# A Proofreading Mutation with an Allosteric Effect Allows a Cluster of SARS-CoV-2 Viruses to Rapidly Evolve

**DOI:** 10.1093/molbev/msad209

**Published:** 2023-09-20

**Authors:** Andrew H Mack, Georgina Menzies, Alex Southgate, D Dafydd Jones, Thomas R Connor

**Affiliations:** Molecular Biosciences Division, School of Biosciences, Cardiff University, UK; Molecular Biosciences Division, School of Biosciences, Cardiff University, UK; Molecular Biosciences Division, School of Biosciences, Cardiff University, UK; Molecular Biosciences Division, School of Biosciences, Cardiff University, UK; Molecular Biosciences Division, School of Biosciences, Cardiff University, UK; Pathogen Genomics Unit, Public Health Wales NHS Trust, Cardiff, UK

## Abstract

The RNA-dependent RNA polymerase of the severe acute respiratory syndrome coronavirus 2 virus is error prone, with errors being corrected by the exonuclease (NSP14) proofreading mechanism. However, the mutagenesis and subsequent evolutionary trajectory of the virus is mediated by the delicate interplay of replicase fidelity and environmental pressures. Here, we have shown that a single, distal mutation (F60S) in NSP14 can have a profound impact upon proofreading with an increased accumulation of mutations and elevated evolutionary rate being observed. Understanding the implications of these changes is crucial, as these underlying mutational processes may have important implications for understanding the population-wide evolution of the virus. This study underscores the urgent need for continued research into the replicative mechanisms of this virus to combat its continued impact on global health, through the re-emergence of immuno-evasive variants.

## Introduction

Severe acute respiratory syndrome coronavirus 2 (SARS-CoV-2) is the causative agent of the coronavirus disease 2019 (COVID-19) global pandemic (Lu et al. 2020). SARS-CoV-2 is a single-strand positive sense RNA virus belonging to the family *Coronaviridae*, genera *Betacoronavirus*, to which the other notable pathogenic human coronaviruses (OC43, HKU1, SARS-CoV-1, and MERS-CoV) also belong (Lu et al. 2020). One important aspect of the behavior of all viruses is their capacity for genetic diversity through mutations. Mutations can arise from errors during the replication of the viral genome, and they have the potential to alter the properties of a virus in various ways. These changes can range from having no effect, through to altering the severity of the virus, rendering it resistant to antiviral drugs, or influencing its transmissibility.

RNA viruses, like SARS-COV-2, have an estimated mutation rate of between 10^−5^ and 10^−2^ substitutions/per site/per year (s/s/y) (Duffy et al. 2008), which is significantly higher than that observed in DNA viruses which typically have mutation rates in the order of 10^−8^–10^−6^ s/s/y (Sanjuán et al. 2010; Peck and Lauring 2018). In comparison with organisms with DNA-based genomes, RNA viruses of the *Coronaviridae* family do not utilize high-accuracy polymerases. Instead, they utilize the RNA-dependent RNA polymerase (RdRp), which, on its own, operates at a faster pace but with a significantly higher error rate compared with most viruses (Shannon et al. 2020). Therefore, coronaviruses employ an independent proofreading mechanism in the form of an exonuclease to correct errors introduced by the RdRp. The exonuclease of SARS-CoV-2 is housed within the nonstructural protein 14 (NSP14) coding segment, along with an N7-guanine methyltransferase. This exonuclease serves a crucial function in maintaining the accuracy of the viral RNA genome by removing erroneous nucleotides during replication. This process helps reduce the rapid accumulation of mutations which could be deleterious, ensuring that most virions generated are viable, and allows the virus to maintain its relatively large genome, which is ∼30 kb in size, while still functioning efficiently (Gorbalenya et al. 2006; Ogando et al. 2020; Jo et al. 2021). Even with the proofreading capabilities of the exonuclease, SARS-CoV-2 has been able to adapt to the human host through the acquisition of mutations, exploring evolutionary space in a stepwise way. The rate of the emergence of variants/advantageous mutations is partly a function of the mutation rate of the virus, which is itself a function of the interaction of NSP14 and RdRp. It is also important to note that although mutations operate to generate new diversity in the population, coronaviruses are also able to undergo recombination, which serves to shuffle diversity within the wider population and has already been seen in SARS-CoV-2 (Jackson et al. 2021). This diversity can further confer an advantage which can then spread through lateral gene transfer into other contexts. Therefore, it is crucial to comprehend and dissect the processes responsible for mutations in SARS-CoV-2 in order to gain a deeper insight into the factors contributing to the emergence of diversity within the virus.

**Fig. 1.**
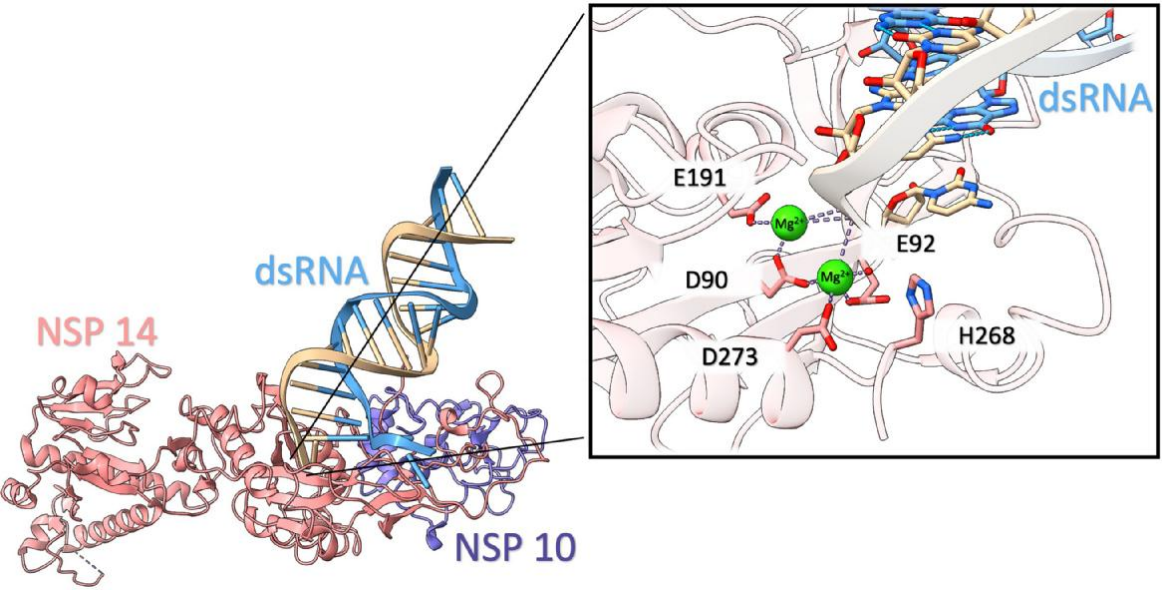
Structure of SARS-CoV-2 NSP14/NSP10 complex with dsRNA. This derives from the high-resolution crystal structure PDB:7N0C, with the E191A mutation reverted. The zoomed-in view of the catalytic core depicts the two Mg^2+^ ions that coordinate with the residues of the enzyme, providing insight into the key elements that facilitate the exonuclease activity of the NSP14. This figure provides a visual representation of the critical components of the complex and its interaction with dsRNA (PDB:7N0C).

The exonuclease core of NSP14 within SARS-CoV-2 is a DEEDh enzyme and member of the DEED superfamily (Zuo and Deutscher 2001). The active site residues are comprised of two glutamic acid residues (E92 and E191), two aspartic acid residues (D90 and D273), and a single histidine residue (H268) which has been structurally confirmed and is similar to the NSP14 of SARS-CoV-1 (Ma et al., 2015; Liu et al. 2021; Lin et al. 2021). The excision of mis-incorporated nucleotides which are introduced through replication is facilitated by two coordinated Mg^2+^ ions (fig. 1). Like other exonucleases, the first Mg^2+^ ion (which is coordinated by E92 and D273) activates a water molecule to initiate nucleophilic attack, whereas the second Mg^2+^ ion (coordinated by D90 and E191) helps to remove the erroneous nucleotides from the replication complex (Hwang et al. 2018). Thus, coordination of Mg^2+^ ions play a critical role in maintaining the accuracy of the viral RNA genome (Ma et al., 2015).

Inactivation of the exonuclease enzyme of HCoV-229E and MERS-CoV through site-directed mutagenesis of key catalytic residues has previously been shown to prevent the successful generation of replication competent, viable virions (Minskaia et al. 2006; Ogando et al. 2020). However, although site-directed mutagenesis experiments of the DEEDh core of HCoV-229E and MERS-CoV rendered these viruses inviable, similar experiments conducted upon SARS-CoV-1 and murine hepatitis virus (MHV) produced viable virions, but with their fidelity greatly compromised, with progenitors displaying a significantly increased burden of mutations with an accompanying fitness cost (Eckerle et al. 2007, 2010; Graepel et al. 2017). Elsewhere, others have recognized that general mutations within the exonuclease are associated with an increased burden of mutations upon the SARS-CoV-2 viral genome (Eskier et al. 2020), but the underlying functional cause was not explored. In addition to its proofreading ability, the exonuclease has also been shown to be crucial for native recombination, as its inactivation resulted in a significant decrease in recombination frequency within MHV (Gribble et al. 2021), further highlighting the diverse roles of this enzyme.

In vitro reverse genetics experiments have shown that the SARS-CoV-1 exonuclease activity is mediated by its interaction with NSP10 (Bouvet et al. 2014). This work illustrated how conserved mutations of residues at the interface of the exonuclease and NSP10 within SARS-CoV-1 not only reduced exonuclease activity but were also capable of rendering the virus severely attenuated or nonviable. Enzymatic characterization experiments have recognized the significance of the NSP14/NSP10 interaction in modulating exonuclease activity, with the complex showing a >35-fold increase in activity compared with the NSP14 alone (Bouvet et al. 2012). This modulation of replication fidelity was further observed in vitro within a MHV model, where mutations at the NSP14/NSP10 interface led to an inability to excise the nucleotide analogues 5-azacytidine and ribavirin (Smith et al. 2015).

Considering the above, this investigation sought to explore whether mutations at the interface of the NSP14 and NSP10 resulted in demonstrable effects upon the evolutionary trajectory of the SARS-CoV-2 virus. Already, the global SARS-CoV-2 sequence data sets provide an unprecedented resource of genome sequence data that enables the identification of clusters of cases, variants of concern (VoC), and an estimation of their observed evolutionary rates. The large, global data set provides an opportunity to examine the generation of diversity in the SARS-CoV-2 population at unprecedented resolution. Building on the efforts of the global scientific community has allowed us to identify a cluster of SARS-CoV-2 cases that possess an interface mutation and to quantify that these viruses exhibit an elevated evolutionary rate. Using these mutations as a starting point, we undertook further in silico investigations to determine their potential impact on exonuclease function through predictive modeling, molecular dynamics (MD), and residue interaction network analyses, which have emerged as powerful tools in the investigation of the effect of mutations on replicase enzymes, including exonuclease function (Berger and Cisneros 2023). By modeling the structural dynamics of the exonuclease, we were able to determine whether mutations could lead to a distal allosteric effect on the catalytic residues of the enzyme, contributing to the observed higher mutation rate. The in-depth examination provided by these simulations offered crucial insights into the complex interaction between the mutations and the protein structure, corroborating our evolutionary findings and providing a more comprehensive understanding of the underlying mechanisms.

## Results

### Identifying Sequences with Mutations at the NSP14/NSP10 Interface

**Fig. 2.**
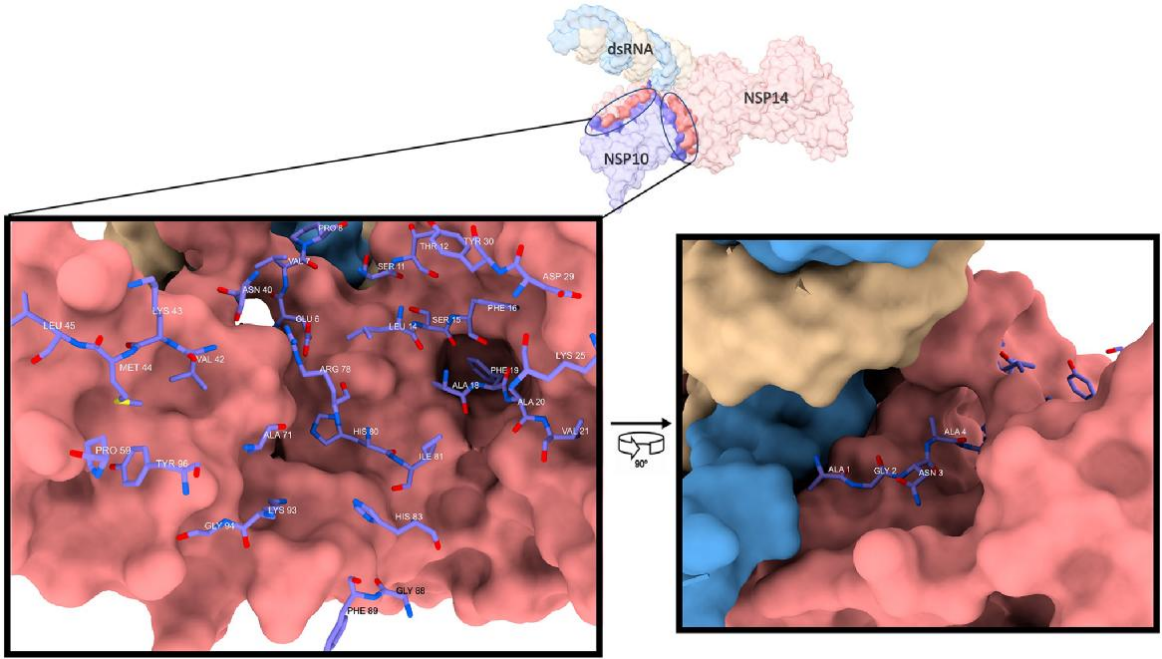
NSP10 and NSP14 interaction in SARS-CoV-2—a representation of the localization of NSP10 residues to the surface model of NSP14 (PDB:7N0*C*).

An examination of the NSP14/NSP10 interface through network analysis (as shown in supplementary fig. 1, Supplementary Material online) uncovered interactions between 29 residues from NSP10 and 32 residues from NSP14. These interactions mainly consisted of Van der Waals forces (40 in total), with additional contributions from 8 hydrogen bonds, 4 Pi–Pi interactions, and 1 ionic bond. Prior research has noted two specific Pi–Pi interactions, between F16 of NSP10 and Y64 of NSP14 and F60 of NSP14 and F19 of NSP10, which have been considered as a possible therapeutic target due to their potential allosteric impact upon the catalytic core (Saramago et al. 2021) (fig. 2 shows the localization of NSP10 residues with NSP14). After identifying all the residues at the interface, a search was conducted on the Global Initiative on Sharing All Influenza Data (GISAID) (Shu and McCauley 2017) database to find SARS-CoV-2 sequences with mutations at those sites. As a result, 14 groups of SARS-CoV-2 sequences with mutations at the interface were discovered. Groups are subsequently named by the interface mutation they possessed. Additionally, two control lineages were obtained that did not posess any mutations at the NSP14/NSP10 interface. The complete list of data sets generated for this study, along with the final number of samples in each, is presented in supplementary table S1, Supplementary Material online.

### Temporal Signal and Bayesian Evolutionary Analysis

**Fig. 3.**
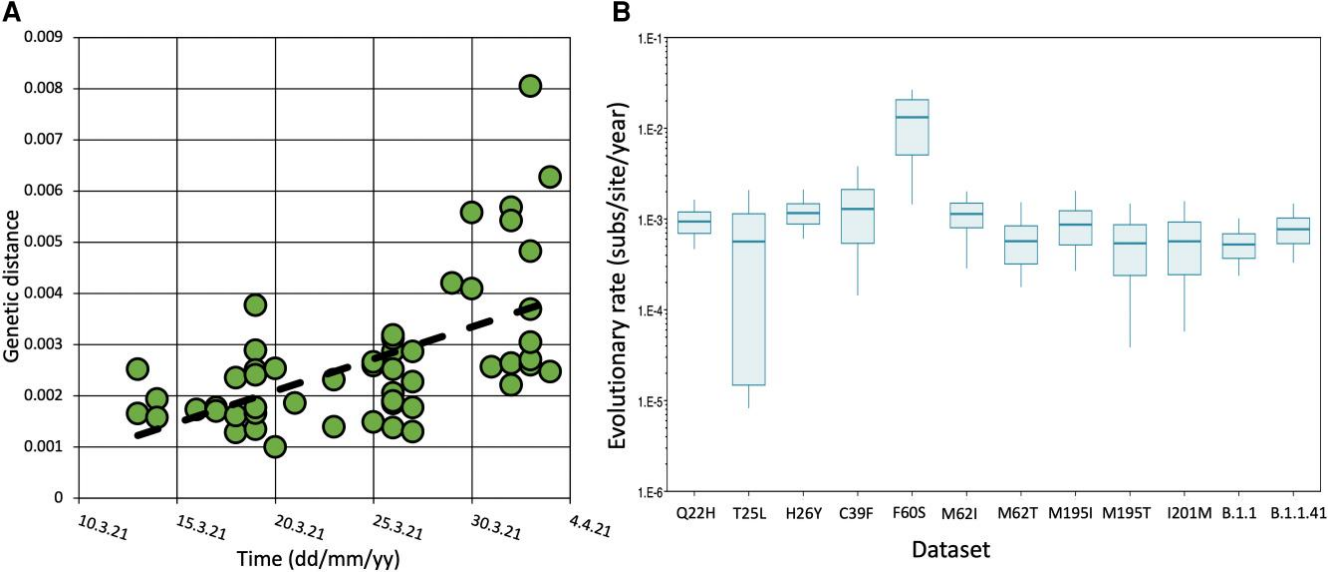
(*A*) Root-to-tip regression plot of the 55 F60*S* sequences showing genetic distance versus time (in dd/mm/yy). (*B*) Posterior distribution of the evolutionary rate (s/s/y) of each data set analyzed under the best ranking model using BEAST.

As an initial assessment of a temporal signal resembling a molecular clock within the identified control and mutant lineages, a linear root-to-tip regression was performed using TempEst v1.5.3 (Rambaut et al. 2016). This step was taken to verify that the data sets were suitable for further Bayesian analysis. Maximum likelihood phylogenies were created for each data set, highly divergent genomes were removed, and data set was rebuilt. A temporal signal was observed in all data sets tested, with rates ranging from 1.86 × 10^−4^ to 2.63 × 10^−3^ (supplementary table S2, Supplementary Material online). The *R*^2^ value, which indicates the degree to which a sample is “clocklike,” also showed variability. For example, *R*^2^ for the M62I mutant was 0.83, whereas F60S had a much lower *R*^2^ of 0.007, indicating a departure from clocklike behavior and a more variable rate of evolution. A root-to-tip regression for the F60S data set is shown in figure 3*A*, whereas a complete data set of root-to-tip results is presented in supplementary table S2, Supplementary Material online. Having detected an initial temporal signal in all data sets, they were all subjected to further Bayesian analysis using BEAST v.1.10.4 (Suchard et al. 2018) to estimate their evolutionary rates. The most appropriate clock model for each data set was first determined through path sampling and stepping stone (SS) analysis (supplementary table S3, Supplementary Material online). All other priors were kept consistent across data sets, including the tree prior, as it was believed to have limited effect on the final molecular clock estimates (Ritchie et al. 2017). Once the best model for each data set was identified, an additional and more robust test of temporal signal was conducted. A Bayesian evaluation of temporal signal (BETS) (Duchene et al. 2020) was performed on each data set, where the aforementioned BEAST analysis was repeated; however, sampling dates were removed. This analysis revealed that data sets H26N, M57I, M57V, and M62V possessed either a weak or no temporal signal, with a Bayes factor <1, and consequently, any downstream inference of these data sets would not be reliable and were removed from the study (supplementary table S6, Supplementary Material online). Evolutionary rates of SARS-CoV-2 are commonly estimated to be within the range of 7.0 × 10^−4^ s/s/y (Ghafari et al. 2022). In this study, we found the evolutionary rate of our two comparator, nonmutant groups to also reside within or close to this estimate; we subsequently name these groups controls. Control group 1 (B.1.1 lineage) resulted in an s/s/y rate of 5.27 × 10^−4^ [95% highest posterior density (HPD) 3.72 × 10^−4^–6.92 × 10^−3^), and Control group 2 (B.1.1.41) displayed an s/s/y rate of 7.71 × 10^−4^ (95% HPD 5.35 × 10^−4^–1.02 × 10^−3^). In comparison, certain data sets containing mutations at the NSP14/NSP10 interface deviated strongly from this expected rate, particularly the F60S and C39F data sets, which resulted in s/s/y rates of 2.37 × 10^−2^ (95% HPD 8.03 × 10^−3^–3.83 × 10^−2^) and 4.06 × 10^−3^ (95% HPD 7.55 × 10^−4^–8.57 × 10^−3^), respectively. The posterior distribution of the evolutionary rates from the BEAST analyses is shown in figure 3*B*. Furthermore, both data sets showed a coefficient of variation which had departed from and was no longer abutting zero, exemplifying how the evolutionary rate of both sets of genomes have departed from clocklike behavior, exhibiting variation between branch rates. We recognize that data sets containing fewer sequences tend to align with broader 95% HPD intervals, implying a reduced level of certainty in the estimates—as expected. Table 1 presents the results of the Bayesian analysis for all data sets.

**Table 1.** Results of BEAST Analysis.

Data Set	Number of Sequences	Clock Model	Mean Rate (s/s/y)	95% HPD (s/s/y)
Q22H	100	E relaxed	9.38 × 10^−4^	6.42 × 10^−4^–1.25 × 10^−3^
T25L	24	E relaxed	5.98 × 10^−4^	4.17 × 10^−5^–1.40 × 10^−3^
H26Y	100	LN relaxed	1.26 × 10^−3^	7.24 × 10^−4^–1.91 × 10^−3^
C39F	54	LN relaxed	4.06 × 10^−3^	7.55 × 10^−4^–8.57 × 10^−3^
F60S	55	E relaxed	1.36 × 10^−2^	8.51 × 10^−3^–3.83 × 10^−2^
M62I	102	LN relaxed	1.58 × 10^−3^	7.11 × 10^−4^–2.84 × 10^−3^
M62T	100	E relaxed	6.07 × 10^−4^	3.25 × 10^−4^–9.16 × 10^−4^
M195I	54	E relaxed	1.01 × 10^−3^	5.36 × 10^−4^–1.53 × 10^−3^
M195T	55	E relaxed	6.34 × 10^−4^	2.40 × 10^−4^–1.08 × 10^−3^
I201M	112	E relaxed	6.28 × 10^−4^	2.91 × 10^−4^–1.00 × 10^−3^
Control 1 B.1.1	100	Strict	5.27 × 10^−4^	3.72 × 10^−4^–6.92 × 10^−3^
Control 2 B.1.1.41	77	Strict	7.71 × 10^−4^	5.35 × 10^−4^–1.02 × 10^−3^

### Detection of Previous Recombination Event within the F60S Lineage

Although the C39F mutant exhibited a notably elevated evolutionary rate, the rate observed in the F60S data set was considerably higher and became the focus in downstream analyses. To elucidate the possible cause for this dramatic elevation in the observed evolutionary rate, a test of recombination was performed. Using RDP5 (Martin et al. 2021), an initial analysis was conducted within the F60S data set, which did not yield any evidence of recombination. Following this, a second analysis was undertaken where 3,000 genomes from throughout the pandemic were obtained from GISAID. For computational efficiency, this data set was split into 10 tranches (∼300 sequences per tranche) with the F60S data set being added to each tranche followed by another test of recombination using RDP5. From this analysis, results indicated that a recombination event was likely present within the F60S genomes, which was supported by 6/9 methods deployed in RDP5 (supplementary table S4, Supplementary Material online); these included MaxChi (*P* = <0.05), 3Seq (*P* = <0.01), RDP (*P* = <0.001), GENECOV (*P* = <0.001), and BootScan (*P* = <0.0001). The recombination breakpoints were suggested to be at nucleotides 21,742 and 23,666 (codons 61–705 of the spike protein), with the inferred (unknown) major parent being from the B.1.1.529 lineage (Omicron) and minor parent being from the B.1.1 lineage, suggesting the B.1.1 minor parent had donated its S1 domain of the spike protein during this recombination event (supplementary fig. 2, Supplementary Material online). Although the F60S genomes were recombinant, this was not considered to be the cause of the elevated rate, as this event was basal to data set that was examined using BEAST, and therefore, it would have no bearing upon this analysis. However, this does highlight that these genomes, with a higher detectable rate of mutation, do have a history of recombination, pointing to the potential for these two mechanisms to combine to generate significant diversity within the population.

### MD Simulations of F60S and Wild-Type NSP14/NSP10 Complexes

Having established that there was no evidence pointing toward intra-dataset recombination as a cause of the elevated rate, we sought to specifically examine if the F60S mutation had any effect upon the NSP14/NSP10 protein complex of SARS-CoV-2. To examine whether F60S may influence its behavior, MD simulations were performed. The source structure for the MD was PDB:7N0C. This structure possessed the mutation E191A at the catalytic core for experimental purposes; this mutation was reverted to wild-type glutamate using CHARMM-GUI (Jo et al. 2008), which was also used to introduce the F60S mutation to the mutant starting structure. Simulations were ran for 100 ns with three repeats. Root mean squared deviation (RMSD) analysis suggested all simulations were stable (supplementary fig. 3, Supplementary Material online). An analysis of the F60S NSP14/NSP10 complex revealed the *N*-terminus of NSP10 that interacts with double-stranded RNA, and NSP10 was more flexible, but the stability between wild-type and mutant RNA was comparable. Within NSP14, although changes in root mean squared fluctuation (RMSF) were observed, particularly an increase in flexibility between residues 221 and 231, no significant changes were observed at the catalytic core (supplementary fig. 4, Supplementary Material online).

### Changes in the Residue Interaction Networks at the F60S Mutation Site

**Fig. 4.**
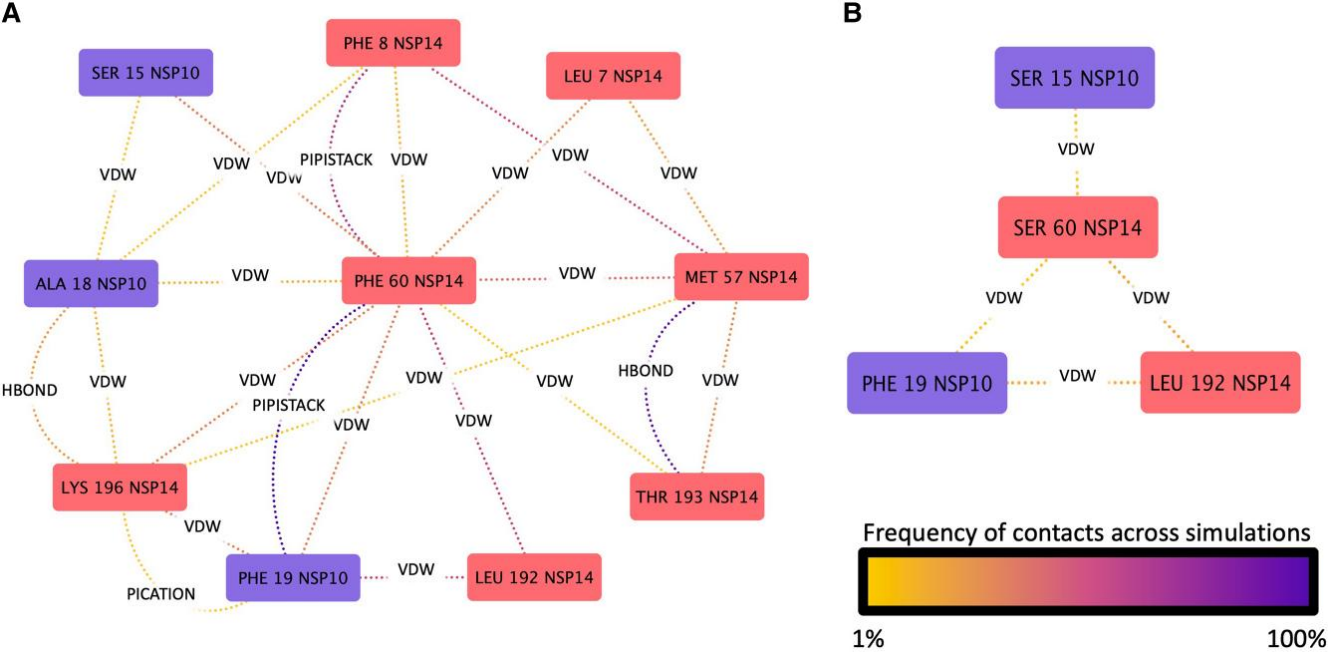
The interaction of the PHE60 of NSP14 in the wild-type (*A*) structure and SER60 in the mutant structure (*B*) and their respective interaction networks. Types of interactions are labeled, with the color of the dotted lines depicting the frequency of those interactions averaged over the MD simulations (VDW, Van der Waals interactions; HBONDS, hydrogen bonds; PIPISTACK, Pi–Pi stacking; IONIC, ionic bond; PICATION, Pi–cation interaction).

To better illustrate the changes in the local interactions within the proximity of the F60S mutation, the three separate 100 ns trajectories were concatenated to single 300 ns simulation for both the wild-type and mutant. Six hundred frames (20% of total) from each concatenated simulation were then extracted, and a residue interaction network was created using RING3.0 (Clementel et al. 2022). This network analysis clearly illustrates the local changes between the wild-type and mutant structures (fig. 4), where the F60S mutation led to the loss of seven out of the ten residue interactions seen in the wild-type and a dramatic decrease in the frequency of the remaining interactions.

### Predicting the Effect of F60S Mutation on the Binding Affinity of NSP14/NSP10 and Interface Surface Size

To determine the impact of the F60S mutation on the binding strength of NSP14 with NSP10, multiple structure-based tools including I-mutant, DynaMut2, CUPSAT, mCSM-PPI2, and mCSM were used. By utilizing these different methods, a consensus can be reached on the calculation of this change in binding affinity. All methods predicted that the NSP14/NSP10 complex would be destabilized because of this mutation, with ΔΔG (difference in Gibbs free energy) ranging between −1.12 and −3.32 kcal/mol (supplementary table S5, Supplementary Material online). This change in affinity may disrupt communication networks across the complex, resulting in a disruption of allosteric signaling. The interface surface size was calculated using the Protein Interfaces, Surfaces and Assemblies (PISA) tool (Krissinel and Henrick 2007) which revealed a surface interface size of 2,256 Å^2^ and energy of −24.3 kcal/mol.

### Mapping of Eigenvector Centrality and Changes in Catalytic Residue Interactions to Illuminate Allosteric Effects

**Fig. 5.**
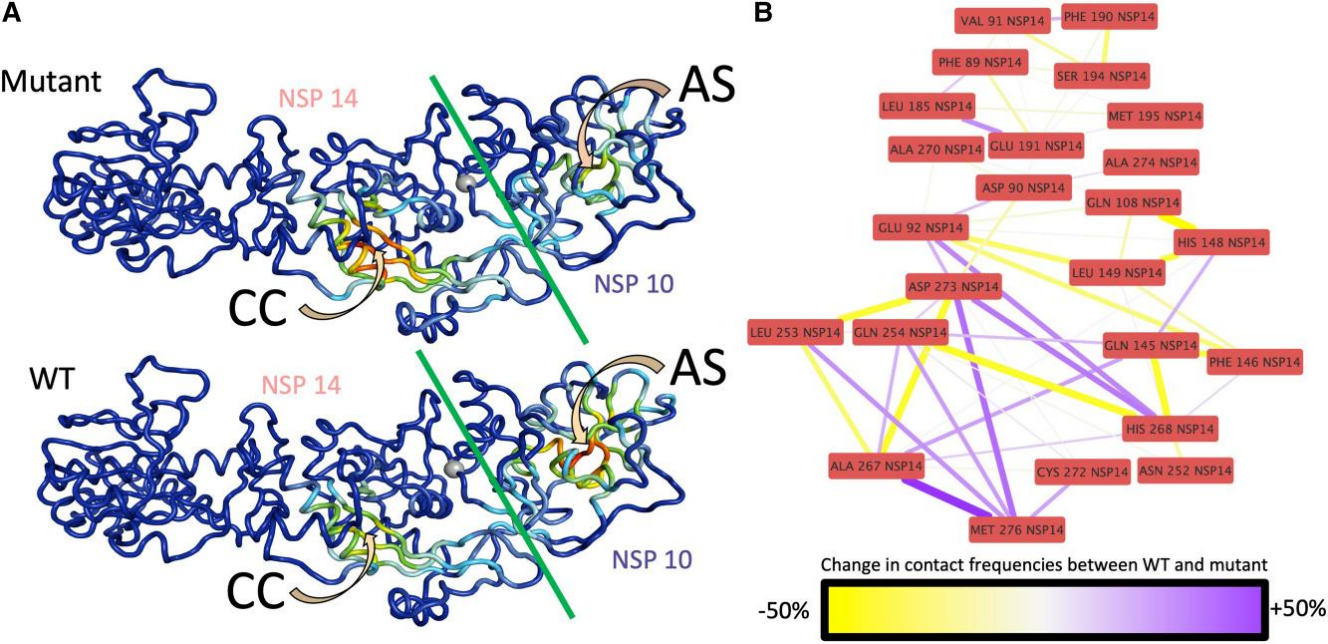
(*A***)** Eigenvector centralities projected onto wild-type (bottom) and mutant (top) NSP14/NSP10 complexes. Here, the centrality of each residue is depicted by color, with blue showing low centrality and red showing high centrality. CC depicts the catalytic core, whereas AS identifies a possible allosteric site. The silver sphere is the F60*S* mutation site. The green line is the between NSP14 and NSP10. (*B*) Change in contact frequencies between residues within 4.5 Å of catalytic residues.

Identifying the pathways responsible for allosteric communication in biomolecules is a difficult task, partly due to the intricate nature of these systems and the absence of effective characterization methods. However, this was previously achieved through exploiting eigenvector centrality, which in essence is a measure of the importance of, or contribution of, information from a node (in this instance a residue) to the remainder of the network (Negre et al. 2018). From MD simulations, nodes and edges can be derived from residues and interactions with well-connected/interacting nodes associated with high eigenvector centralities. Changes in these centralities can provide insight to system-wide changes which may have been brought about by mutations. Here, the *correlationplus* software (Tekpinar et al. 2021) was used to perform this analysis and identify eigenvector centralities within the wild-type and mutant NSP14 structures. Results for the wild-type show a focused eigenvector centrality within the NSP10 (fig. 5*A*), with weaker signals being detected at the catalytic core of the NSP14. However, within the mutant structure, the eigenvector centrality is now focused within the catalytic core of the NSP14, evidencing a reverse in eigenvector centrality. The shift in eigenvector centrality from the middle of the NSP10 protein to the catalytic core in the mutant structure suggests the interaction between NSP10 and NSP14 has altered in such way that affects the connectivity of distal regions of the complex. Differences were also observed between the wild-type and mutant structures in terms of residue contact frequencies at the catalytic core (fig. 5*B*). Here, we show that numerous residue–residue interactions throughout the catalytic core had changed in frequency, with increased interactions between E92, D273, and H268 and reduced interactions between E92, L149, and F146, further exemplifying changes at the network level within the system.

### Characterizing Structural Changes to Catalytic Residues

**Fig. 6.**
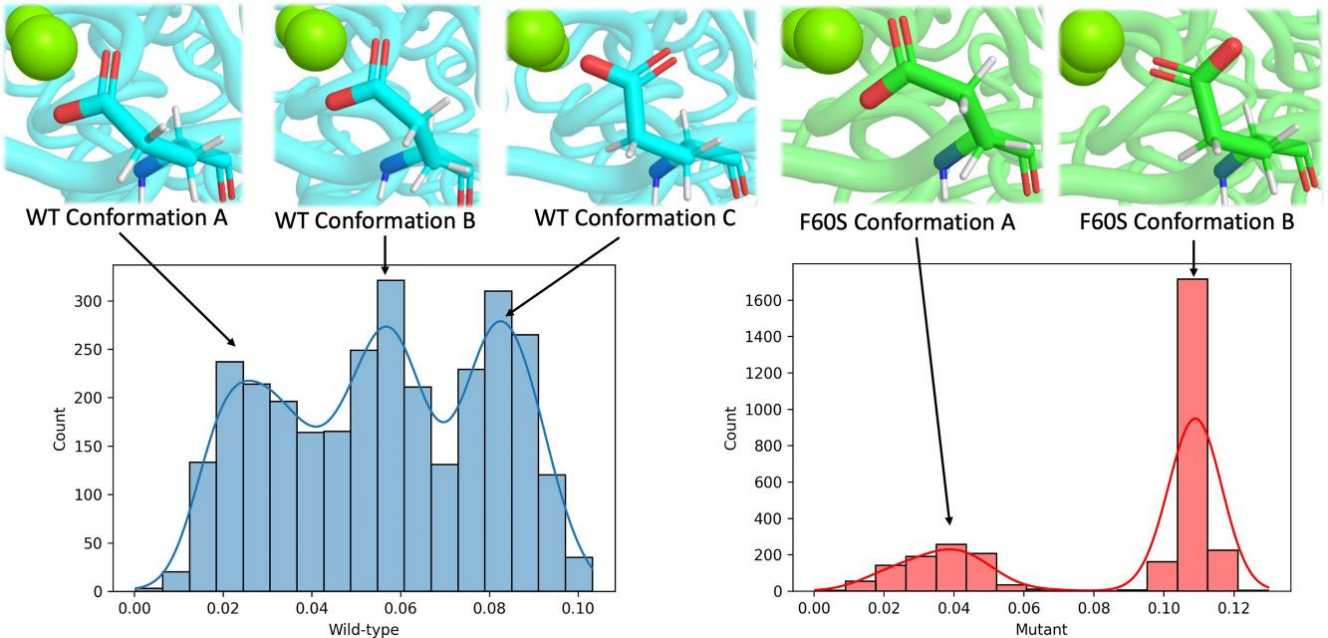
Comparison of the rotamer conformation distributions in the wild-type (left) and F60*S* mutant (right) structures, with representative frames from MD trajectory (top).

Considering the intensification of eigenvector centrality within the catalytic core of NSP14 and changes to the residue interaction network, we next sought to determine whether any structural changes that might impede function could be elucidated by further analyzing the MD data. It has previously been shown that the stability and flexibility of catalytic residues can be characterized through RMSD analysis, which can then show the population distribution of rotamer conformations (Riziotis et al. 2022). This investigation revealed that the distribution of catalytic residue conformations for D90, E191, D273, and H268 was similar between the wild-type and mutant structures across the 3 × 100 ns MD simulations (supplementary fig. 5, Supplementary Material online). However, the distribution of rotamer conformations for E92 in the mutant structure showed a significant departure from the wild-type distribution (fig. 6). The wild-type showed three distinct populations of conformations (wild-type conformations A, B, and C), but analysis showed that E92 of the F60S mutant structure primarily resided in a single conformation (F60S conformation B) with very brief periods in conformation A. Conformation A of the F60S simulation is comparable with conformation B of the wild-type simulation; however, conformation B of the F60S simulation is unique and not observed in the wild-type simulation, which sees the carboxyl group of E92 rotate 180 degrees. This may indicate that the intrinsic flexible and dynamic quality of the wild-type E92 is no longer present within the mutant structure.

## Discussion

The exonuclease within the NSP14/NSP10 complex plays an important role in maintaining the accuracy of replication by removing erroneous nucleotides introduced by RdRp. Examining the global SARS-CoV-2 genomic data set, we were able to identify clusters of cases which shared a mutation in NSP14 exonuclease that exhibited a detectable increase in evolutionary rate compared with other contemporaneous lineages.

The formation of the NSP10/NSP14 complex is known to promote exonuclease activity (Bouvet et al. 2014) and plays a key role in maintaining fidelity during viral RNA replication. The NSP10/NSP14 interface is relatively extended covering ∼2,250 Å^2^ and a predicted interface energy of −24.3 kcal/mol. Thus, a single mutation like F60S with a predicted interface energy loss of −1.1 to −3.2 kcal/mol is unlikely to lead to total complex disassembly.

The F60S mutation is anticipated to influence the exonuclease activity and evolutionary pace of the SARS-CoV-2 virus via an allosteric mechanism triggered by modifications in the interaction network within the NSP14/NSP10 interface. Although it is probable that this complex will stay structurally intact, the mutation is expected to modify the surface area and perturb the allosteric communication between the interface of the complex and the active site of NSP14. Figure 1 provides a visual representation of the active site's positioning in relation to the NSP14/NSP10 interface.

Our modeling suggests that the NSP14 F60S mutation results in a significant reduction in the number of residue–residue interactions within itself and NSP10. Although the latter is to be expected given its site at the interface and this is borne out in a slight reduction in predicted affinity, the reduction in interactions within NSP14 is likely to be more significant due to the extensive number of reduced contacts. Additionally, there was a notable transference in eigenvector centrality following the introduction of the F60S mutation. This became focused within the catalytic core of the mutant NSP14 as opposed to the wild-type NSP10, signifying a change in the in the epicenter of the communication network across the complex. Furthermore, an altered coordination of the catalytic core of the F60S mutant was characterized by the dramatic change in the distribution of rotamer conformations of the critical catalytic residue E92.

Simulations of the wild-type structure showed three distinct populations of conformations for E92, whereas the mutant structure primarily resided in a single conformation with only a brief period in one other conformation. This departure from the wild-type conformation distribution suggests that the intrinsic flexibility and dynamic quality of the wild-type E92 is no longer present in the mutant structure.

The change in the distribution of rotamer conformations for E92 of the catalytic core within the NSP14 would likely impact the function of the exonuclease, as one of the key roles of E92 is the accurate coordination of an Mg^2+^ ion, essential for the exonucleolytic reaction (Hwang et al. 2018). The catalytic core plays a critical role in the RNA hydrolysis process, and any changes to its stability and flexibility could have consequences on the efficiency of its exonucleolytic reaction.

Mutations in the catalytic core of NSP14 have previously been linked to an increased accumulation of mutations in certain coronaviruses (Eckerle et al 2010; Graepel et al. 2017; Niu et al. 2021). Similarly, our study has demonstrated that the F60S mutant displays an increase in the rate with which it acquires mutations, a potential indication of a nonoptimal exonuclease.

The NSP14/NSP10 complex's function may be significantly impacted by the decrease in the number of residue interactions and binding strength at the interface, as well as the suggestive shift in the allosteric network and changes in rotamer conformation. If the binding affinity and/or the coupled residue interaction network between NSP14 and NSP10 decrease, the complex's allosteric communication networks could be disrupted, leading to a disruption of the exonuclease's proper functioning.

Moreover, it's worth noting that the observed evolutionary rates in this study are quite remarkable, surpassing those documented in other research efforts identifying lineages with heightened evolutionary rates (Hill et al. 2022; Tay et al. 2022). Although the mechanisms leading to an increased rate varies between these studies and the analysis of the F60S mutant in question, it is probable that selective host pressures exert an influence in all cases.

Here, we suggest that the F60S lineage experienced mutational meltdown, leading to its extinction within 22 days of detection. However, prior to its demise, the high mutation rate allowed these viruses to explore the sequence space and acquire mutations associated with improved fitness and increased virulence. Through an analysis using USHER (Turakhia et al. 2021) (supplementary table S7, Supplementary Material online). We identified several spike protein mutations, uncluding 11 nonsynonymous mutations known to evade immune responses, improve receptor binding, or enhance transmissibility. Forty-four other nonsynonymous mutations were also detected within this lineage; though their significance remains uncertain, they could be of no effect or detrimental. We suggest that the F60S mutant eventually crossed a fitness threshold where the negative impact of detrimental mutations surpassed the benefits gained from advantageous mutations, resulting in cessation due to mutational meltdown.

In conclusion, this study has leveraged phylodynamic investigations to identify altered mutational rates within specific genotypes of SARS-CoV-2, which were then structurally assessed using MD approaches. Having identified F60S as a mutation of interest, our analysis then highlighted the impact of the NSP14 F60S mutation on the structural and functional integrity of the NSP14/NSP10 complex in SARS-CoV-2. The reduction in binding strength, shift in allosteric network, and changes in rotamer conformation could result in a reduction in the efficiency of the exonucleolytic reaction and negatively impact the replication fidelity of the virus, potentially providing an explanation for the increased evolutionary rate observed through the Bayesian analysis.

The mechanisms by which SARS-CoV-2 and other *Coronaviridae* generate diversity remain an important puzzle to decipher. The ability of SARS-CoV-2 to shuffle diversity via recombination has already been shown (Jackson et al. 2021), whereas the role of chronic carriers has also been identified as one mechanism by which SARS-CoV-2 may generate significant amounts of diversity, allowing “jumps” between variants (Hill et al. 2022). Furthermore, zooanthroponosis (reverse zoonotic) SARS-CoV-2 infections in white-tailed deer (Pickering et al. 2022) and mink (Porter et al. 2023) have also been associated with increased evolutionary rates of 3.7 × 10^−3^ and 6.59 × 10^−3^ respectively, most likely as a result of significant selective pressures brought about by novel host environments.

This work potentially identifies another route by which mutations can be generated rapidly, which, when combined with the ability of SARS-CoV-2 to recombine, may be of significance in the evolution of SARS-CoV-2 going forward. It also demonstrates an interesting and potentially important observation, but further studies are now needed to fully understand the impact of exonuclease mutations and their potential impact on the generation of diversity in SARS-CoV-2 and other viruses.

## Methods

### Whole Genome Sequences Retrieval and Preparation

A search of the GISAID database was undertaken in November 2022 to identify clusters of SARS-CoV-2 viruses with mutations at the NSP14/NSP10 interface which were previously highlighted. Sequences with low coverage and missing collection date were excluded. Sequences were download and allocated a data set only when >20 sequences could be attributed to a single PANGO lineage (O’Toole 2021). Each experimental data set was named in accordance with the mutation possessed at the NSP14/NSP10 interface. Two control lineages were also obtained from GISAID which did not contain mutations in either the catalytic core of the exonuclease or the NSP14/NSP10 interface. Each data set was checked for monophylogeny using USHER (Turakhia et al. 2021) to ensure close clustering with outliers and genetically distant sequences being removed. A list of Spike protein mutations was also obtained from USHER (supplementary table S7, Supplementary Material online). Each data set was aligned to the SARS-CoV-2 reference genome MN908947.3 using MAFFT (Katoh and Standley 2013) with --keeplength --6merpair --addfragments options. Highly variable ends and troublesome sites were masked as described elsewhere (De Maio et al. 2020). The GISAID accession numbers for all sequence are available in supplementary materials.

### Temporal Signal and Bayesian Analysis

Maximum likelihood phylogenies were constructed with IQ-TREE (Trifinopoulos et al. 2016) using the GTR model (Tavaré 1986). A temporal signal was determined using TempEst v.1.5.3 (Rambaut et al. 2016) with *R*^2^ and correlation coefficients being generated under the heuristic residual mean squared function with a best fitting root applied. Evolutionary analysis was undertaken to determine the number of s/s/y using BEAST v.1.10.4 (Suchard et al. 2018). Three different models were evaluated for each group. These models were composed of three different clock models: strict, lognormal uncorrelated relaxed, and exponential uncorrelated relaxed (Drummond et al. 2006). In each model, an exponential coalescent tree prior was implemented (Drummond et al. 2002) which is not expected to have an excessive impact on substitution rates while allowing the models to be more comparable (Ritchie et al. 2017). Each model also used a GTR+Γ4 substitution model. Population size was set to an exponential distribution with a mean of 10^5^, whereas for the growth rate, a Laplace distribution with a location of 0 and a scale of 100 was chosen. For the lognormal relaxed clock models, an exponential prior of 0.33 (mean) was set. A continuous-time Markov chain (CTMC) prior was set as the clock rate for each model (Ferreira and Suchard 2008). Each model was run in triplicate for chain lengths of 10^8^, sampling every 10^3^ iterations. The best model was identified following the comparison of log-marginal likelihood estimates generated by SS analysis (Baele et al. 2012, 2013). The SS analysis was performed for 200 steps with chain lengths being set to 10^6^, and values were logged at every 1,000th iteration. 10% of results were discarded and considered burn in. Triplicate runs were combined using LogCombiner and Markov chain Monte Carlo (MCMC) traces were reviewed using Tracer v1.7.1 (Rambaut et al. 2018) to check for convergence and that effective sample sizes were >200. The best model was reran for each data set without tip-dates as a formal test of temporal signal (Duchene et al. 2020), and log-marginal likelihoods of data sets containing tip-dates and those without were then directly compared. Where a Bayes factor <1 in favor of the tip-date containing data set was reported, that data set was regarded as having a weak temporal signal and was removed from the study as inference would be unreliable. The data sets that were removed were H26M, M57I, M57V, and M62V (full BETS results are provided in supplementary table S6, Supplementary Material online). Finally, for those data sets with a strong temporal signal, an additional BEAST analysis was performed, sampling only from the prior. The results for this analysis are presented in supplementary figure 6, Supplementary Material online. Considering difference between the posterior densities of the prior-only and fully informed runs, it can be assumed that the results are not solely derived from the influence of the predetermined priors.

### Detection of Recombination

In May 2022, a globally representative data set of ∼3,000 SARS-CoV-2 whole genome sequences was obtained from GISAID. This data set was downloaded from the Genomic Epidemiology Portal which houses the preselected sequences which were considered being globally representative. Once downloaded, the ∼3,000 sequence data set was separated to 10 tranches of ∼300 sequences for computational efficiency. To each subset, the F60S mutant sequences were added, and as done previously, each tranche was aligned to the SARS-CoV-2 reference genome MN908947.3 using MAFFT (Katoh and Standley 2013) with --keeplength --6merpair --addfragments options. Again, highly variable ends and troublesome sites were masked as described elsewhere (De Maio et al. 2020). This produced 10 tranches of ∼300 sequences of globally representative sequences combined with the F60S sequences. Each tranche was tested for recombination using RDP5 (Martin et al. 2021) with the following methods RDP, GENECOV, BootScan, MaxChi, Chimera, SiScan, PhyloPro, LARD, and 3Seq. All methods were used using default settings. The GISAID accession numbers for the sequences from the recombinant positive tranche are provided in supplementary materials.

### NSP14/NSP10 Interface Analysis

The 7N0C structure of the SARS-CoV-2 NSP14 bound to NSP10, and RNA (Liu et al. 2021) was downloaded from the protein databank (https://www.rcsb.org/). The structure was uploaded to the RING3.0 server (Clementel et al. 2022), and using the default parameters for bond distances, an interaction map was generated. The resulting JSON file was visualized and analyzed in Cystoscape (Shannon et al. 2003).

### MD Analysis and RMSF Analysis

Again, using the 7N0C structure, two molecular systems were prepared for MD simulations with the CHARMM-GUI web interface (Jo et al. 2008). Except for introducing the F60S mutations to the mutant structure as mentioned previously, both systems were set up and ran in the same manner. A water box was made that was 10 Å from the protein edge, and 0.15 M of Na^+^ and Cl^−^ ions were added to neutralize the system charge. Energy terms were derived from CHARMM36 forcefields (Huang and Mackerell 2013), whereas water molecules were represented by TIP3 potentials (Jorgensen et al. 1983). A step size of 0.001 with a 12 Å cutoff was selected to consider nonbonded, distant interactions with a particle mesh Ewald summation (Darden et al. 1993). Hydrogen within covalent bonds were constrained by LINCS, and the system temperature was maintained at 310.15 K (37 °C). System pressure was maintained at 1 bar with a compressibility value of 4.5 × 10^−5^ bar^−1^. Equilibration was performed with a step size of 0.001 ns for 125,000 steps. Minimization was performed with a steep descent integrator for 5,000 steps. The production runs were performed at 0.002 ns step sizes for 50,000,000 steps (100 ns). All simulations were ran using GROMACS 2021.2 (Abraham et al. 2015). RMSD analysis was performed using the gmx rms command with the residue being selected as a reference from a custom index file. RMSF analysis was performed with gmx rmsf using the carbon alpha of the protein system.

### Residue Interaction and Contact Frequency Analysis

The 3 × 100 ns simulations for the wild-type were concatenated as were the simulations for the mutant. One thousand frames were then extracted from each of the concatenated simulations as PDB files. The files were then analyzed with RING3.0 (Clementel et al. 2022) locally. The resulting JSON file was visualized and analyzed in Cystoscape (Shannon et al. 2003). The concatenated PDB files were loaded to Cytoscape where SenseNet (Schneider and Antes 2022) was used to compare the change in contact frequency of residues within 4.5 Å of the catalytic residues.

### Binding Affinity Analysis

Five different servers were used to determine the change in binding affinity: I-mutant (https://folding.biofold.org/i-mutant/i-mutant2.0.html) (Capriotti et al. 2005), DynaMut2 (https://biosig.lab.uq.edu.au/dynamut2/) (Rodrigues et al. 2021), CUPSAT (http://cupsat.tu-bs.de/) (Parthiban et al. 2006), mCSM (https://biosig.lab.uq.edu.au/mcsm/protein_protein) (Pires et al. 2014), and mCSM-PPI2 (https://biosig.lab.uq.edu.au/mcsm_ppi2/) (Rodrigues et al. 2019)

### Calculating Eigenvector Centrality

Eigenvector centralities were calculated using the *correlationplus* software (Tekpinar et al. 2021). As described for the residue interaction analysis, the concatenated PDBs for both the wild-type and mutant structures were used as inputs for this analysis. Here, the dynamic cross correlation (DCC) approach was used with default parameters.

### Structure Illustrations

Structures were illustrated using ChimeraX (Goddard et al. 2018) and Pymol (Schrödinger and DeLano 2020).

## Supplementary material

Supplementary data are available at *Molecular Biology and Evolution* online.

## Supplementary Material

msad209_Supplementary_Data

## Data Availability

All genomic data used in this analysis are available through GISAID. Accession identifiers are provided in supplementary data, Supplementary Material online. All other data are available upon request.
