# Supplementary material for: A Proofreading Mutation with an Allosteric Effect Allows a Cluster of SARS-CoV-2 Viruses to Rapidly Evolve
Source: Mol Biol Evol. 2023 Sep 20;40(10):msad209. doi: 10.1093/molbev/msad209 (PMC10553922; doi:10.1093/molbev/msad209)
Supplement: msad209_Supplementary_Data [file msad209_supplementary_data.zip › Supplementary Data - GISAID names.docx]

GISAID accessions

NSP14 mutant accessions

I201M

hCoV-19/England/DHSC-CYYSO11/2021|EPI_ISL_10469977|2021-12-27

hCoV-19/England/ALDP-2948E9A/2021|EPI_ISL_6148470|2021-11-04

hCoV-19/England/ALDP-29E473B/2021|EPI_ISL_6356857|2021-11-08

hCoV-19/England/ALDP-29E49CC/2021|EPI_ISL_6356950|2021-11-08

hCoV-19/England/ALDP-2A63171/2021|EPI_ISL_6551585|2021-11-13

hCoV-19/England/ALDP-2A63180/2021|EPI_ISL_6551720|2021-11-13

hCoV-19/England/QEUH-2AD4111/2021|EPI_ISL_6673233|2021-11-15

hCoV-19/England/QEUH-2B0AFF9/2021|EPI_ISL_6758032|2021-11-17

hCoV-19/England/QEUH-2B0B12F/2021|EPI_ISL_6758106|2021-11-16

hCoV-19/England/QEUH-2B0ACF2/2021|EPI_ISL_6758271|2021-11-16

hCoV-19/England/QEUH-2B0B031/2021|EPI_ISL_6758295|2021-11-16

hCoV-19/England/ALDP-2AF84C3/2021|EPI_ISL_6759538|2021-11-18

hCoV-19/England/ALDP-2B2D8D7/2021|EPI_ISL_6761707|2021-11-20

hCoV-19/England/ALDP-2B10682/2021|EPI_ISL_6763972|2021-11-18

hCoV-19/England/ALDP-2B2803E/2021|EPI_ISL_6799641|2021-11-19

hCoV-19/England/ALDP-2AFEE29/2021|EPI_ISL_6807820|2021-11-18

hCoV-19/England/ALDP-2B5E925/2021|EPI_ISL_6818986|2021-11-20

hCoV-19/England/ALDP-2B88403/2021|EPI_ISL_6836071|2021-11-21

hCoV-19/England/ALDP-2B88069/2021|EPI_ISL_6836135|2021-11-21

hCoV-19/England/ALDP-2AFFFDA/2021|EPI_ISL_6838969|2021-11-18

hCoV-19/England/ALDP-2B88AC5/2021|EPI_ISL_6840250|2021-11-21

hCoV-19/England/QEUH-2BB4CCA/2021|EPI_ISL_6867626|2021-11-22

hCoV-19/England/QEUH-2BA78F3/2021|EPI_ISL_6869370|2021-11-22

hCoV-19/England/QEUH-2BA974F/2021|EPI_ISL_6870465|2021-11-22

hCoV-19/England/ALDP-2B9FCF2/2021|EPI_ISL_6871333|2021-11-22

hCoV-19/England/ALDP-2BF22EC/2021|EPI_ISL_6915604|2021-11-25

hCoV-19/England/ALDP-2BEF18B/2021|EPI_ISL_6919804|2021-11-25

hCoV-19/England/ALDP-2C09A43/2021|EPI_ISL_7022297|2021-11-26

hCoV-19/England/QEUH-2C0BC4B/2021|EPI_ISL_7025706|2021-11-24

hCoV-19/England/ALDP-2C06D10/2021|EPI_ISL_7026417|2021-11-26

hCoV-19/England/QEUH-2C0EF30/2021|EPI_ISL_7030444|2021-11-24

hCoV-19/England/QEUH-2C0FFC6/2021|EPI_ISL_7030479|2021-11-25

hCoV-19/England/ALDP-2C3695F/2021|EPI_ISL_7144190|2021-11-27

hCoV-19/England/PHWC-PFTWPO/2021|EPI_ISL_7169536|2021-11-16

hCoV-19/England/ALDP-2C47320/2021|EPI_ISL_7187273|2021-11-28

hCoV-19/England/ALDP-2C473D5/2021|EPI_ISL_7187446|2021-11-28

hCoV-19/England/ALDP-2C51B85/2021|EPI_ISL_7187684|2021-11-27

hCoV-19/England/ALDP-2C87B2C/2021|EPI_ISL_7198168|2021-11-29

hCoV-19/England/ALDP-2C876FE/2021|EPI_ISL_7198319|2021-11-29

hCoV-19/England/ALDP-2CA42CA/2021|EPI_ISL_7296396|2021-11-30

hCoV-19/England/ALDP-2CA4066/2021|EPI_ISL_7296553|2021-11-30

hCoV-19/England/ALDP-2C9E14E/2021|EPI_ISL_7301729|2021-11-29

hCoV-19/England/ALDP-2CB7015/2021|EPI_ISL_7345752|2021-11-30

hCoV-19/England/ALDP-2CB22F6/2021|EPI_ISL_7346333|2021-11-30

hCoV-19/England/ALDP-2CB2162/2021|EPI_ISL_7346544|2021-11-30

hCoV-19/England/ALDP-2CA79A4/2021|EPI_ISL_7348745|2021-11-29

hCoV-19/England/ALDP-2CB3468/2021|EPI_ISL_7352569|2021-11-30

hCoV-19/England/ALDP-2D09981/2021|EPI_ISL_7382018|2021-12-02

hCoV-19/England/ALDP-2D09B30/2021|EPI_ISL_7382033|2021-12-02

hCoV-19/England/ALDP-2CF4F3D/2021|EPI_ISL_7389509|2021-12-02

hCoV-19/England/QEUH-2CE312C/2021|EPI_ISL_7394738|2021-11-30

hCoV-19/England/ALDP-2CA5EE3/2021|EPI_ISL_7397937|2021-11-29

hCoV-19/England/ALDP-2CEB6DA/2021|EPI_ISL_7482475|2021-12-01

hCoV-19/England/ALDP-2CEB94A/2021|EPI_ISL_7482507|2021-12-01

hCoV-19/England/ALDP-2D203A4/2021|EPI_ISL_7516344|2021-11-28

hCoV-19/England/ALDP-2D1D5FF/2021|EPI_ISL_7531363|2021-12-03

hCoV-19/England/ALDP-2D2190B/2021|EPI_ISL_7536625|2021-12-03

hCoV-19/England/ALDP-2D23E64/2021|EPI_ISL_7537291|2021-12-03

hCoV-19/England/ALDP-2D23DD0/2021|EPI_ISL_7537368|2021-12-03

hCoV-19/England/ALDP-2D23D2B/2021|EPI_ISL_7537486|2021-12-03

hCoV-19/England/ALDP-2D6BEE8/2021|EPI_ISL_7578167|2021-12-06

hCoV-19/England/ALDP-2D6C03C/2021|EPI_ISL_7578341|2021-12-06

hCoV-19/England/ALDP-2DA83B3/2021|EPI_ISL_7625002|2021-12-07

hCoV-19/England/ALDP-2DA3682/2021|EPI_ISL_7625569|2021-12-07

hCoV-19/England/ALDP-2DA3716/2021|EPI_ISL_7625614|2021-12-07

hCoV-19/England/QEUH-2DA1E31/2021|EPI_ISL_7626006|2021-12-03

hCoV-19/England/QEUH-2DA4DE6/2021|EPI_ISL_7628716|2021-12-05

hCoV-19/England/ALDP-2DA69C2/2021|EPI_ISL_7629637|2021-12-06

hCoV-19/England/ALDP-2D70211/2021|EPI_ISL_7631309|2021-12-06

hCoV-19/England/ALDP-2D70220/2021|EPI_ISL_7631415|2021-12-06

hCoV-19/England/ALDP-2D6FCDB/2021|EPI_ISL_7631811|2021-12-06

hCoV-19/England/ALDP-2D8A0CF/2021|EPI_ISL_7635065|2021-12-06

hCoV-19/England/ALDP-2D87824/2021|EPI_ISL_7635349|2021-12-06

hCoV-19/England/ALDP-2DCEDD7/2021|EPI_ISL_7676473|2021-12-08

hCoV-19/England/ALDP-2DCECBC/2021|EPI_ISL_7676550|2021-12-08

hCoV-19/England/ALDP-2DD57B1/2021|EPI_ISL_7677440|2021-12-08

hCoV-19/England/ALDP-2DBCB87/2021|EPI_ISL_7678600|2021-12-06

hCoV-19/England/QEUH-2DC9A7B/2021|EPI_ISL_7678857|2021-12-04

hCoV-19/England/ALDP-2DCAB2C/2021|EPI_ISL_7678882|2021-12-06

hCoV-19/England/QEUH-2DCB684/2021|EPI_ISL_7679029|2021-12-05

hCoV-19/England/QEUH-2DC96C2/2021|EPI_ISL_7679104|2021-12-06

hCoV-19/England/QEUH-2DCB5E1/2021|EPI_ISL_7679194|2021-12-05

hCoV-19/England/QEUH-2DAD8A0/2021|EPI_ISL_7682980|2021-12-06

hCoV-19/England/ALDP-2DD2004/2021|EPI_ISL_7724525|2021-12-07

hCoV-19/England/QEUH-2DE3EF7/2021|EPI_ISL_7725761|2021-12-07

hCoV-19/England/ALDP-2E0684A/2021|EPI_ISL_7760301|2021-12-08

hCoV-19/England/ALDP-2E01E3E/2021|EPI_ISL_7760388|2021-12-09

hCoV-19/England/ALDP-2E068E0/2021|EPI_ISL_7760611|2021-12-08

hCoV-19/England/ALDP-2E1673B/2021|EPI_ISL_7761682|2021-12-09

hCoV-19/England/ALDP-2E2DC9C/2021|EPI_ISL_7816372|2021-12-10

hCoV-19/England/ALDP-2E2DE1E/2021|EPI_ISL_7816410|2021-12-10

hCoV-19/England/ALDP-2E2CC9D/2021|EPI_ISL_7819688|2021-12-10

hCoV-19/England/ALDP-2E2A831/2021|EPI_ISL_7837046|2021-12-10

hCoV-19/England/QEUH-2E4BE65/2021|EPI_ISL_7842489|2021-12-09

hCoV-19/England/ALDP-2EA84A0/2021|EPI_ISL_7958210|2021-12-14

hCoV-19/England/ALDP-2E90511/2021|EPI_ISL_7960888|2021-12-11

hCoV-19/England/ALDP-2EB5BBA/2021|EPI_ISL_7963127|2021-12-12

hCoV-19/England/QEUH-2EB6A9E/2021|EPI_ISL_7972734|2021-12-14

hCoV-19/England/ALDP-2E9818E/2021|EPI_ISL_7973392|2021-12-13

hCoV-19/England/ALDP-2EADC67/2021|EPI_ISL_7974101|2021-12-12

hCoV-19/England/QEUH-2EF6CF6/2021|EPI_ISL_7990026|2021-12-14

hCoV-19/England/ALDP-2DCF0FB/2021|EPI_ISL_8051220|2021-12-07

hCoV-19/England/ALDP-2DCFE6A/2021|EPI_ISL_8051254|2021-12-08

hCoV-19/England/ALDP-2DCFC24/2021|EPI_ISL_8051268|2021-12-08

hCoV-19/England/ALDP-2DCFDF4/2021|EPI_ISL_8051307|2021-12-08

hCoV-19/England/QEUH-2F1AD25/2021|EPI_ISL_8071540|2021-12-14

hCoV-19/England/ALDP-2F23B89/2021|EPI_ISL_8072174|2021-12-17

hCoV-19/England/LSPA-2F5F5E0/2021|EPI_ISL_8084732|2021-12-15

hCoV-19/England/NORT-YBYR5R/2021|EPI_ISL_8133630|2021-12-13

hCoV-19/England/ALDP-2FBB88F/2021|EPI_ISL_8239805|2021-12-21

hCoV-19/England/ALDP-31BF72F/2022|EPI_ISL_8760386|2022-01-08

hCoV-19/England/PHEC-5M0B2ZA2/2021|EPI_ISL_9688418|2021-12-23

T25L

hCoV-19/Sichuan/SC-MY-058/2020|EPI_ISL_451324|2020-02-02

hCoV-19/Sichuan/SC-NC-076/2020|EPI_ISL_451335|2020-01-29

hCoV-19/Sichuan/SC-NJ-094/2020|EPI_ISL_451343|2020-01-30

hCoV-19/Sichuan/SC-PHCC1-028/2020|EPI_ISL_451349|2020-02-08

hCoV-19/Sichuan/SC-PHCC2-042/2020|EPI_ISL_451362|2020-02-13

hCoV-19/Sichuan/SC-PHCC2-046/2020|EPI_ISL_451366|2020-01-29

hCoV-19/Sichuan/SC-PHCC2-047/2020|EPI_ISL_451367|2020-02-08

hCoV-19/Sichuan/SC-PHCC2-052/2020|EPI_ISL_451372|2020-02-03

hCoV-19/Sichuan/SC-WCH-081/2020|EPI_ISL_451373|2020-02-03

hCoV-19/Sichuan/SC-WCH1-002/2020|EPI_ISL_451375|2020-02-08

hCoV-19/Sichuan/SC-YA-087/2020|EPI_ISL_451396|2020-01-30

hCoV-19/Sichuan/SC-YA-088/2020|EPI_ISL_451397|2020-01-31

hCoV-19/Wuhan/HB-WHCM-101/2020|EPI_ISL_455399|2020-03-02

hCoV-19/Wuhan/HB-WHCM-103/2020|EPI_ISL_455400|2020-03-02

hCoV-19/Wuhan/HB-WHCM-105/2020|EPI_ISL_455401|2020-03-02

hCoV-19/Wuhan/HB-WHCM-106/2020|EPI_ISL_455402|2020-03-02

hCoV-19/Wuhan/HB-WHCM-110/2020|EPI_ISL_455403|2020-03-02

hCoV-19/Wuhan/HB-WHCM-111/2020|EPI_ISL_455404|2020-03-02

hCoV-19/Wuhan/HB-WHCM-112/2020|EPI_ISL_455405|2020-03-02

hCoV-19/Wuhan/HB-WHCM-119/2020|EPI_ISL_455407|2020-03-02

hCoV-19/Wuhan/HB-WHCM-121/2020|EPI_ISL_455408|2020-03-02

hCoV-19/Wuhan/HB-WHCM2-273/2020|EPI_ISL_455409|2020-03-05

hCoV-19/Wuhan/HB-WHCM2-275/2020|EPI_ISL_455410|2020-03-03

hCoV-19/Wuhan/HB-WHCM2-277/2020|EPI_ISL_455411|2020-03-02

B.1.1. Control

hCoV-19/Wales/PHW04/2020|EPI_ISL_415041|2020-03-07

hCoV-19/Wales/PHWC-246B9/2020|EPI_ISL_419440|2020-03-17

hCoV-19/Wales/PHWC-26D49/2020|EPI_ISL_422022|2020-03-30

hCoV-19/Wales/PHWC-26A15/2020|EPI_ISL_422036|2020-03-29

hCoV-19/Wales/PHWC-26FCB/2020|EPI_ISL_422123|2020-03-30

hCoV-19/Wales/PHWC-259A0/2020|EPI_ISL_422138|2020-03-24

hCoV-19/Wales/PHWC-25F62/2020|EPI_ISL_422168|2020-03-25

hCoV-19/Wales/PHWC-265BA/2020|EPI_ISL_422192|2020-03-27

hCoV-19/Wales/PHWC-26F61/2020|EPI_ISL_422214|2020-03-28

hCoV-19/Wales/PHWC-252F0/2020|EPI_ISL_432177|2020-03-22

hCoV-19/Wales/PHWC-2A12B/2020|EPI_ISL_432178|2020-04-04

hCoV-19/Wales/PHWC-27AAB/2020|EPI_ISL_432227|2020-03-30

hCoV-19/Wales/PHWC-2B1A2/2020|EPI_ISL_432261|2020-04-07

hCoV-19/Wales/PHWC-2B01E/2020|EPI_ISL_432265|2020-04-08

hCoV-19/Wales/PHWC-27285/2020|EPI_ISL_432277|2020-03-31

hCoV-19/Wales/PHWC-29C58/2020|EPI_ISL_432292|2020-04-05

hCoV-19/Wales/PHWC-29FF5/2020|EPI_ISL_432344|2020-04-03

hCoV-19/Wales/PHWC-28891/2020|EPI_ISL_432349|2020-03-31

hCoV-19/Wales/PHWC-2A246/2020|EPI_ISL_445541|2020-04-05

hCoV-19/Wales/PHWC-2B412/2020|EPI_ISL_445708|2020-04-11

hCoV-19/Wales/PHWC-2BDAE/2020|EPI_ISL_445846|2020-04-10

hCoV-19/Wales/PHWC-2E1BE/2020|EPI_ISL_446068|2020-04-07

hCoV-19/Wales/PHWC-2EFD2/2020|EPI_ISL_446184|2020-04-04

hCoV-19/Wales/PHWC-2F0ED/2020|EPI_ISL_446200|2020-04-06

hCoV-19/Wales/PHWC-30AEB/2020|EPI_ISL_446320|2020-04-10

hCoV-19/Wales/PHWC-31B7E/2020|EPI_ISL_446535|2020-04-11

hCoV-19/Wales/PHWC-32375/2020|EPI_ISL_446638|2020-04-08

hCoV-19/Wales/PHWC-33B9A/2020|EPI_ISL_446796|2020-04-20

hCoV-19/Wales/PHWC-15C8DF/2020|EPI_ISL_472553|2020-04-18

hCoV-19/Wales/PHWC-15CC88/2020|EPI_ISL_472602|2020-05-07

hCoV-19/Wales/PHWC-15CE37/2020|EPI_ISL_472626|2020-04-20

hCoV-19/Wales/PHWC-15D285/2020|EPI_ISL_472692|2020-05-06

hCoV-19/Wales/PHWC-15D2FE/2020|EPI_ISL_472699|2020-05-07

hCoV-19/Wales/PHWC-15D8FC/2020|EPI_ISL_472750|2020-05-14

hCoV-19/Wales/PHWC-15DD93/2020|EPI_ISL_472775|2020-05-14

hCoV-19/Wales/PHWC-15E06C/2020|EPI_ISL_472790|2020-05-12

hCoV-19/Wales/PHWC-16002B/2020|EPI_ISL_472884|2020-04-18

hCoV-19/Wales/PHWC-1600D0/2020|EPI_ISL_472895|2020-05-15

hCoV-19/Wales/PHWC-160629/2020|EPI_ISL_472955|2020-04-19

hCoV-19/Wales/PHWC-160683/2020|EPI_ISL_472961|2020-04-09

hCoV-19/Wales/PHWC-160780/2020|EPI_ISL_472975|2020-05-23

hCoV-19/Wales/PHWC-160C81/2020|EPI_ISL_473034|2020-04-19

hCoV-19/Wales/PHWC-162539/2020|EPI_ISL_473085|2020-05-19

hCoV-19/Wales/PHWC-1627AC/2020|EPI_ISL_473119|2020-06-04

hCoV-19/Wales/PHWC-162988/2020|EPI_ISL_473146|2020-05-28

hCoV-19/Wales/PHWC-1636CC/2020|EPI_ISL_473981|2020-04-29

hCoV-19/Wales/PHWC-1638B7/2020|EPI_ISL_474011|2020-04-27

hCoV-19/Wales/PHWC-1642E5/2020|EPI_ISL_474157|2020-05-08

hCoV-19/Wales/PHWC-16431F/2020|EPI_ISL_474160|2020-05-06

hCoV-19/Wales/PHWC-164485/2020|EPI_ISL_474181|2020-05-02

hCoV-19/Wales/PHWC-164555/2020|EPI_ISL_474193|2020-05-06

hCoV-19/Wales/PHWC-164652/2020|EPI_ISL_474209|2020-05-10

hCoV-19/Wales/PHWC-29EF8/2020|EPI_ISL_474254|2020-04-02

hCoV-19/Wales/PHWC-2DEC7/2020|EPI_ISL_474318|2020-04-08

hCoV-19/Wales/PHWC-32700/2020|EPI_ISL_474343|2020-04-15

hCoV-19/Wales/PHWC-32B04/2020|EPI_ISL_474397|2020-04-15

hCoV-19/Wales/PHWC-357FE/2020|EPI_ISL_474479|2020-04-25

hCoV-19/Wales/PHWC-3612C/2020|EPI_ISL_474624|2020-04-28

hCoV-19/Wales/PHWC-36C85/2020|EPI_ISL_474785|2020-05-01

hCoV-19/Wales/PHWC-164C7E/2020|EPI_ISL_479365|2020-06-11

hCoV-19/Wales/PHWC-165615/2020|EPI_ISL_484689|2020-06-28

hCoV-19/Wales/PHWC-165633/2020|EPI_ISL_484691|2020-06-27

hCoV-19/Wales/PHWC-1657F4/2020|EPI_ISL_490727|2020-06-07

hCoV-19/Wales/PHWC-165ACE/2020|EPI_ISL_490763|2020-06-27

hCoV-19/Wales/PHWC-165C40/2020|EPI_ISL_490783|2020-06-16

hCoV-19/Wales/PHWC-165F56/2020|EPI_ISL_490818|2020-06-13

hCoV-19/Wales/PHWC-1663A4/2020|EPI_ISL_493990|2020-06-27

hCoV-19/Wales/PHWC-166429/2020|EPI_ISL_493997|2020-06-13

hCoV-19/Wales/PHWC-166474/2020|EPI_ISL_494001|2020-06-27

hCoV-19/Wales/PHWC-1664CF/2020|EPI_ISL_494005|2020-06-23

hCoV-19/Wales/PHWC-166562/2020|EPI_ISL_494015|2020-06-12

hCoV-19/Wales/PHWC-1665CC/2020|EPI_ISL_494020|2020-06-27

hCoV-19/Wales/PHWC-16666F/2020|EPI_ISL_494026|2020-06-27

hCoV-19/Wales/PHWC-1668F0/2020|EPI_ISL_494058|2020-06-27

hCoV-19/Wales/PHWC-166AEB/2020|EPI_ISL_494080|2020-06-27

hCoV-19/Wales/PHWC-167006/2020|EPI_ISL_494140|2020-06-23

hCoV-19/Wales/PHWC-1681D5/2020|EPI_ISL_494211|2020-05-28

hCoV-19/Wales/PHWC-16831B/2020|EPI_ISL_494226|2020-04-20

hCoV-19/Wales/PHWC-168D95/2020|EPI_ISL_494364|2020-04-01

hCoV-19/Wales/PHWC-168F44/2020|EPI_ISL_499370|2020-04-05

hCoV-19/Wales/PHWC-1694AE/2020|EPI_ISL_499447|2020-04-01

hCoV-19/Wales/PHWC-1694F9/2020|EPI_ISL_499451|2020-04-01

hCoV-19/Wales/QEUH-961E55/2020|EPI_ISL_537093|2020-08-01

hCoV-19/Wales/ALDP-954383/2020|EPI_ISL_553819|2020-06-12

hCoV-19/Wales/ALDP-9543A1/2020|EPI_ISL_553844|2020-06-12

hCoV-19/Wales/MILK-958729/2020|EPI_ISL_554783|2020-06-10

hCoV-19/Wales/ALDP-952CA8/2020|EPI_ISL_555351|2020-06-12

hCoV-19/Wales/ALDP-94E568/2020|EPI_ISL_555392|2020-06-16

hCoV-19/Wales/ALDP-952C5D/2020|EPI_ISL_555406|2020-06-11

hCoV-19/Wales/ALDP-952C8A/2020|EPI_ISL_555460|2020-06-12

hCoV-19/Wales/ALDP-94F3A9/2020|EPI_ISL_555873|2020-06-19

hCoV-19/Wales/ALDP-6DA8D4/2020|EPI_ISL_556130|2020-06-30

hCoV-19/Wales/MILK-95DA49/2020|EPI_ISL_556235|2020-06-19

hCoV-19/Wales/ALDP-5A05A4/2020|EPI_ISL_558641|2020-06-12

hCoV-19/Wales/ALDP-5A8AE9/2020|EPI_ISL_558741|2020-06-13

hCoV-19/Wales/MILK-9EF4E4/2020|EPI_ISL_594922|2020-09-25

hCoV-19/Wales/ALDP-944140/2020|EPI_ISL_602159|2020-06-07

hCoV-19/Wales/PHWC-4871FB/2020|EPI_ISL_639173|2020-10-31

hCoV-19/Wales/PHWC-487243/2020|EPI_ISL_639176|2020-10-31

hCoV-19/Wales/QEUH-CC31BF/2020|EPI_ISL_892214|2020-12-21

B.1.1.41 Control

hCoV-19/England/PHEC-YYDRQZA/2020|EPI_ISL_11156026|2020-05-22

hCoV-19/England/SHEF-BFF4F/2020|EPI_ISL_418311|2020-03-25

hCoV-19/England/NOTT-10E5C6/2020|EPI_ISL_425567|2020-03-23

hCoV-19/England/NOTT-10E5D5/2020|EPI_ISL_425568|2020-03-22

hCoV-19/England/NOTT-10E951/2020|EPI_ISL_425616|2020-03-30

hCoV-19/England/NOTT-10EAF4/2020|EPI_ISL_425640|2020-03-30

hCoV-19/England/CAMB-77927/2020|EPI_ISL_439470|2020-03-31

hCoV-19/England/CAMB-73141/2020|EPI_ISL_440297|2020-03-21

hCoV-19/England/NOTT-1110DE/2020|EPI_ISL_453628|2020-05-03

hCoV-19/England/NOTT-11141E/2020|EPI_ISL_453662|2020-05-17

hCoV-19/England/NORW-EAD32/2020|EPI_ISL_457371|2020-04-28

hCoV-19/England/NOTT-1115DF/2020|EPI_ISL_461896|2020-05-18

hCoV-19/England/NOTT-11188B/2020|EPI_ISL_461934|2020-05-22

hCoV-19/England/NOTT-11189A/2020|EPI_ISL_461935|2020-05-22

hCoV-19/England/NOTT-111A3A/2020|EPI_ISL_461953|2020-05-28

hCoV-19/England/NOTT-111CBC/2020|EPI_ISL_472394|2020-06-05

hCoV-19/England/NOTT-111F59/2020|EPI_ISL_472428|2020-05-02

hCoV-19/England/NOTT-111F68/2020|EPI_ISL_472429|2020-05-03

hCoV-19/England/BIRM-5F891/2020|EPI_ISL_473344|2020-05-16

hCoV-19/England/LCST-24D687A/2020|EPI_ISL_478304|2020-05-31

hCoV-19/England/LCST-24D68F2/2020|EPI_ISL_478311|2020-06-01

hCoV-19/England/LCST-24D70BD/2020|EPI_ISL_478362|2020-06-07

hCoV-19/England/NOTT-11227D/2020|EPI_ISL_484361|2020-05-01

hCoV-19/England/NOTT-11228C/2020|EPI_ISL_484362|2020-05-01

hCoV-19/England/NOTT-1123F2/2020|EPI_ISL_484378|2020-05-30

hCoV-19/England/NOTT-1125CF/2020|EPI_ISL_484400|2020-04-29

hCoV-19/England/NOTT-1125DE/2020|EPI_ISL_484401|2020-04-24

hCoV-19/England/NOTT-1125ED/2020|EPI_ISL_484402|2020-04-29

hCoV-19/England/NOTT-112969/2020|EPI_ISL_493567|2020-04-20

hCoV-19/England/LIVE-A7F3B/2020|EPI_ISL_517093|2020-03-26

hCoV-19/England/LEED-2A9702/2020|EPI_ISL_538776|2020-04-08

hCoV-19/England/LEED-2A95AE/2020|EPI_ISL_538817|2020-04-09

hCoV-19/England/LEED-2A9A63/2020|EPI_ISL_538837|2020-04-14

hCoV-19/England/LEED-2A9508/2020|EPI_ISL_538888|2020-04-09

hCoV-19/England/LEED-2A98E1/2020|EPI_ISL_538916|2020-04-28

hCoV-19/England/LEED-2A94A1/2020|EPI_ISL_538930|2020-04-13

hCoV-19/England/LEED-2A8202/2020|EPI_ISL_538999|2020-04-27

hCoV-19/England/LEED-2A81E7/2020|EPI_ISL_539029|2020-04-26

hCoV-19/England/LEED-2A8D7A/2020|EPI_ISL_539033|2020-03-24

hCoV-19/England/LEED-2A9131/2020|EPI_ISL_539079|2020-04-07

hCoV-19/England/LEED-2A8ED1/2020|EPI_ISL_539103|2020-04-06

hCoV-19/England/LEED-2A8FDE/2020|EPI_ISL_539108|2020-04-05

hCoV-19/England/LEED-2A8B25/2020|EPI_ISL_539111|2020-04-01

hCoV-19/England/LEED-2A8123/2020|EPI_ISL_539122|2020-04-14

hCoV-19/England/LEED-2A823F/2020|EPI_ISL_539138|2020-04-06

hCoV-19/England/LEED-2A8E68/2020|EPI_ISL_539163|2020-03-31

hCoV-19/England/ALDP-90604D/2020|EPI_ISL_551382|2020-06-24

hCoV-19/England/ALDP-938BF6/2020|EPI_ISL_551423|2020-06-21

hCoV-19/England/ALDP-906186/2020|EPI_ISL_551445|2020-06-24

hCoV-19/England/ALDP-9060D4/2020|EPI_ISL_551510|2020-06-24

hCoV-19/England/MILK-6AB0B6/2020|EPI_ISL_551753|2020-07-01

hCoV-19/England/ALDP-95218B/2020|EPI_ISL_553642|2020-06-09

hCoV-19/England/ALDP-95222E/2020|EPI_ISL_553643|2020-06-09

hCoV-19/England/ALDP-95221F/2020|EPI_ISL_553646|2020-06-09

hCoV-19/England/MILK-95799D/2020|EPI_ISL_554185|2020-06-09

hCoV-19/England/ALDP-94D7DC/2020|EPI_ISL_554950|2020-06-14

hCoV-19/England/ALDP-950299/2020|EPI_ISL_555152|2020-06-17

hCoV-19/England/ALDP-94FE5D/2020|EPI_ISL_555212|2020-06-16

hCoV-19/England/ALDP-95028A/2020|EPI_ISL_555273|2020-06-17

hCoV-19/England/ALDP-94A344/2020|EPI_ISL_556493|2020-06-11

hCoV-19/England/ALDP-528A87/2020|EPI_ISL_558989|2020-06-01

hCoV-19/England/ALDP-519CED/2020|EPI_ISL_559076|2020-06-02

hCoV-19/England/ALDP-497F2B/2020|EPI_ISL_559550|2020-06-03

hCoV-19/England/ALDP-4A35C0/2020|EPI_ISL_559573|2020-05-27

hCoV-19/England/ALDP-497D12/2020|EPI_ISL_559647|2020-06-03

hCoV-19/England/ALDP-9ECECB/2020|EPI_ISL_589271|2020-05-04

hCoV-19/England/ALDP-9EDDBE/2020|EPI_ISL_589284|2020-05-06

hCoV-19/England/ALDP-9EE3CA/2020|EPI_ISL_589306|2020-05-06

hCoV-19/England/ALDP-9ED510/2020|EPI_ISL_589350|2020-05-05

hCoV-19/England/ALDP-9EE38E/2020|EPI_ISL_589358|2020-05-06

hCoV-19/England/ALDP-9ED598/2020|EPI_ISL_589369|2020-05-08

hCoV-19/England/ALDP-9ED2DD/2020|EPI_ISL_589371|2020-05-04

hCoV-19/England/ALDP-9ECE34/2020|EPI_ISL_589439|2020-05-05

hCoV-19/England/ALDP-9ED325/2020|EPI_ISL_589487|2020-05-04

hCoV-19/England/ALDP-9ED316/2020|EPI_ISL_589507|2020-05-04

hCoV-19/England/ALDP-9EE3D9/2020|EPI_ISL_589563|2020-05-06

hCoV-19/England/NOTT-11F3C8/2020|EPI_ISL_679967|2020-05-12

C39F

hCoV-19/England/QEUH-131EBCC/2021|EPI_ISL_1175193|2021-02-24

hCoV-19/England/QEUH-13642E0/2021|EPI_ISL_1187264|2021-02-27

hCoV-19/England/QEUH-136423B/2021|EPI_ISL_1187280|2021-02-27

hCoV-19/England/QEUH-13641E3/2021|EPI_ISL_1187427|2021-02-27

hCoV-19/England/QEUH-13617A8/2021|EPI_ISL_1187487|2021-02-28

hCoV-19/England/QEUH-13642A4/2021|EPI_ISL_1187501|2021-02-27

hCoV-19/England/QEUH-13353D6/2021|EPI_ISL_1187637|2021-02-25

hCoV-19/England/QEUH-13353B8/2021|EPI_ISL_1187684|2021-02-25

hCoV-19/England/QEUH-1335039/2021|EPI_ISL_1187707|2021-02-25

hCoV-19/England/QEUH-132C545/2021|EPI_ISL_1188457|2021-02-25

hCoV-19/England/QEUH-132C5BE/2021|EPI_ISL_1188537|2021-02-25

hCoV-19/England/QEUH-132C572/2021|EPI_ISL_1188593|2021-02-25

hCoV-19/England/QEUH-135DB58/2021|EPI_ISL_1205644|2021-02-28

hCoV-19/England/QEUH-135F691/2021|EPI_ISL_1223023|2021-02-28

hCoV-19/England/QEUH-1367757/2021|EPI_ISL_1223568|2021-03-01

hCoV-19/England/QEUH-1369254/2021|EPI_ISL_1224052|2021-03-01

hCoV-19/England/QEUH-13710D7/2021|EPI_ISL_1243756|2021-02-27

hCoV-19/England/QEUH-1351D7C/2021|EPI_ISL_1246019|2021-02-27

hCoV-19/England/QEUH-1351D8B/2021|EPI_ISL_1246201|2021-02-27

hCoV-19/England/NORT-1B8ACB1/2021|EPI_ISL_1248896|2021-03-01

hCoV-19/England/QEUH-138BFBF/2021|EPI_ISL_1250220|2021-03-04

hCoV-19/England/QEUH-139F352/2021|EPI_ISL_1256100|2021-03-05

hCoV-19/England/QEUH-139EEF8/2021|EPI_ISL_1256197|2021-03-06

hCoV-19/England/QEUH-1310465/2021|EPI_ISL_1257288|2021-02-22

hCoV-19/England/QEUH-13AD746/2021|EPI_ISL_1263619|2021-03-08

hCoV-19/England/QEUH-139E9CA/2021|EPI_ISL_1264075|2021-03-06

hCoV-19/England/QEUH-139E9E8/2021|EPI_ISL_1264179|2021-03-05

hCoV-19/England/QEUH-13A0B29/2021|EPI_ISL_1264240|2021-03-06

hCoV-19/England/QEUH-137BC24/2021|EPI_ISL_1264756|2021-03-03

hCoV-19/England/QEUH-13B3D87/2021|EPI_ISL_1274501|2021-03-08

hCoV-19/England/QEUH-13ACC66/2021|EPI_ISL_1276628|2021-03-08

hCoV-19/England/QEUH-13AA08A/2021|EPI_ISL_1276652|2021-03-08

hCoV-19/England/QEUH-138C30D/2021|EPI_ISL_1310666|2021-03-04

hCoV-19/England/QEUH-139F659/2021|EPI_ISL_1316441|2021-03-05

hCoV-19/England/QEUH-139F4E6/2021|EPI_ISL_1316730|2021-03-05

hCoV-19/England/MILK-13E4154/2021|EPI_ISL_1324912|2021-03-13

hCoV-19/England/QEUH-13E88E2/2021|EPI_ISL_1325785|2021-03-13

hCoV-19/England/QEUH-13D0935/2021|EPI_ISL_1326274|2021-03-12

hCoV-19/England/QEUH-13DA625/2021|EPI_ISL_1328182|2021-03-13

hCoV-19/England/QEUH-13C3CA5/2021|EPI_ISL_1328801|2021-03-09

hCoV-19/England/QEUH-13CF201/2021|EPI_ISL_1330220|2021-03-10

hCoV-19/England/QEUH-13C10E7/2021|EPI_ISL_1332333|2021-03-11

hCoV-19/England/QEUH-13C1F62/2021|EPI_ISL_1332531|2021-03-10

hCoV-19/England/MILK-140FD30/2021|EPI_ISL_1375371|2021-03-17

hCoV-19/England/MILK-140FBFA/2021|EPI_ISL_1375463|2021-03-17

hCoV-19/England/QEUH-141281B/2021|EPI_ISL_1376085|2021-03-10

hCoV-19/England/QEUH-1451494/2021|EPI_ISL_1452844|2021-03-22

hCoV-19/England/QEUH-1451564/2021|EPI_ISL_1452850|2021-03-22

hCoV-19/England/ALDP-148B929/2021|EPI_ISL_1487601|2021-03-26

hCoV-19/England/ALDP-148ADA6/2021|EPI_ISL_1487618|2021-03-25

hCoV-19/England/ALDP-148AB51/2021|EPI_ISL_1487686|2021-03-25

hCoV-19/England/ALDP-14A8B02/2021|EPI_ISL_1519466|2021-03-29

hCoV-19/England/ALDP-14A8AF6/2021|EPI_ISL_1519509|2021-03-29

hCoV-19/England/ALDP-14A8B7B/2021|EPI_ISL_1519585|2021-03-29

F60S

hCoV-19/Mexico/NLE-LESPNL-00970/2021|EPI_ISL_7716412|2021-03-17

hCoV-19/Mexico/NLE-LESPNL-00971/2021|EPI_ISL_7716413|2021-03-26

hCoV-19/Mexico/NLE-LESPNL-00972/2021|EPI_ISL_7716414|2021-03-29

hCoV-19/Mexico/NLE-LESPNL-00973/2021|EPI_ISL_7716415|2021-03-30

hCoV-19/Mexico/NLE-LESPNL-00975/2021|EPI_ISL_7716416|2021-03-26

hCoV-19/Mexico/NLE-LESPNL-00976/2021|EPI_ISL_7716417|2021-03-26

hCoV-19/Mexico/NLE-LESPNL-00977/2021|EPI_ISL_7716418|2021-03-27

hCoV-19/Mexico/NLE-LESPNL-00978/2021|EPI_ISL_7716419|2021-03-27

hCoV-19/Mexico/NLE-LESPNL-00979/2021|EPI_ISL_7716420|2021-03-27

hCoV-19/Mexico/NLE-LESPNL-00980/2021|EPI_ISL_7716421|2021-03-26

hCoV-19/Mexico/NLE-LESPNL-00981/2021|EPI_ISL_7716422|2021-04-02

hCoV-19/Mexico/NLE-LESPNL-00982/2021|EPI_ISL_7716423|2021-04-01

hCoV-19/Mexico/NLE-LESPNL-00983/2021|EPI_ISL_7716424|2021-04-02

hCoV-19/Mexico/NLE-LESPNL-00985/2021|EPI_ISL_7716426|2021-03-19

hCoV-19/Mexico/NLE-LESPNL-00986/2021|EPI_ISL_7716427|2021-03-20

hCoV-19/Mexico/NLE-LESPNL-00988/2021|EPI_ISL_7716428|2021-03-18

hCoV-19/Mexico/NLE-LESPNL-00989/2021|EPI_ISL_7716429|2021-03-19

hCoV-19/Mexico/NLE-LESPNL-00990/2021|EPI_ISL_7716430|2021-03-21

hCoV-19/Mexico/NLE-LESPNL-00991/2021|EPI_ISL_7716431|2021-03-23

hCoV-19/Mexico/NLE-LESPNL-00992/2021|EPI_ISL_7716432|2021-03-26

hCoV-19/Mexico/NLE-LESPNL-00993/2021|EPI_ISL_7716433|2021-03-26

hCoV-19/Mexico/NLE-LESPNL-00994/2021|EPI_ISL_7716434|2021-03-26

hCoV-19/Mexico/NLE-LESPNL-00996/2021|EPI_ISL_7716436|2021-04-02

hCoV-19/Mexico/NLE-LESPNL-00997/2021|EPI_ISL_7716438|2021-03-25

hCoV-19/Mexico/NLE-LESPNL-00998/2021|EPI_ISL_7716439|2021-03-23

hCoV-19/Mexico/NLE-LESPNL-01000/2021|EPI_ISL_7716440|2021-03-13

hCoV-19/Mexico/NLE-LESPNL-01002/2021|EPI_ISL_7716441|2021-03-13

hCoV-19/Mexico/NLE-LESPNL-01003/2021|EPI_ISL_7716442|2021-03-19

hCoV-19/Mexico/NLE-LESPNL-01004/2021|EPI_ISL_7716443|2021-03-19

hCoV-19/Mexico/NLE-LESPNL-01005/2021|EPI_ISL_7716444|2021-03-19

hCoV-19/Mexico/NLE-LESPNL-01006/2021|EPI_ISL_7716445|2021-04-01

hCoV-19/Mexico/NLE-LESPNL-01007/2021|EPI_ISL_7716446|2021-03-25

hCoV-19/Mexico/NLE-LESPNL-01008/2021|EPI_ISL_7716447|2021-04-03

hCoV-19/Mexico/NLE-LESPNL-01009/2021|EPI_ISL_7716448|2021-04-02

hCoV-19/Mexico/NLE-LESPNL-01010/2021|EPI_ISL_7716449|2021-03-30

hCoV-19/Mexico/NLE-LESPNL-01015/2021|EPI_ISL_7716450|2021-03-25

hCoV-19/Mexico/NLE-LESPNL-01016/2021|EPI_ISL_7716451|2021-04-03

hCoV-19/Mexico/NLE-LESPNL-01017/2021|EPI_ISL_7716452|2021-04-01

hCoV-19/Mexico/NLE-LESPNL-01018/2021|EPI_ISL_7716453|2021-03-19

hCoV-19/Mexico/NLE-LESPNL-01020/2021|EPI_ISL_7716455|2021-03-26

hCoV-19/Mexico/NLE-LESPNL-01021/2021|EPI_ISL_7716456|2021-03-27

hCoV-19/Mexico/NLE-LESPNL-01022/2021|EPI_ISL_7716457|2021-03-31

hCoV-19/Mexico/NLE-LESPNL-01023/2021|EPI_ISL_7716458|2021-04-01

hCoV-19/Mexico/NLE-LESPNL-01024/2021|EPI_ISL_7716459|2021-04-02

hCoV-19/Mexico/NLE-LESPNL-01025/2021|EPI_ISL_7716460|2021-04-02

hCoV-19/Mexico/NLE-LESPNL-01046/2021|EPI_ISL_7716481|2021-04-02

hCoV-19/Mexico/NLE-LESPNL-01047/2021|EPI_ISL_7716482|2021-03-14

hCoV-19/Mexico/NLE-LESPNL-01048/2021|EPI_ISL_7716483|2021-03-14

hCoV-19/Mexico/NLE-LESPNL-01049/2021|EPI_ISL_7716484|2021-03-20

hCoV-19/Mexico/NLE-LESPNL-01050/2021|EPI_ISL_7716485|2021-03-16

hCoV-19/Mexico/NLE-LESPNL-01051/2021|EPI_ISL_7716486|2021-03-17

hCoV-19/Mexico/NLE-LESPNL-01052/2021|EPI_ISL_7716487|2021-03-18

hCoV-19/Mexico/NLE-LESPNL-01053/2021|EPI_ISL_7716488|2021-03-18

hCoV-19/Mexico/NLE-LESPNL-01054/2021|EPI_ISL_7716489|2021-03-19

hCoV-19/Mexico/NLE-LESPNL-001055/2021|EPI_ISL_7730969|2021-03-26

H26N

hCoV-19/Wales/PHWC-PFPOIC/2021|EPI_ISL_7011877|2021-11-11

hCoV-19/Wales/PHWC-PFPOOS/2021|EPI_ISL_7011896|2021-11-11

hCoV-19/Wales/PHWC-PF16RS/2021|EPI_ISL_7349158|2021-11-19

hCoV-19/Wales/PHWC-PF31CR/2021|EPI_ISL_7727727|2021-11-29

hCoV-19/Wales/PHWC-PF3PFZ/2021|EPI_ISL_7728406|2021-11-29

hCoV-19/Wales/PHWC-PF3PR3/2021|EPI_ISL_7728419|2021-11-29

hCoV-19/Wales/PHWC-PF51NW/2021|EPI_ISL_7843890|2021-12-01

hCoV-19/Wales/PHWC-PF6PWQ/2021|EPI_ISL_7870598|2021-11-27

hCoV-19/Wales/PHWC-PF9FUS/2021|EPI_ISL_7874176|2021-11-30

hCoV-19/Wales/PHWC-PF9G97/2021|EPI_ISL_7874192|2021-11-30

hCoV-19/Wales/PHWC-PGY8Q7/2021|EPI_ISL_7997822|2021-12-06

hCoV-19/Wales/PHWC-PGY8U1/2021|EPI_ISL_7997886|2021-12-06

hCoV-19/Wales/PHWC-PGY8WO/2021|EPI_ISL_7997887|2021-12-06

hCoV-19/Wales/PHWC-PGYHF4/2021|EPI_ISL_7998610|2021-12-05

hCoV-19/Wales/PHWC-PGYNST/2021|EPI_ISL_7999505|2021-12-05

hCoV-19/Wales/PHWC-PGNNKG/2021|EPI_ISL_8118906|2021-12-02

hCoV-19/Wales/PHWC-PGNOA5/2021|EPI_ISL_8118924|2021-12-02

hCoV-19/Wales/PHWC-PGNOS9/2021|EPI_ISL_8118940|2021-12-02

hCoV-19/Wales/PHWC-PGRQ5U/2021|EPI_ISL_8141762|2021-12-09

hCoV-19/Wales/PHWC-PG8FOC/2021|EPI_ISL_8171276|2021-12-10

hCoV-19/Wales/PHWC-PGJPT6/2021|EPI_ISL_8288473|2021-12-13

hCoV-19/Wales/PHWC-PG1J6I/2021|EPI_ISL_8746037|2021-12-08

hCoV-19/Wales/PHWC-PG1KM5/2021|EPI_ISL_8746081|2021-12-08

hCoV-19/Wales/PHWC-PGIWQF/2021|EPI_ISL_8784434|2021-12-22

H26Y

hCoV-19/Wales/PHWC-PYFMAY/2021|EPI_ISL_2434822|2021-05-25

hCoV-19/Wales/PHWC-PYFMZN/2021|EPI_ISL_2434843|2021-05-25

hCoV-19/Wales/ALDP-1620186/2021|EPI_ISL_2516308|2021-06-01

hCoV-19/Wales/ALDP-168DE44/2021|EPI_ISL_2640078|2021-06-08

hCoV-19/Wales/PHWC-PY8F9W/2021|EPI_ISL_2707442|2021-06-18

hCoV-19/Wales/PHWC-PY8FJB/2021|EPI_ISL_2707451|2021-06-19

hCoV-19/Wales/PHWC-PYGT6C/2021|EPI_ISL_2708011|2021-06-15

hCoV-19/Wales/PHWC-PY8976/2021|EPI_ISL_2750000|2021-06-17

hCoV-19/Wales/PHWC-PY89HY/2021|EPI_ISL_2750009|2021-06-17

hCoV-19/Wales/PHWC-PYE31A/2021|EPI_ISL_2750089|2021-06-21

hCoV-19/Wales/PHWC-PYE3T4/2021|EPI_ISL_2750111|2021-06-21

hCoV-19/Wales/PHWC-PYE3X9/2021|EPI_ISL_2750114|2021-06-21

hCoV-19/Wales/PHWC-PYE4GO/2021|EPI_ISL_2750125|2021-06-21

hCoV-19/Wales/PHWC-PYEMTE/2021|EPI_ISL_2750387|2021-06-22

hCoV-19/Wales/PHWC-PYEMXP/2021|EPI_ISL_2750390|2021-06-22

hCoV-19/Wales/PHWC-PYEOYG/2021|EPI_ISL_2750434|2021-06-21

hCoV-19/Wales/PHWC-PYEP9K/2021|EPI_ISL_2750441|2021-06-22

hCoV-19/Wales/PHWC-PYEQYE/2021|EPI_ISL_2750486|2021-06-22

hCoV-19/Wales/PHWC-PYEZO6/2021|EPI_ISL_2750680|2021-06-21

hCoV-19/Wales/PHWC-PYE63F/2021|EPI_ISL_2818341|2021-06-26

hCoV-19/Wales/PHWC-PYE6A8/2021|EPI_ISL_2818346|2021-06-26

hCoV-19/Wales/PHWC-PYJ48C/2021|EPI_ISL_2818578|2021-06-25

hCoV-19/Wales/PHWC-PYJ4O3/2021|EPI_ISL_2818590|2021-06-24

hCoV-19/Wales/PHWC-PYJD8D/2021|EPI_ISL_2818809|2021-06-25

hCoV-19/Wales/PHWC-PYJDF8/2021|EPI_ISL_2818816|2021-06-25

hCoV-19/Wales/PHWC-PYJMCT/2021|EPI_ISL_2819028|2021-06-23

hCoV-19/Wales/PHWC-PYJPKU/2021|EPI_ISL_2819178|2021-06-23

hCoV-19/Wales/PHWC-PYJRCA/2021|EPI_ISL_2819273|2021-06-24

hCoV-19/Wales/PHWC-PYJRM4/2021|EPI_ISL_2819288|2021-06-24

hCoV-19/Wales/PHWC-PYJXU6/2021|EPI_ISL_2819572|2021-06-22

hCoV-19/Wales/PHWC-PYJXZS/2021|EPI_ISL_2819576|2021-06-23

hCoV-19/Wales/PHWC-PYCMA1/2021|EPI_ISL_2904890|2021-06-29

hCoV-19/Wales/PHWC-PYCMZW/2021|EPI_ISL_2904907|2021-06-29

hCoV-19/Wales/PHWC-PYK13P/2021|EPI_ISL_2905225|2021-06-28

hCoV-19/Wales/PHWC-PYK3CB/2021|EPI_ISL_2905260|2021-06-29

hCoV-19/Wales/PHWC-PYK4QH/2021|EPI_ISL_2905302|2021-06-29

hCoV-19/Wales/PHWC-PYK5WQ/2021|EPI_ISL_2905330|2021-06-29

hCoV-19/Wales/PHWC-PYKXGZ/2021|EPI_ISL_2905876|2021-06-28

hCoV-19/Wales/PHWC-PYMDHW/2021|EPI_ISL_2906214|2021-06-27

hCoV-19/Wales/ALDP-1888524/2021|EPI_ISL_2974600|2021-07-02

hCoV-19/Wales/PHWC-PYP9J5/2021|EPI_ISL_3004673|2021-07-06

hCoV-19/Wales/PHWC-PYPAFK/2021|EPI_ISL_3004698|2021-07-04

hCoV-19/Wales/PHWC-PYPENY/2021|EPI_ISL_3004783|2021-07-03

hCoV-19/Wales/PHWC-PYPTHN/2021|EPI_ISL_3005129|2021-07-05

hCoV-19/Wales/PHWC-PYPWYA/2021|EPI_ISL_3005186|2021-07-01

hCoV-19/Wales/QEUH-1927C7C/2021|EPI_ISL_3070752|2021-07-12

hCoV-19/Wales/PHWC-PY1GTA/2021|EPI_ISL_3081719|2021-07-07

hCoV-19/Wales/PHWC-PY1TM4/2021|EPI_ISL_3082007|2021-07-11

hCoV-19/Wales/PHWC-PYSBUT/2021|EPI_ISL_3181658|2021-07-16

hCoV-19/Wales/PHWC-PY44RX/2021|EPI_ISL_3294270|2021-07-27

hCoV-19/Wales/PHWC-PY4IZP/2021|EPI_ISL_3294571|2021-07-27

hCoV-19/Wales/PHWC-PYAXPC/2021|EPI_ISL_3295727|2021-07-19

hCoV-19/Wales/PHWC-PYAXQK/2021|EPI_ISL_3295729|2021-07-19

hCoV-19/Wales/PHWC-PYH4M7/2021|EPI_ISL_3295953|2021-07-28

hCoV-19/Wales/PHWC-PYHO1A/2021|EPI_ISL_3296620|2021-07-28

hCoV-19/Wales/PHWC-PDBY7G/2021|EPI_ISL_3422604|2021-08-09

hCoV-19/Wales/PHWC-PY6BDN/2021|EPI_ISL_3423435|2021-07-29

hCoV-19/Wales/PHWC-PY7PKK/2021|EPI_ISL_3424190|2021-07-31

hCoV-19/Wales/PHWC-PYHH54/2021|EPI_ISL_3425176|2021-08-01

hCoV-19/Wales/PHWC-PDBCNT/2021|EPI_ISL_3575058|2021-08-06

hCoV-19/Wales/PHWC-PDBXEN/2021|EPI_ISL_3575354|2021-08-07

hCoV-19/Wales/PHWC-PDNGTS/2021|EPI_ISL_3575804|2021-08-11

hCoV-19/Wales/PHWC-PDNRIO/2021|EPI_ISL_3576089|2021-08-10

hCoV-19/Wales/PHWC-PDNSWY/2021|EPI_ISL_3576133|2021-08-10

hCoV-19/Wales/PHWC-PDDYKJ/2021|EPI_ISL_3788912|2021-08-11

hCoV-19/Wales/PHWC-PDN63Q/2021|EPI_ISL_3790159|2021-08-12

hCoV-19/Wales/PHWC-PDNHKX/2021|EPI_ISL_3790478|2021-08-11

hCoV-19/Wales/PHWC-PDRUM1/2021|EPI_ISL_3792049|2021-08-15

hCoV-19/Wales/PHWC-PDRWBF/2021|EPI_ISL_3792067|2021-08-16

hCoV-19/Wales/PHWC-PDRWXJ/2021|EPI_ISL_3792086|2021-08-16

hCoV-19/Wales/PHWC-PD8HX5/2021|EPI_ISL_3962962|2021-08-22

hCoV-19/Wales/PHWC-PDGSOY/2021|EPI_ISL_3965261|2021-08-15

hCoV-19/Wales/PHWC-PDE1SW/2021|EPI_ISL_4133143|2021-08-19

hCoV-19/Wales/PHWC-PDJ81F/2021|EPI_ISL_4134048|2021-08-17

hCoV-19/Wales/PHWC-PDJ8UD/2021|EPI_ISL_4134068|2021-08-17

hCoV-19/Wales/PHWC-PDJ8WB/2021|EPI_ISL_4134069|2021-08-17

hCoV-19/Wales/PHWC-PDJG9C/2021|EPI_ISL_4134255|2021-08-17

hCoV-19/Wales/PHWC-PDK14E/2021|EPI_ISL_4134753|2021-08-26

hCoV-19/Wales/PHWC-PDKF3Z/2021|EPI_ISL_4135118|2021-08-23

hCoV-19/Wales/PHWC-PDPT3F/2021|EPI_ISL_4137025|2021-07-29

hCoV-19/Wales/PHWC-PD166D/2021|EPI_ISL_4312736|2021-08-30

hCoV-19/Wales/PHWC-PD1X5A/2021|EPI_ISL_4313535|2021-08-16

hCoV-19/Wales/PHWC-PDP7A5/2021|EPI_ISL_4313692|2021-09-01

hCoV-19/Wales/PHWC-PDQMF1/2021|EPI_ISL_4314390|2021-08-31

hCoV-19/Wales/PHWC-PDT54O/2021|EPI_ISL_4314817|2021-08-29

hCoV-19/Wales/PHWC-PDT5X8/2021|EPI_ISL_4314842|2021-08-29

hCoV-19/Wales/PHWC-PDTK75/2021|EPI_ISL_4315211|2021-09-02

hCoV-19/Wales/PHWC-PDTMKB/2021|EPI_ISL_4315253|2021-09-02

hCoV-19/Wales/PHWC-PDX8T5/2021|EPI_ISL_4318335|2021-09-03

hCoV-19/Wales/PHWC-PDXIQW/2021|EPI_ISL_4319121|2021-09-05

hCoV-19/Wales/PHWC-PD5HBQ/2021|EPI_ISL_4794465|2021-09-20

hCoV-19/Wales/PHWC-PD5WBS/2021|EPI_ISL_4795318|2021-09-20

hCoV-19/Wales/PHWC-PRYF6Y/2021|EPI_ISL_5049060|2021-09-26

hCoV-19/Wales/PHWC-PRDKSF/2021|EPI_ISL_5288295|2021-09-28

hCoV-19/Wales/ALDP-289B312/2021|EPI_ISL_6115290|2021-10-27

hCoV-19/Wales/PHWC-PFDF3D/2021|EPI_ISL_6284401|2021-11-03

hCoV-19/Wales/PHWC-PFNB7B/2021|EPI_ISL_6285225|2021-11-02

hCoV-19/Wales/PHWC-PFYNRZ/2021|EPI_ISL_6286535|2021-10-22

hCoV-19/Wales/PHWC-PR6FII/2021|EPI_ISL_6290794|2021-10-29

hCoV-19/Wales/PHWC-PGAOKM/2021|EPI_ISL_8852643|2021-12-27

H57V

hCoV-19/England/QEUH-29BA19A/2021|EPI_ISL_6295345|2021-11-08

hCoV-19/England/QEUH-29EDF85/2021|EPI_ISL_6344804|2021-11-09

hCoV-19/England/QEUH-29F203F/2021|EPI_ISL_6345965|2021-11-09

hCoV-19/England/QEUH-29F6AE2/2021|EPI_ISL_6351271|2021-11-09

hCoV-19/England/ALDP-2BE6A6B/2021|EPI_ISL_6919187|2021-11-25

hCoV-19/England/QEUH-2BE4229/2021|EPI_ISL_6919202|2021-11-24

hCoV-19/England/ALDP-2BEFBE7/2021|EPI_ISL_6919616|2021-11-24

hCoV-19/England/QEUH-2BBC1F0/2021|EPI_ISL_6924686|2021-11-24

hCoV-19/England/QEUH-2C0DB79/2021|EPI_ISL_7020840|2021-11-25

hCoV-19/England/QEUH-2C0DD73/2021|EPI_ISL_7020852|2021-11-25

hCoV-19/England/QEUH-2C11C33/2021|EPI_ISL_7022290|2021-11-26

hCoV-19/England/QEUH-2B87862/2021|EPI_ISL_7023880|2021-11-22

hCoV-19/England/ALDP-2BFA572/2021|EPI_ISL_7026565|2021-11-26

hCoV-19/England/QEUH-2C037BB/2021|EPI_ISL_7030992|2021-11-24

hCoV-19/England/NEWC-2C50DAE/2021|EPI_ISL_7183534|2021-11-27

hCoV-19/England/NEWC-2C4DCB6/2021|EPI_ISL_7183627|2021-11-27

hCoV-19/England/NEWC-2C52AF0/2021|EPI_ISL_7183907|2021-11-28

hCoV-19/England/NEWC-2C4DC10/2021|EPI_ISL_7183993|2021-11-27

hCoV-19/England/QEUH-2C4C992/2021|EPI_ISL_7186927|2021-11-28

hCoV-19/England/ALDP-2E164F8/2021|EPI_ISL_7761435|2021-12-09

hCoV-19/England/ALDP-2EA38B8/2021|EPI_ISL_7967246|2021-12-13

hCoV-19/England/ALDP-2F509C5/2021|EPI_ISL_8086960|2021-12-18

hCoV-19/England/ALDP-2F50B74/2021|EPI_ISL_8086981|2021-12-18

hCoV-19/England/ALDP-2FC2797/2021|EPI_ISL_8237855|2021-12-21

M57I

hCoV-19/Wales/PHWC-PDC87J/2021|EPI_ISL_4132200|2021-08-27

hCoV-19/Wales/PHWC-PDCHJ7/2021|EPI_ISL_4132495|2021-08-31

hCoV-19/Wales/PHWC-PDCUX4/2021|EPI_ISL_4132990|2021-08-27

hCoV-19/Wales/PHWC-PDKU69/2021|EPI_ISL_4135527|2021-08-26

hCoV-19/Wales/PHWC-PDKUCR/2021|EPI_ISL_4135533|2021-08-27

hCoV-19/Wales/PHWC-PDKUX6/2021|EPI_ISL_4135548|2021-08-27

hCoV-19/Wales/PHWC-PD16WZ/2021|EPI_ISL_4312768|2021-08-30

hCoV-19/Wales/PHWC-PDQXJG/2021|EPI_ISL_4314668|2021-08-31

hCoV-19/Wales/PHWC-PDQXKR/2021|EPI_ISL_4314669|2021-08-31

hCoV-19/Wales/PHWC-PDQXP6/2021|EPI_ISL_4314671|2021-08-31

hCoV-19/Wales/PHWC-PDQXX4/2021|EPI_ISL_4314676|2021-08-31

hCoV-19/Wales/PHWC-PDXRMM/2021|EPI_ISL_4319802|2021-09-04

hCoV-19/Wales/ALDP-1E9D544/2021|EPI_ISL_4454317|2021-09-16

hCoV-19/Wales/ALDP-1E9130A/2021|EPI_ISL_4454331|2021-09-14

hCoV-19/Wales/ALDP-1EB8D4B/2021|EPI_ISL_4503007|2021-09-14

hCoV-19/Wales/PHWC-PDADXT/2021|EPI_ISL_4796734|2021-09-12

hCoV-19/Wales/PHWC-PDSADA/2021|EPI_ISL_4800490|2021-08-26

hCoV-19/Wales/PHWC-PDZTPJ/2021|EPI_ISL_4803535|2021-09-09

hCoV-19/Wales/PHWC-PDZTQ8/2021|EPI_ISL_4803536|2021-09-09

hCoV-19/Wales/PHWC-PDZTTY/2021|EPI_ISL_4803548|2021-09-08

M62I

hCoV-19/England/ALDP-1634301/2021|EPI_ISL_2538130|2021-06-03

hCoV-19/England/ALDP-169AD47/2021|EPI_ISL_2605258|2021-06-07

hCoV-19/England/ALDP-1690A3B/2021|EPI_ISL_2606126|2021-06-08

hCoV-19/England/ALDP-170E624/2021|EPI_ISL_2664213|2021-06-15

hCoV-19/England/NORT-1BD44CF/2021|EPI_ISL_2706270|2021-06-15

hCoV-19/England/NORT-1BD6919/2021|EPI_ISL_2706624|2021-06-18

hCoV-19/England/SHEF-10D91A0/2021|EPI_ISL_2708439|2021-06-21

hCoV-19/England/SHEF-10E6EC7/2021|EPI_ISL_2708737|2021-06-21

hCoV-19/England/QEUH-1773D8F/2021|EPI_ISL_2718230|2021-06-21

hCoV-19/England/NORT-1BD8DC0/2021|EPI_ISL_2723484|2021-06-22

hCoV-19/England/NORW-13E828A/2021|EPI_ISL_2902700|2021-07-04

hCoV-19/England/NORW-13E8967/2021|EPI_ISL_2902793|2021-07-04

hCoV-19/England/ALDP-184FAFD/2021|EPI_ISL_2915557|2021-06-30

hCoV-19/England/ALDP-18AC609/2021|EPI_ISL_2973863|2021-07-05

hCoV-19/England/QEUH-1910F40/2021|EPI_ISL_2994562|2021-07-12

hCoV-19/England/ALDP-1993BE7/2021|EPI_ISL_3068656|2021-07-14

hCoV-19/England/QEUH-1969BAE/2021|EPI_ISL_3069975|2021-07-16

hCoV-19/England/MILK-19BC702/2021|EPI_ISL_3105926|2021-07-21

hCoV-19/England/ALDP-1989276/2021|EPI_ISL_3202111|2021-07-17

hCoV-19/England/PLYM-1A5FD1D/2021|EPI_ISL_3247972|2021-07-29

hCoV-19/England/ALDP-1AAA1DB/2021|EPI_ISL_3310591|2021-08-02

hCoV-19/England/QEUH-1AC1C91/2021|EPI_ISL_3338410|2021-08-02

hCoV-19/England/ALDP-1B00E17/2021|EPI_ISL_3359099|2021-08-05

hCoV-19/England/ALDP-1AEF143/2021|EPI_ISL_3362144|2021-08-03

hCoV-19/England/ALDP-1AE765B/2021|EPI_ISL_3365294|2021-08-04

hCoV-19/England/ALDP-1B10ED8/2021|EPI_ISL_3379989|2021-08-05

hCoV-19/England/NORW-3014D34/2021|EPI_ISL_3571820|2021-08-05

hCoV-19/England/PHEC-S303S4E0/2021|EPI_ISL_3574135|2021-07-24

hCoV-19/England/ALDP-1CE19DE/2021|EPI_ISL_3954756|2021-08-26

hCoV-19/England/NEWC-1B30BAE/2021|EPI_ISL_3977112|2021-08-08

hCoV-19/England/MILK-1D5C296/2021|EPI_ISL_4009833|2021-09-01

hCoV-19/England/PLYM-1D42336/2021|EPI_ISL_4010301|2021-09-01

hCoV-19/England/BRBR-1D37D18/2021|EPI_ISL_4012845|2021-08-31

hCoV-19/England/ALDP-1D46BD0/2021|EPI_ISL_4048116|2021-09-01

hCoV-19/England/MILK-1D69A8F/2021|EPI_ISL_4066376|2021-09-03

hCoV-19/England/NEWC-1D92E81/2021|EPI_ISL_4101387|2021-09-03

hCoV-19/England/MILK-1E1527A/2021|EPI_ISL_4247078|2021-09-10

hCoV-19/England/MILK-1E10DC9/2021|EPI_ISL_4248636|2021-09-10

hCoV-19/England/BRBR-1E2989F/2021|EPI_ISL_4288771|2021-09-10

hCoV-19/England/BRBR-1E6F6B1/2021|EPI_ISL_4351404|2021-09-14

hCoV-19/England/MILK-1E138F2/2021|EPI_ISL_4355158|2021-09-10

hCoV-19/England/MILK-1E9312C/2021|EPI_ISL_4405129|2021-09-16

hCoV-19/England/ALDP-1EBE03D/2021|EPI_ISL_4478718|2021-09-16

hCoV-19/England/ALDP-1EF9898/2021|EPI_ISL_4521926|2021-09-19

hCoV-19/England/ALDP-1ECEB1B/2021|EPI_ISL_4525437|2021-09-16

hCoV-19/England/ALDP-1F50F99/2021|EPI_ISL_4583274|2021-09-23

hCoV-19/England/BRBR-1F1A5E4/2021|EPI_ISL_4586525|2021-09-21

hCoV-19/England/ALDP-1F03EE3/2021|EPI_ISL_4623921|2021-09-16

hCoV-19/England/ALDP-1F9B8C4/2021|EPI_ISL_4752994|2021-09-24

hCoV-19/England/ALDP-1FA9E85/2021|EPI_ISL_4764081|2021-09-25

hCoV-19/England/NEWC-1FC1379/2021|EPI_ISL_4857107|2021-09-29

hCoV-19/England/ALDP-2006A60/2021|EPI_ISL_4975796|2021-10-01

hCoV-19/England/MILK-2052ACF/2021|EPI_ISL_5139553|2021-10-05

hCoV-19/England/MILK-2052898/2021|EPI_ISL_5140054|2021-10-05

hCoV-19/England/MILK-2052BEA/2021|EPI_ISL_5140131|2021-10-05

hCoV-19/England/ALDP-20DE7A6/2021|EPI_ISL_5257043|2021-10-10

hCoV-19/England/BRBR-2756DBC/2021|EPI_ISL_5357071|2021-10-14

hCoV-19/England/BRBR-276E601/2021|EPI_ISL_5408745|2021-10-14

hCoV-19/England/BRBR-2764C81/2021|EPI_ISL_5409148|2021-10-14

hCoV-19/England/PHEP-YYBK5HZ/2021|EPI_ISL_5490759|2021-10-12

hCoV-19/England/QEUH-284F061/2021|EPI_ISL_5783838|2021-10-25

hCoV-19/England/BRBR-284215C/2021|EPI_ISL_5785880|2021-10-24

hCoV-19/England/MILK-2832C6D/2021|EPI_ISL_5786309|2021-10-23

hCoV-19/England/QEUH-2827ABC/2021|EPI_ISL_5790032|2021-10-23

hCoV-19/England/LIVE-E1C22B/2021|EPI_ISL_5805811|2021-06-23

hCoV-19/England/QEUH-285B42A/2021|EPI_ISL_5834386|2021-10-24

hCoV-19/England/QEUH-285E6C7/2021|EPI_ISL_5893699|2021-10-25

hCoV-19/England/QEUH-2881D20/2021|EPI_ISL_5922042|2021-10-26

hCoV-19/England/QEUH-28A54C7/2021|EPI_ISL_5952389|2021-10-27

hCoV-19/England/QEUH-28936B7/2021|EPI_ISL_5962521|2021-10-26

hCoV-19/England/QEUH-28CED63/2021|EPI_ISL_5991192|2021-10-29

hCoV-19/England/ALDP-28C56E9/2021|EPI_ISL_5992649|2021-10-29

hCoV-19/England/QEUH-28C72D4/2021|EPI_ISL_5995647|2021-10-30

hCoV-19/England/QEUH-28EFAED/2021|EPI_ISL_6003400|2021-10-31

hCoV-19/England/QEUH-28E3463/2021|EPI_ISL_6007753|2021-10-31

hCoV-19/England/QEUH-28E36E5/2021|EPI_ISL_6007850|2021-10-31

hCoV-19/England/QEUH-28E3515/2021|EPI_ISL_6007919|2021-10-31

hCoV-19/England/ALDP-28B5109/2021|EPI_ISL_6008217|2021-10-28

hCoV-19/England/PHEC-3P077P01/2021|EPI_ISL_6025152|2021-10-14

hCoV-19/England/ALDP-28F8C45/2021|EPI_ISL_6041324|2021-11-01

hCoV-19/England/ALDP-28F8BDF/2021|EPI_ISL_6041508|2021-11-01

hCoV-19/England/ALDP-2905619/2021|EPI_ISL_6042895|2021-11-01

hCoV-19/England/QEUH-2949A86/2021|EPI_ISL_6148291|2021-11-03

hCoV-19/England/MILK-2937DFB/2021|EPI_ISL_6149236|2021-11-02

hCoV-19/England/QEUH-29B7E96/2021|EPI_ISL_6295370|2021-11-08

hCoV-19/England/QEUH-29BA349/2021|EPI_ISL_6295424|2021-11-08

hCoV-19/England/MILK-29AA5FB/2021|EPI_ISL_6296334|2021-11-07

hCoV-19/England/QEUH-299B609/2021|EPI_ISL_6296988|2021-11-07

hCoV-19/England/QEUH-29F0F9E/2021|EPI_ISL_6349872|2021-11-09

hCoV-19/England/QEUH-29C1822/2021|EPI_ISL_6354748|2021-11-08

hCoV-19/England/ALDP-2A137C9/2021|EPI_ISL_6430765|2021-11-10

hCoV-19/England/ALDP-2A13978/2021|EPI_ISL_6430850|2021-11-11

hCoV-19/England/BRBR-2AB87D5/2021|EPI_ISL_6620311|2021-11-15

hCoV-19/England/MILK-2B1A5A9/2021|EPI_ISL_6768376|2021-11-18

hCoV-19/England/PLYM-2B3DD6C/2021|EPI_ISL_6804991|2021-11-19

hCoV-19/England/MILK-2D77F04/2021|EPI_ISL_7572156|2021-12-06

hCoV-19/England/MILK-2D78185/2021|EPI_ISL_7572183|2021-12-06

hCoV-19/England/ALDP-2EAB470/2021|EPI_ISL_7971473|2021-12-13

hCoV-19/England/LSPA-2F26C83/2021|EPI_ISL_8072275|2021-12-15

hCoV-19/England/QEUH-2FCF2F3/2021|EPI_ISL_8278267|2021-12-21

hCoV-19/England/ALDP-30BDC23/2021|EPI_ISL_8492164|2021-12-29

hCoV-19/England/ALDP-3151CC6/2022|EPI_ISL_8656760|2022-01-05

M62T

hCoV-19/Denmark/DCGC-87617/2021|EPI_ISL_2023802|2021-05-03

hCoV-19/Denmark/DCGC-87692/2021|EPI_ISL_2023877|2021-04-26

hCoV-19/Denmark/DCGC-87737/2021|EPI_ISL_2023922|2021-04-26

hCoV-19/Denmark/DCGC-88870/2021|EPI_ISL_2025084|2021-05-03

hCoV-19/Denmark/DCGC-88966/2021|EPI_ISL_2025183|2021-05-03

hCoV-19/Denmark/DCGC-89096/2021|EPI_ISL_2025313|2021-04-26

hCoV-19/Denmark/DCGC-89393/2021|EPI_ISL_2025610|2021-04-26

hCoV-19/Denmark/DCGC-89963/2021|EPI_ISL_2026186|2021-04-26

hCoV-19/Denmark/DCGC-90310/2021|EPI_ISL_2026569|2021-04-26

hCoV-19/Denmark/DCGC-90984/2021|EPI_ISL_2027274|2021-04-26

hCoV-19/Denmark/DCGC-92740/2021|EPI_ISL_2172583|2021-05-10

hCoV-19/Denmark/DCGC-93918/2021|EPI_ISL_2173763|2021-05-10

hCoV-19/Denmark/DCGC-94282/2021|EPI_ISL_2174128|2021-05-10

hCoV-19/Denmark/DCGC-94886/2021|EPI_ISL_2174733|2021-05-10

hCoV-19/Denmark/DCGC-94944/2021|EPI_ISL_2174791|2021-05-10

hCoV-19/Denmark/DCGC-95008/2021|EPI_ISL_2174855|2021-05-03

hCoV-19/Denmark/DCGC-95212/2021|EPI_ISL_2175059|2021-05-10

hCoV-19/Denmark/DCGC-95322/2021|EPI_ISL_2175169|2021-05-10

hCoV-19/Denmark/DCGC-96057/2021|EPI_ISL_2175905|2021-05-03

hCoV-19/Denmark/DCGC-96354/2021|EPI_ISL_2176202|2021-05-03

hCoV-19/Denmark/DCGC-97400/2021|EPI_ISL_2177287|2021-05-10

hCoV-19/Denmark/DCGC-97429/2021|EPI_ISL_2177316|2021-05-03

hCoV-19/Denmark/DCGC-97594/2021|EPI_ISL_2177557|2021-05-03

hCoV-19/Denmark/DCGC-97664/2021|EPI_ISL_2177627|2021-05-03

hCoV-19/Denmark/DCGC-100672/2021|EPI_ISL_2298488|2021-05-10

hCoV-19/Denmark/DCGC-101302/2021|EPI_ISL_2299046|2021-05-17

hCoV-19/Denmark/DCGC-101557/2021|EPI_ISL_2299226|2021-05-17

hCoV-19/Denmark/DCGC-102909/2021|EPI_ISL_2300406|2021-05-10

hCoV-19/Denmark/DCGC-103315/2021|EPI_ISL_2300820|2021-05-10

hCoV-19/Denmark/DCGC-104251/2021|EPI_ISL_2411649|2021-05-17

hCoV-19/Denmark/DCGC-104830/2021|EPI_ISL_2412334|2021-05-17

hCoV-19/Denmark/DCGC-106642/2021|EPI_ISL_2414656|2021-05-24

hCoV-19/Denmark/DCGC-106725/2021|EPI_ISL_2414750|2021-05-17

hCoV-19/Denmark/DCGC-106802/2021|EPI_ISL_2414839|2021-05-24

hCoV-19/Denmark/DCGC-107722/2021|EPI_ISL_2415966|2021-05-10

hCoV-19/Denmark/DCGC-108773/2021|EPI_ISL_2417266|2021-05-17

hCoV-19/Denmark/DCGC-109081/2021|EPI_ISL_2417643|2021-05-17

hCoV-19/Denmark/DCGC-109511/2021|EPI_ISL_2418160|2021-05-24

hCoV-19/Denmark/DCGC-104739/2021|EPI_ISL_2429545|2021-05-24

hCoV-19/Denmark/DCGC-110811/2021|EPI_ISL_2665435|2021-05-17

hCoV-19/Denmark/DCGC-111572/2021|EPI_ISL_2665998|2021-05-31

hCoV-19/Denmark/DCGC-111945/2021|EPI_ISL_2666284|2021-05-24

hCoV-19/Denmark/DCGC-111947/2021|EPI_ISL_2666285|2021-05-31

hCoV-19/Denmark/DCGC-111949/2021|EPI_ISL_2666287|2021-06-07

hCoV-19/Denmark/DCGC-112314/2021|EPI_ISL_2666546|2021-06-14

hCoV-19/Denmark/DCGC-112617/2021|EPI_ISL_2666767|2021-05-31

hCoV-19/Denmark/DCGC-112638/2021|EPI_ISL_2666784|2021-05-31

hCoV-19/Denmark/DCGC-112752/2021|EPI_ISL_2666862|2021-06-14

hCoV-19/Denmark/DCGC-112998/2021|EPI_ISL_2667040|2021-05-31

hCoV-19/Denmark/DCGC-113697/2021|EPI_ISL_2667536|2021-06-14

hCoV-19/Denmark/DCGC-113739/2021|EPI_ISL_2667564|2021-06-07

hCoV-19/Denmark/DCGC-114704/2021|EPI_ISL_2668270|2021-05-24

hCoV-19/Denmark/DCGC-115371/2021|EPI_ISL_2668758|2021-06-07

hCoV-19/Denmark/DCGC-115656/2021|EPI_ISL_2668951|2021-06-07

hCoV-19/Denmark/DCGC-115816/2021|EPI_ISL_2669061|2021-06-07

hCoV-19/Denmark/DCGC-116413/2021|EPI_ISL_2669488|2021-05-31

hCoV-19/Denmark/DCGC-116951/2021|EPI_ISL_2669861|2021-06-14

hCoV-19/Denmark/DCGC-117114/2021|EPI_ISL_2669984|2021-05-31

hCoV-19/Denmark/DCGC-117659/2021|EPI_ISL_2670358|2021-05-31

hCoV-19/Denmark/DCGC-117750/2021|EPI_ISL_2670421|2021-05-31

hCoV-19/Denmark/DCGC-117885/2021|EPI_ISL_2670523|2021-05-31

hCoV-19/Denmark/DCGC-118141/2021|EPI_ISL_2670702|2021-06-07

hCoV-19/Denmark/DCGC-118284/2021|EPI_ISL_2670805|2021-06-07

hCoV-19/Denmark/DCGC-118294/2021|EPI_ISL_2670813|2021-06-07

hCoV-19/Denmark/DCGC-118942/2021|EPI_ISL_2671288|2021-06-14

hCoV-19/Denmark/DCGC-104837/2021|EPI_ISL_2683592|2021-05-24

hCoV-19/Denmark/DCGC-105429/2021|EPI_ISL_2683662|2021-05-24

hCoV-19/Denmark/DCGC-107172/2021|EPI_ISL_2683899|2021-05-24

hCoV-19/Denmark/DCGC-107865/2021|EPI_ISL_2683977|2021-05-17

hCoV-19/Denmark/DCGC-108761/2021|EPI_ISL_2684084|2021-05-24

hCoV-19/Denmark/DCGC-109419/2021|EPI_ISL_2684160|2021-05-17

hCoV-19/Denmark/DCGC-119115/2021|EPI_ISL_2684302|2021-06-07

hCoV-19/Denmark/DCGC-119274/2021|EPI_ISL_2684423|2021-05-31

hCoV-19/Denmark/DCGC-119485/2021|EPI_ISL_2684574|2021-05-24

hCoV-19/Denmark/DCGC-119712/2021|EPI_ISL_2684738|2021-06-14

hCoV-19/Denmark/DCGC-119871/2021|EPI_ISL_2684857|2021-05-31

hCoV-19/Denmark/DCGC-120083/2021|EPI_ISL_2685001|2021-06-07

hCoV-19/Denmark/DCGC-120100/2021|EPI_ISL_2685016|2021-06-07

hCoV-19/Denmark/DCGC-120149/2021|EPI_ISL_2685057|2021-05-31

hCoV-19/Denmark/DCGC-120224/2021|EPI_ISL_2685118|2021-06-07

hCoV-19/Denmark/DCGC-120427/2021|EPI_ISL_2685267|2021-06-07

hCoV-19/Denmark/DCGC-120497/2021|EPI_ISL_2685323|2021-06-14

hCoV-19/Denmark/DCGC-120504/2021|EPI_ISL_2685330|2021-06-07

hCoV-19/Denmark/DCGC-120893/2021|EPI_ISL_2685622|2021-05-31

hCoV-19/Denmark/DCGC-121139/2021|EPI_ISL_2685809|2021-06-07

hCoV-19/Denmark/DCGC-121416/2021|EPI_ISL_2747785|2021-06-21

hCoV-19/Denmark/DCGC-121594/2021|EPI_ISL_2748014|2021-06-24

hCoV-19/Denmark/DCGC-122134/2021|EPI_ISL_2784466|2021-06-23

hCoV-19/Denmark/DCGC-122315/2021|EPI_ISL_2784592|2021-06-26

hCoV-19/Denmark/DCGC-122379/2021|EPI_ISL_2784643|2021-06-25

hCoV-19/Denmark/DCGC-125172/2021|EPI_ISL_2896562|2021-07-06

hCoV-19/Denmark/DCGC-132359/2021|EPI_ISL_3041338|2021-07-15

hCoV-19/Denmark/DCGC-115956/2021|EPI_ISL_3074870|2021-05-24

hCoV-19/Denmark/DCGC-116041/2021|EPI_ISL_3074885|2021-05-31

hCoV-19/Denmark/DCGC-119807/2021|EPI_ISL_3075635|2021-06-07

hCoV-19/Denmark/DCGC-122189/2021|EPI_ISL_3075873|2021-06-25

hCoV-19/Denmark/DCGC-122968/2021|EPI_ISL_3076045|2021-06-29

hCoV-19/Denmark/DCGC-124036/2021|EPI_ISL_3076189|2021-07-02

hCoV-19/Denmark/DCGC-124752/2021|EPI_ISL_3076307|2021-07-03

hCoV-19/Denmark/DCGC-146250/2021|EPI_ISL_3339821|2021-06-30

M62V

hCoV-19/France/un-HMN-21022080726/2021|EPI_ISL_1085600|2021-02-08

hCoV-19/France/ARA-HCL021033503701/2021|EPI_ISL_1265803|2021-02-20

hCoV-19/France/IDF-HMN-21032090260/2021|EPI_ISL_1396835|2021-03-02

hCoV-19/France/IDF-IPP06545/2021|EPI_ISL_1417206|2021-03-16

hCoV-19/France/IDF-HMN-21032220647/2021|EPI_ISL_1754399|2021-03-16

hCoV-19/France/HDF-P054-21145M1187/2021|EPI_ISL_1818834|2021-04-07

hCoV-19/France/IDF-HMN-21042200724/2021|EPI_ISL_1821847|2021-04-20

hCoV-19/France/HDF-P157-21161M0769/2021|EPI_ISL_1920221|2021-04-19

hCoV-19/France/HDF-P155-21161M0801/2021|EPI_ISL_1920226|2021-04-17

hCoV-19/France/PDL-IPP11309/2021|EPI_ISL_2029609|2021-04-27

hCoV-19/France/PDL-IPP11330/2021|EPI_ISL_2029645|2021-04-27

hCoV-19/France/PDL-IPP11345/2021|EPI_ISL_2029847|2021-04-27

hCoV-19/France/IDF-IPP10826/2021|EPI_ISL_2029890|2021-04-27

hCoV-19/France/HDF-P310-21182C4555/2021|EPI_ISL_2143385|2021-05-04

hCoV-19/France/HDF-P310-21182C4555/2021|EPI_ISL_2143386|2021-05-04

hCoV-19/France/HDF-P310-21182C4555/2021|EPI_ISL_2143388|2021-05-04

hCoV-19/France/HDF-P305-21182C5353/2021|EPI_ISL_2143398|2021-05-04

hCoV-19/France/HDF-P302-21177C1553/2021|EPI_ISL_2143451|2021-05-02

hCoV-19/France/HDF-P351-21172M0982/2021|EPI_ISL_2143474|2021-04-26

hCoV-19/France/HDF-P310-21182C4555/2021|EPI_ISL_2143502|2021-05-04

hCoV-19/France/HDF-P305-21182C5353/2021|EPI_ISL_2143516|2021-05-04

hCoV-19/France/HDF-P302-21177C1553/2021|EPI_ISL_2143590|2021-05-02

hCoV-19/France/HDF-P351-21172M0982/2021|EPI_ISL_2143616|2021-04-26

hCoV-19/France/PDL-IPP11527/2021|EPI_ISL_2188371|2021-04-30

hCoV-19/France/HDF-P146-21201M1171/2021|EPI_ISL_2361557|2021-05-16

hCoV-19/France/OCC-CHU-TLS-210821397001/2021|EPI_ISL_2366978|2021-03-15

hCoV-19/France/OCC-CHU-TLS-210821380501/2021|EPI_ISL_2367029|2021-03-16

hCoV-19/France/HDF-P343-21214M0973/2021|EPI_ISL_2501287|2021-05-26

hCoV-19/France/HDF-IPP13359/2021|EPI_ISL_2535499|2021-05-25

M195I

hCoV-19/England/MILK-379497F/2022|EPI_ISL_10335805|2022-02-19

hCoV-19/England/MILK-37EAB0B/2022|EPI_ISL_10467380|2022-02-21

hCoV-19/England/MILK-3863547/2022|EPI_ISL_10644949|2022-02-24

hCoV-19/England/MILK-38AD75A/2022|EPI_ISL_10724925|2022-02-28

hCoV-19/England/MILK-388BD7E/2022|EPI_ISL_10727470|2022-02-27

hCoV-19/England/MILK-38CB0E2/2022|EPI_ISL_10797018|2022-03-02

hCoV-19/England/MILK-38B6132/2022|EPI_ISL_10799248|2022-03-01

hCoV-19/England/MILK-38D36E5/2022|EPI_ISL_10850545|2022-03-02

hCoV-19/England/MILK-3901146/2022|EPI_ISL_10887688|2022-03-03

hCoV-19/England/LSPA-391509D/2022|EPI_ISL_10930017|2022-03-03

hCoV-19/England/LSPA-3941FF5/2022|EPI_ISL_10965828|2022-03-04

hCoV-19/England/MILK-397FC60/2022|EPI_ISL_10970089|2022-03-05

hCoV-19/England/MILK-397AEE7/2022|EPI_ISL_10971384|2022-03-06

hCoV-19/England/LSPA-3943D35/2022|EPI_ISL_10975978|2022-03-05

hCoV-19/England/LSPA-3944068/2022|EPI_ISL_10976107|2022-03-05

hCoV-19/England/LSPA-39A55EE/2022|EPI_ISL_10987864|2022-03-06

hCoV-19/England/MILK-39C2590/2022|EPI_ISL_11033167|2022-03-07

hCoV-19/England/MILK-3A017F8/2022|EPI_ISL_11078333|2022-03-10

hCoV-19/England/MILK-39B201A/2022|EPI_ISL_11092539|2022-03-08

hCoV-19/England/MILK-39B08C9/2022|EPI_ISL_11092564|2022-03-08

hCoV-19/England/PHEC-YYDR595/2022|EPI_ISL_11111988|2022-03-02

hCoV-19/England/LSPA-3A33924/2022|EPI_ISL_11121859|2022-03-11

hCoV-19/England/MILK-3A08041/2022|EPI_ISL_11124930|2022-03-09

hCoV-19/England/MILK-3A13AFA/2022|EPI_ISL_11129141|2022-03-10

hCoV-19/England/MILK-3A315F5/2022|EPI_ISL_11151254|2022-03-12

hCoV-19/England/NEWC-3A64517/2022|EPI_ISL_11181763|2022-03-07

hCoV-19/England/MILK-3A6B668/2022|EPI_ISL_11182397|2022-03-12

hCoV-19/England/ALDP-3A6FA3B/2022|EPI_ISL_11185308|2022-03-11

hCoV-19/England/MILK-3A9E769/2022|EPI_ISL_11196446|2022-03-13

hCoV-19/England/MILK-3ABCCDF/2022|EPI_ISL_11212593|2022-03-13

hCoV-19/England/LSPA-3A736F4/2022|EPI_ISL_11275033|2022-03-12

hCoV-19/England/LSPA-3AE71AF/2022|EPI_ISL_11308533|2022-03-15

hCoV-19/England/LSPA-3B0CA7B/2022|EPI_ISL_11337767|2022-03-15

hCoV-19/England/MILK-3B581F3/2022|EPI_ISL_11388635|2022-03-18

hCoV-19/England/MILK-3B4E178/2022|EPI_ISL_11392571|2022-03-18

hCoV-19/England/LSPA-3B77677/2022|EPI_ISL_11418219|2022-03-18

hCoV-19/England/LSPA-3B85A7A/2022|EPI_ISL_11419345|2022-03-19

hCoV-19/England/BRBR-3B86C0A/2022|EPI_ISL_11419403|2022-03-19

hCoV-19/England/LSPA-3BA859E/2022|EPI_ISL_11429534|2022-03-21

hCoV-19/England/LSPA-3B9436E/2022|EPI_ISL_11431069|2022-03-18

hCoV-19/England/LSPA-3C0FA68/2022|EPI_ISL_11544000|2022-03-23

hCoV-19/England/ALDP-3C23248/2022|EPI_ISL_11597973|2022-03-23

hCoV-19/England/LSPA-3C72857/2022|EPI_ISL_11641929|2022-03-25

hCoV-19/England/ALDP-3C41B2B/2022|EPI_ISL_11642421|2022-03-24

hCoV-19/England/LSPA-3C91DCC/2022|EPI_ISL_11689299|2022-03-26

hCoV-19/England/LSPA-3CA3583/2022|EPI_ISL_11727359|2022-03-25

hCoV-19/England/LSPA-3CB0592/2022|EPI_ISL_11731763|2022-03-28

hCoV-19/England/PHEC-YYD3437/2022|EPI_ISL_11828914|2022-03-21

hCoV-19/England/LSPA-3D058FE/2022|EPI_ISL_11831015|2022-03-31

hCoV-19/England/PHEC-YYD4QR1/2022|EPI_ISL_11877209|2022-03-23

hCoV-19/England/PHEC-YYRC9F5/2022|EPI_ISL_12102566|2022-03-23

hCoV-19/England/PHEC-YYR1EM3/2022|EPI_ISL_12221877|2022-04-07

hCoV-19/England/QEUH-3CEA061/2022|EPI_ISL_12776389|2022-03-24

hCoV-19/England/QEUH-3CE11C1/2022|EPI_ISL_12776593|2022-03-24

M195T

hCoV-19/Wales/PHWC-PY169R/2021|EPI_ISL_3081489|2021-07-14

hCoV-19/Wales/PHWC-PY19E1/2021|EPI_ISL_3081534|2021-07-14

hCoV-19/Wales/PHWC-PYUZ1D/2021|EPI_ISL_3083737|2021-07-14

hCoV-19/Wales/PHWC-PYWJKY/2021|EPI_ISL_3083908|2021-07-13

hCoV-19/Wales/PHWC-PYWU7X/2021|EPI_ISL_3084091|2021-07-16

hCoV-19/Wales/PHWC-PYWU9M/2021|EPI_ISL_3084093|2021-07-15

hCoV-19/Wales/PHWC-PYWW1R/2021|EPI_ISL_3084105|2021-07-15

hCoV-19/Wales/PHWC-PYWWOE/2021|EPI_ISL_3084122|2021-07-15

hCoV-19/Wales/PHWC-PYWWTG/2021|EPI_ISL_3084125|2021-07-15

hCoV-19/Wales/PHWC-PYWX6T/2021|EPI_ISL_3084131|2021-07-15

hCoV-19/Wales/PHWC-PYIPQ1/2021|EPI_ISL_3181080|2021-07-18

hCoV-19/Wales/PHWC-PYSK61/2021|EPI_ISL_3181745|2021-07-20

hCoV-19/Wales/PHWC-PYSKPI/2021|EPI_ISL_3181757|2021-07-20

hCoV-19/Wales/PHWC-PY3I6B/2021|EPI_ISL_3293791|2021-07-22

hCoV-19/Wales/PHWC-PY3IHF/2021|EPI_ISL_3293800|2021-07-22

hCoV-19/Wales/PHWC-PY3PYG/2021|EPI_ISL_3293961|2021-07-26

hCoV-19/Wales/PHWC-PY3QWH/2021|EPI_ISL_3293984|2021-07-21

hCoV-19/Wales/PHWC-PY3XRH/2021|EPI_ISL_3294144|2021-07-19

hCoV-19/Wales/PHWC-PY3Z8Q/2021|EPI_ISL_3294184|2021-07-23

hCoV-19/Wales/PHWC-PY3ZCR/2021|EPI_ISL_3294188|2021-07-23

hCoV-19/Wales/PHWC-PY3ZGO/2021|EPI_ISL_3294190|2021-07-22

hCoV-19/Wales/PHWC-PY4EMU/2021|EPI_ISL_3294498|2021-07-24

hCoV-19/Wales/PHWC-PYA5EK/2021|EPI_ISL_3294993|2021-07-25

hCoV-19/Wales/PHWC-PYA5WT/2021|EPI_ISL_3295006|2021-07-25

hCoV-19/Wales/PHWC-PYADTX/2021|EPI_ISL_3295158|2021-07-24

hCoV-19/Wales/PHWC-PYAOBD/2021|EPI_ISL_3295393|2021-07-19

hCoV-19/Wales/PHWC-PYAOKT/2021|EPI_ISL_3295401|2021-07-19

hCoV-19/Wales/PHWC-PYAOMX/2021|EPI_ISL_3295402|2021-07-19

hCoV-19/Wales/PHWC-PYAONB/2021|EPI_ISL_3295403|2021-07-19

hCoV-19/Wales/PHWC-PYAQW6/2021|EPI_ISL_3295460|2021-07-19

hCoV-19/Wales/PHWC-PYAX98/2021|EPI_ISL_3295702|2021-07-19

hCoV-19/Wales/PHWC-PYAXNN/2021|EPI_ISL_3295723|2021-07-19

hCoV-19/Wales/PHWC-PYH4NX/2021|EPI_ISL_3295955|2021-07-28

hCoV-19/Wales/PHWC-PYSI9F/2021|EPI_ISL_3297625|2021-07-21

hCoV-19/Wales/PHWC-PYSIYR/2021|EPI_ISL_3297642|2021-07-21

hCoV-19/Wales/PHWC-PYSSTY/2021|EPI_ISL_3297752|2021-07-21

hCoV-19/Wales/ALDP-1ABD199/2021|EPI_ISL_3338358|2021-08-02

hCoV-19/Wales/PHWC-PDBD3M/2021|EPI_ISL_3422366|2021-08-08

hCoV-19/Wales/PHWC-PY6BJS/2021|EPI_ISL_3423440|2021-07-30

hCoV-19/Wales/PHWC-PY7IEG/2021|EPI_ISL_3424118|2021-08-02

hCoV-19/Wales/PHWC-PY7IFC/2021|EPI_ISL_3424119|2021-08-02

hCoV-19/Wales/PHWC-PY95DG/2021|EPI_ISL_3424403|2021-07-27

hCoV-19/Wales/PHWC-PY9MKE/2021|EPI_ISL_3424803|2021-08-03

hCoV-19/Wales/QEUH-1AFD35B/2021|EPI_ISL_3446517|2021-08-04

hCoV-19/Wales/QEUH-1B38BA6/2021|EPI_ISL_3455356|2021-08-08

hCoV-19/Wales/ALDP-1B336E6/2021|EPI_ISL_3455357|2021-08-05

hCoV-19/Wales/LSPA-1A0E506/2021|EPI_ISL_3499956|2021-07-23

hCoV-19/Wales/PHWC-PDD3AU/2021|EPI_ISL_3785973|2021-08-13

hCoV-19/Wales/PHWC-PDD4GZ/2021|EPI_ISL_3786149|2021-08-13

hCoV-19/Wales/PHWC-PDDHET/2021|EPI_ISL_3787456|2021-08-11

hCoV-19/Wales/PHWC-PDFOZ4/2021|EPI_ISL_3789766|2021-08-16

hCoV-19/Wales/PHWC-PDFTBG/2021|EPI_ISL_3789938|2021-08-16

hCoV-19/Wales/PHWC-PDR3YN/2021|EPI_ISL_3790827|2021-08-09

hCoV-19/Wales/PHWC-PD8MHT/2021|EPI_ISL_3963065|2021-08-15

hCoV-19/Wales/PHWC-PDJJCO/2021|EPI_ISL_4134345|2021-08-23

Q22H

hCoV-19/England/ALDP-1652DE1/2021|EPI_ISL_2541855|2021-06-05

hCoV-19/England/PLYM-178691C/2021|EPI_ISL_2729809|2021-06-22

hCoV-19/England/QEUH-18073D8/2021|EPI_ISL_2850083|2021-06-29

hCoV-19/England/PLYM-1825EE2/2021|EPI_ISL_2867186|2021-07-01

hCoV-19/England/QEUH-1898F23/2021|EPI_ISL_2935797|2021-07-05

hCoV-19/England/ALDP-18CB72F/2021|EPI_ISL_2974103|2021-07-07

hCoV-19/England/ALDP-18CB67D/2021|EPI_ISL_2974142|2021-07-07

hCoV-19/England/ALDP-1911422/2021|EPI_ISL_2994420|2021-07-11

hCoV-19/England/PLYM-195071F/2021|EPI_ISL_3042171|2021-07-15

hCoV-19/England/ALDP-194864A/2021|EPI_ISL_3051686|2021-07-12

hCoV-19/England/ALDP-19958A2/2021|EPI_ISL_3093082|2021-07-18

hCoV-19/England/ALDP-19CBDD1/2021|EPI_ISL_3107503|2021-07-17

hCoV-19/England/ALDP-1983397/2021|EPI_ISL_3109110|2021-07-16

hCoV-19/England/ALDP-19A8F4D/2021|EPI_ISL_3109363|2021-07-20

hCoV-19/England/ALDP-19CE749/2021|EPI_ISL_3125228|2021-07-21

hCoV-19/England/ALDP-1A148D7/2021|EPI_ISL_3248354|2021-07-25

hCoV-19/England/NORT-1BE9EBA/2021|EPI_ISL_3288515|2021-07-27

hCoV-19/England/NORW-13F8894/2021|EPI_ISL_3289525|2021-07-23

hCoV-19/England/ALDP-1B60E06/2021|EPI_ISL_3437761|2021-08-10

hCoV-19/England/ALDP-1B6EEDB/2021|EPI_ISL_3454121|2021-08-11

hCoV-19/England/ALDP-1BB2078/2021|EPI_ISL_3529553|2021-08-14

hCoV-19/England/PLYM-1C0138F/2021|EPI_ISL_3702962|2021-08-17

hCoV-19/England/MILK-1CA8BA0/2021|EPI_ISL_3884292|2021-08-25

hCoV-19/England/MILK-1CA8CDA/2021|EPI_ISL_3884349|2021-08-25

hCoV-19/England/BRBR-1CC5DC5/2021|EPI_ISL_3954210|2021-08-26

hCoV-19/England/ALDP-1CC97E1/2021|EPI_ISL_3956403|2021-08-26

hCoV-19/England/ALDP-1D12C70/2021|EPI_ISL_3992114|2021-08-30

hCoV-19/England/MILK-1D16DD3/2021|EPI_ISL_4015181|2021-08-30

hCoV-19/England/MILK-1D16A27/2021|EPI_ISL_4015273|2021-08-30

hCoV-19/England/MILK-1DD78FB/2021|EPI_ISL_4190928|2021-09-07

hCoV-19/England/MILK-1DD7907/2021|EPI_ISL_4191101|2021-09-07

hCoV-19/England/BRBR-1E0D73A/2021|EPI_ISL_4247301|2021-09-09

hCoV-19/England/NORW-3034DCF/2021|EPI_ISL_4307769|2021-09-09

hCoV-19/England/MILK-1E6B442/2021|EPI_ISL_4322745|2021-09-14

hCoV-19/England/NEWC-1E3114E/2021|EPI_ISL_4328397|2021-09-10

hCoV-19/England/NEWC-1E8092A/2021|EPI_ISL_4407776|2021-09-14

hCoV-19/England/QEUH-1EA4DE8/2021|EPI_ISL_4453543|2021-09-16

hCoV-19/England/ALDP-1ED7FB9/2021|EPI_ISL_4501702|2021-09-17

hCoV-19/England/ALDP-1EF1ECB/2021|EPI_ISL_4522314|2021-09-19

hCoV-19/England/MILK-1EE6B1D/2021|EPI_ISL_4524981|2021-09-20

hCoV-19/England/PHEC-YYBKAIE/2021|EPI_ISL_4530981|2021-09-09

hCoV-19/England/PHEC-Z30AZ4F8/2021|EPI_ISL_4531710|2021-09-16

hCoV-19/England/PLYM-1F57F92/2021|EPI_ISL_4618717|2021-09-24

hCoV-19/England/ALDP-1F66D2D/2021|EPI_ISL_4675525|2021-09-24

hCoV-19/England/MILK-1F5EBF1/2021|EPI_ISL_4736036|2021-09-23

hCoV-19/England/MILK-1F9686F/2021|EPI_ISL_4755759|2021-09-27

hCoV-19/England/ALDP-1FB8102/2021|EPI_ISL_4762490|2021-09-24

hCoV-19/England/ALDP-1FAF8AF/2021|EPI_ISL_4764533|2021-09-25

hCoV-19/England/ALDP-1F7D048/2021|EPI_ISL_4875304|2021-09-25

hCoV-19/England/QEUH-200517B/2021|EPI_ISL_4974313|2021-09-30

hCoV-19/England/MILK-203D480/2021|EPI_ISL_5023432|2021-10-03

hCoV-19/England/MILK-20833D9/2021|EPI_ISL_5142364|2021-10-06

hCoV-19/England/MILK-2755D80/2021|EPI_ISL_5350397|2021-10-12

hCoV-19/England/MILK-284D7B9/2021|EPI_ISL_5784616|2021-10-24

hCoV-19/England/PHEC-3M0AEMEB/2021|EPI_ISL_5809505|2021-10-19

hCoV-19/England/ALDP-28724C3/2021|EPI_ISL_5896761|2021-10-26

hCoV-19/England/ALDP-2871901/2021|EPI_ISL_5922381|2021-10-26

hCoV-19/England/ALDP-28B69C4/2021|EPI_ISL_5949369|2021-10-28

hCoV-19/England/MILK-289951E/2021|EPI_ISL_5993527|2021-10-25

hCoV-19/England/ALDP-28EEF58/2021|EPI_ISL_6003251|2021-10-31

hCoV-19/England/PHEC-3R07CR3F/2021|EPI_ISL_6030796|2021-10-25

hCoV-19/England/PHEC-3R07CR4E/2021|EPI_ISL_6030797|2021-10-25

hCoV-19/England/ALDP-294C4EF/2021|EPI_ISL_6148004|2021-11-04

hCoV-19/England/MILK-292A1FF/2021|EPI_ISL_6150449|2021-11-03

hCoV-19/England/ALDP-29422D0/2021|EPI_ISL_6225412|2021-11-03

hCoV-19/England/QEUH-299FF73/2021|EPI_ISL_6300437|2021-11-06

hCoV-19/England/ALDP-298AAD4/2021|EPI_ISL_6303204|2021-11-06

hCoV-19/England/ALDP-29E0D6A/2021|EPI_ISL_6351190|2021-11-08

hCoV-19/England/MILK-29CD03D/2021|EPI_ISL_6354392|2021-11-08

hCoV-19/England/ALDP-2ACECA0/2021|EPI_ISL_6611212|2021-11-16

hCoV-19/England/ALDP-2AD81A4/2021|EPI_ISL_6671047|2021-11-17

hCoV-19/England/ALDP-2B0B796/2021|EPI_ISL_6757680|2021-11-18

hCoV-19/England/QEUH-2BBF834/2021|EPI_ISL_6925277|2021-11-24

hCoV-19/England/LOND-13567F1/2021|EPI_ISL_6972904|2021-11-11

hCoV-19/England/PHEP-YYNBAQE/2021|EPI_ISL_6986906|2021-11-15

hCoV-19/England/QEUH-2C0DBE2/2021|EPI_ISL_7020916|2021-11-25

hCoV-19/England/ALDP-2BE4CEF/2021|EPI_ISL_7029849|2021-11-25

hCoV-19/England/ALDP-2CA4312/2021|EPI_ISL_7296831|2021-11-30

hCoV-19/England/ALDP-2CB2302/2021|EPI_ISL_7346360|2021-11-30

hCoV-19/England/QEUH-2CB2979/2021|EPI_ISL_7353136|2021-11-30

hCoV-19/England/QEUH-2CB2AA3/2021|EPI_ISL_7353234|2021-11-30

hCoV-19/England/ALDP-2CF5084/2021|EPI_ISL_7389572|2021-12-02

hCoV-19/England/QEUH-2CF541F/2021|EPI_ISL_7389689|2021-11-30

hCoV-19/England/PHEP-YYN89C3/2021|EPI_ISL_7464170|2021-11-22

hCoV-19/England/PHEP-YYN89QI/2021|EPI_ISL_7464174|2021-11-22

hCoV-19/England/ALDP-2D1C806/2021|EPI_ISL_7540985|2021-12-02

hCoV-19/England/BRBR-2D33D55/2021|EPI_ISL_7541319|2021-12-01

hCoV-19/England/MILK-2D620AF/2021|EPI_ISL_7555666|2021-12-05

hCoV-19/England/ALDP-2DABAF7/2021|EPI_ISL_7631040|2021-12-07

hCoV-19/England/MILK-2D80DD1/2021|EPI_ISL_7654789|2021-12-06

hCoV-19/England/PHEC-YYB1G73/2021|EPI_ISL_7771807|2021-12-04

hCoV-19/England/ALDP-2E298D8/2021|EPI_ISL_7819641|2021-12-10

hCoV-19/England/PHEC-YYB1IOR/2021|EPI_ISL_7823131|2021-12-03

hCoV-19/England/MILK-2E70E3A/2021|EPI_ISL_7863212|2021-12-13

hCoV-19/England/MILK-2E74470/2021|EPI_ISL_7864941|2021-12-13

hCoV-19/England/MILK-2EEEC3D/2021|EPI_ISL_8048597|2021-12-15

hCoV-19/England/ALDP-2CE9C52/2021|EPI_ISL_8052466|2021-12-02

hCoV-19/England/ALDP-2F25F12/2021|EPI_ISL_8072472|2021-12-18

hCoV-19/England/PHEC-YYBWAN4/2021|EPI_ISL_8090217|2021-12-14

hCoV-19/England/LSPA-3059245/2021|EPI_ISL_8452919|2021-12-27

Recombination dataset

hCoV-19/Mexico/QUE-IBT-IMSS-507/2020

hCoV-19/Senegal/B34b12_N335332798/2022

hCoV-19/USA/IA-GMF-33487/2020

hCoV-19/Canada/BC-BCCDC-6752/2020

hCoV-19/USA/NY-NYCPHL-011383/2022

hCoV-19/USA/NE-CUMC_22094025/2022

hCoV-19/Thailand/NIC_PRE_SEQ9906/2022

hCoV-19/Afganistan/IMB15488/2021

hCoV-19/Crimea/RII-MH2936S/2020

hCoV-19/FrenchPolynesia/IPP35897/2021

hCoV-19/Thailand/NIC_BKK_17237/2022

hCoV-19/Malaysia/A21/2021

hCoV-19/Peru/ARE-INS-16732/2022

hCoV-19/Australia/NSW-SAVID-11098/2022

hCoV-19/Romania/B-3989/2021

hCoV-19/Indonesia/AC-NIHRD-WGS09260/2021

hCoV-19/Singapore/208/2020

hCoV-19/Australia/QLD0x00E0B9/2022

hCoV-19/Denmark/DCGC-1131/2020

hCoV-19/Angola/KRISP-K009705/2020

hCoV-19/CzechRepublic/NRL_11716/2020

hCoV-19/Canada/NL-NML-20652/2021

hCoV-19/Canada/SK-NML-686/2020

hCoV-19/HongKong/VZ22073632/2022

hCoV-19/Canada/SK-NML-16198/2021

hCoV-19/Timor-Leste/TL27/2020

hCoV-19/Kuwait/KU-12024/2021

hCoV-19/Curacao/CW-RIVM-36976/2021

hCoV-19/Palau/PW-CDC-2-5064293/2021

hCoV-19/Aruba/AW-RIVM-97438/2022

hCoV-19/USA/NY-SUNYQB-61210452414265/2022

hCoV-19/Brazil/SP-IB_156267/2022

hCoV-19/Peru/LIM-INS-16628/2022

hCoV-19/SouthAfrica/SU-NHLS_4413/2022

hCoV-19/Israel/ICH-741216957/2022

hCoV-19/USA/NM-CDC-ASC210849113/2022

hCoV-19/Bonaire/BQ-RIVM-96557/2022

hCoV-19/USA/PA-Curative-287882/2022

hCoV-19/Pakistan/NIH-B41-S10/2022

hCoV-19/India/MH-1-31/2020

hCoV-19/India/OR-ILSGS07785/2021

hCoV-19/Australia/ACT0248/2021

hCoV-19/Gambia/0548/2020

hCoV-19/Colombia/DC-INS-3334/2020

hCoV-19/Denmark/DCGC-5323/2020

hCoV-19/Suriname/SR-RIVM-22521/2021

hCoV-19/Latvia/035/2020

hCoV-19/Australia/NT227/2021

hCoV-19/Qatar/QA.QU_18.19.B8/2021

hCoV-19/Qatar/QA.QU_18.19.A1/2021

hCoV-19/Botswana/R21B68_BHP_AAB78372/2021

hCoV-19/Aruba/AW-RIVM-77657/2021

hCoV-19/Peru/LAM-INS-12819/2021

hCoV-19/USA/NY-NYCPHL-001183/2020

hCoV-19/USA/MT-MTPHL-3842245/2021

hCoV-19/SouthSudan/UG1100/2021

hCoV-19/Sweden/20-13056/2020

hCoV-19/Vietnam/PIHCM_224/2022

hCoV-19/Brazil/BA-FIOCRUZ-PVM80967/2021

hCoV-19/Belize/CML-70/2021

hCoV-19/Iran/GRC-81/2020

hCoV-19/Mexico/TAM-INMEGEN-28-246/2021

hCoV-19/Zambia/ZMB-119155/2021

hCoV-19/Ghana/WACCBIP-GS1163/2021

hCoV-19/USA/ID-CDC-ASC210402836/2021

hCoV-19/Germany/ST-RKI-I-171102/2021

hCoV-19/Mexico/JAL-InDRE_FB13207_S1518/2021

hCoV-19/Cambodia/f1208161/2021

hCoV-19/EquatorialGuinea/61992/2021

hCoV-19/Bonaire/BQ-RIVM-77930/2021

hCoV-19/Djibouti/NAMRU3_A06/2020

hCoV-19/Australia/SA1353/2021

hCoV-19/Kenya/SS8012/2022

hCoV-19/Guatemala/INC-LNS-267/2022

hCoV-19/Brazil/BA-FIOCRUZ-PVM99357/2022

hCoV-19/Bonaire/BQ-RIVM-98469/2022

hCoV-19/India/RJ-SMS-ICMR-INSACOG-TS-13659/2022

hCoV-19/Peru/LAL-INS-16712/2022

hCoV-19/Botswana/R1113B31_BHP_1041393/2022

hCoV-19/Switzerland/AG-ETHZ-36894022/2022

hCoV-19/Cambodia/934915/2022

hCoV-19/Oman/rega-OM-86/2021

hCoV-19/EquatorialGuinea/43296/2020

hCoV-19/Denmark/DCGC-88602/2021

hCoV-19/Bulgaria/21BG-NC_A000035/2020

hCoV-19/Peru/LIM-INS-820/2020

hCoV-19/Argentina/INEI109350/2021

hCoV-19/Suriname/SR-465/2021

hCoV-19/Zambia/ZMB-93671/2021

hCoV-19/Mexico/CHP-InDRE-23/2020

hCoV-19/Lithuania/MR-LUHS-Eilnr174/2020

hCoV-19/Mexico/TLA-InDRE-IBT-16/2020

hCoV-19/Panama/GMI-PA641589/2021

hCoV-19/Mongolia/202107061/2021

hCoV-19/Aruba/AW-RIVM-102283/2022

hCoV-19/USA/OK-CDC-QDX37459278/2022

hCoV-19/Brunei/7122118816/2022

hCoV-19/Israel/SMC-7073847/2022

hCoV-19/Sweden/2A580405521/2022

hCoV-19/Mexico/OAX-INMEGEN-74-1/2022

hCoV-19/Aruba/AW-RIVM-102090/2022

hCoV-19/HongKong/VZ22133114/2022

hCoV-19/DominicanRepublic/2017634-LNSPDD/2022

hCoV-19/Panama/M222872-GMI/2022

hCoV-19/USA/OH-CDC-QDX37551102/2022

hCoV-19/USA/OR-OSPHL06170/2022

hCoV-19/Guatemala/4731-LNS/2022

hCoV-19/Brazil/SC-FIOCRUZ-11768/2022

hCoV-19/Mexico/NLE_CIAD-IMSS_202201027193/2022

hCoV-19/Venezuela/Voy2055/2022

hCoV-19/Kyrgyzstan/NRL-43587/2021

hCoV-19/Bonaire/BQ-RIVM-105511/2022

hCoV-19/Curacao/CW-RIVM-106244/2022

hCoV-19/Kenya/MKU-14/2020

hCoV-19/USA/NH-NHPHL-2204290047/2022

hCoV-19/NewZealand/22CH5731/2022

hCoV-19/Zambia/CHAZ-CHS2252/2022

hCoV-19/Zambia/CHAZ-CHS2201/2022

hCoV-19/Australia/VIC58111/2022

hCoV-19/Guadeloupe/GUA-cerba-22T0274091/2022

hCoV-19/Peru/LIM-INS-20646/2022

hCoV-19/Monaco/MCO-cerba-22T0730961/2022

hCoV-19/USA/KY-CDC-LC0748721/2022

hCoV-19/Kazakhstan/378/2022

hCoV-19/Colombia/ANT-CWOHC-VG-SEC03544H/2022

hCoV-19/Taiwan/TSGH-80/2022

hCoV-19/Libya/86043-26/2022

hCoV-19/Mozambique/INS-PXA0175054/2022

hCoV-19/DRC/INRB-RDC-924/2022

hCoV-19/ElSalvador/INC-LNSP-222/2022

hCoV-19/ElSalvador/INC-LNSP-230/2022

hCoV-19/Ghana/WACCBIP-GS3017/2022

hCoV-19/DRC/INRB-RDC-923/2022

hCoV-19/Chile/LL-80674/2022

hCoV-19/NorthMacedonia/IPH-MKD-9582/2022

hCoV-19/Paraguay/450784/2022

hCoV-19/Nigeria/NCDC-NR-GL-005417/2022

hCoV-19/SouthAfrica/NICD-N40286/2022

hCoV-19/SouthKorea/KDCA43563/2022

hCoV-19/Eswatini/N40473/2022

hCoV-19/CostaRica/INC-5611-762301/2022

hCoV-19/Kosovo/CO-00850_XXK000_9447_05_Niph_2022/2022

hCoV-19/Norway/OUS-25382/2022

hCoV-19/Mexico/MIC_LANGEBIO_IMSS_6473/2022

hCoV-19/USA/MS-MSPHL-0911/2022

hCoV-19/Singapore/5533/2022

hCoV-19/CaboVerde/ST5969/2022

hCoV-19/Anhui/WHCDC-011/2022

hCoV-19/Nepal/NPHL-S-1516/2022

hCoV-19/USA/GA-EHC-4362T/2022

hCoV-19/Canada/NL-PHML-433540/2022

hCoV-19/Algeria/20423/2022

hCoV-19/Algeria/2691/2022

hCoV-19/Cambodia/02-2205130256/2022

hCoV-19/Brunei/7122103825/2022

hCoV-19/CostaRica/INC-5665-762516/2022

hCoV-19/Chile/AI-60904/2022

hCoV-19/Argentina/PAIS-G1039/2022

hCoV-19/Netherlands/NH-RIVM-100619/2022

hCoV-19/USA/AR-CDC-2-6071518/2022

hCoV-19/USA/KS-KHEL-11606/2022

hCoV-19/CaboVerde/ST1402/2022

hCoV-19/Suriname/SR-1020/2022

hCoV-19/Ecuador/USFQ-3469/2022

hCoV-19/Georgia/Tb-sNGS4984/2022

hCoV-19/MarshallIslands/MH-H2210100/2022

hCoV-19/Guatemala/8039-LNS/2022

hCoV-19/Bonaire/BQ-RIVM-101149/2022

hCoV-19/Suriname/SR-1038/2022

hCoV-19/USA/CO-CDPHE-2103281710/2022

hCoV-19/Thailand/DMSc-10673/2022

hCoV-19/Thailand/5056207/2022

hCoV-19/Greece/149009/2022

hCoV-19/Australia/SA359715/2022

hCoV-19/Japan/PG-262323/2022

hCoV-19/NewZealand/22CV7053/2022

hCoV-19/Canada/un-NML-411614/2022

hCoV-19/Georgia/Tb-sNGS234/2021

hCoV-19/Lebanon/CMUL-022/2021

hCoV-19/Panama/477371-GMI/2020

hCoV-19/Taiwan/NTU63/2021

hCoV-19/Lesotho/N21459/2021

hCoV-19/Serbia/23-08062022/2022

hCoV-19/Singapore/6485/2022

hCoV-19/Suriname/SR-1049/2022

hCoV-19/Panama/M225885-GMI/2022

hCoV-19/Uganda/494/2022

hCoV-19/Uganda/457/2022

hCoV-19/Brazil/AM-FIOCRUZ-ILMD2204270/2022

hCoV-19/Brunei/9222166511/2022

hCoV-19/Algeria/26943/2022

hCoV-19/Qatar/DA-USAFSAM-S17155/2022

hCoV-19/Laos/LOMWRU-0267/2022

hCoV-19/Ecuador/NIC-INSPI-93849/2022

hCoV-19/Thailand/DMSc-09721/2022

hCoV-19/Morocco/771/2022

hCoV-19/SouthKorea/KDCA72130/2022

hCoV-19/Peru/CUS-INS-18148/2022

hCoV-19/Curacao/CW-RIVM-102734/2022

hCoV-19/NewZealand/22MV2800/2022

hCoV-19/NewZealand/22CV5001/2022

hCoV-19/NewZealand/22CH4089/2022

hCoV-19/NewZealand/22CH4596/2022

hCoV-19/SouthKorea/KDCA77007/2022

hCoV-19/Denmark/DCGC-535329/2022

hCoV-19/Germany/BY-RKI-I-861591/2022

hCoV-19/Paraguay/457410/2022

hCoV-19/DRC/INRB-RDC-690/2022

hCoV-19/Canada/NL-PHML-459391/2022

hCoV-19/Argentina/INEI120782/2022

hCoV-19/Finland/THL-202211854/2022

hCoV-19/Guadeloupe/IPP31330/2022

hCoV-19/Panama/SEQ1211-GMI/2022

hCoV-19/Australia/VIC63351/2022

hCoV-19/Bonaire/BQ-RIVM-103982/2022

hCoV-19/Belize/CML-458/2022

hCoV-19/NewZealand/22CV6570/2022

hCoV-19/Israel/SMC-7087654/2022

hCoV-19/Kuwait/Jaber2220189195/2022

hCoV-19/Ecuador/USFQ-3355/2022

hCoV-19/Zambia/CHAZ-CHS2029/2022

hCoV-19/Georgia/Tb-sNGS5214/2022

hCoV-19/SouthKorea/KDCA91942/2022

hCoV-19/DominicanRepublic/2032655-LNSPDD/2022

hCoV-19/DominicanRepublic/2034110-LNSPDD/2022

hCoV-19/DominicanRepublic/2034835-LNSPDD/2022

hCoV-19/Indonesia/JB-GS-WJHL-ITB-W03923/2022

hCoV-19/Mexico/SIN_IBT_IMSS_025700CIBO_NC/2022

hCoV-19/Zambia/44915/2022

hCoV-19/Zambia/44924/2022

hCoV-19/Oman/7228777/2022

hCoV-19/Oman/7229008/2022

hCoV-19/Oman/7229469/2022

hCoV-19/Mexico/BCS_InDRE_FB16791_E03317458330_S13996/2022

hCoV-19/USA/TN-CDC-QDX38969075/2022

hCoV-19/USA/NJ-CDC-LC0796045/2022

hCoV-19/England/PHEC-YYGMTZR/2022

hCoV-19/Malaysia/IMR_OS1475/2022

hCoV-19/Romania/AG_528601/2022

hCoV-19/Singapore/9913/2022

hCoV-19/SouthAfrica/CERI-KRISP-K045687/2022

hCoV-19/Mauritius/CERI-KRISP-K044303/2022

hCoV-19/USA/OK-CDC-LC0822975/2022

hCoV-19/Namibia/N43487/2022

hCoV-19/Namibia/N43557/2022

hCoV-19/USA/MI-UM-MH28411/2022

hCoV-19/Mauritius/N43349/2022

hCoV-19/Mauritius/N43406/2022

hCoV-19/Liechtenstein/FL-Risch-20220714R12778/2022

hCoV-19/SouthKorea/KDCA96110/2022

hCoV-19/Turkey/HSGM-GS7121/2022

hCoV-19/Australia/QLD0x010AE5/2022

hCoV-19/Guinea/CERFIG-37302/2022

hCoV-19/Brazil/SP-IB_CEVC_2202535/2022

hCoV-19/Gambia/35001T/2022

hCoV-19/Guinea/LFHVG-G0710/2022

hCoV-19/Algeria/33020/2022

hCoV-19/NewZealand/22ZA1804/2022

hCoV-19/NewZealand/22YA1542/2022

hCoV-19/NewZealand/22YA1656/2022

hCoV-19/NewZealand/22YA0458/2022

hCoV-19/NewZealand/22XA0498/2022

hCoV-19/NewZealand/22XA0324/2022

hCoV-19/NewZealand/22ZA2036/2022

hCoV-19/Algeria/33262/2022

hCoV-19/Brazil/ES-LACENES-321281300/2022

hCoV-19/USA/IL-CDC-STM-QVZSVN3FD/2022

hCoV-19/Mexico/SON_LANGEBIO_IMSS_10530/2022

hCoV-19/USA/FL-BPHL-12481/2022

hCoV-19/Peru/JUN-INS-25126/2022

hCoV-19/Saint-Martin/IPP48925/2022

hCoV-19/Guatemala/7879-LNS/2022

hCoV-19/Jordan/s25/2022

hCoV-19/Bonaire/BQ-RIVM-110666/2022

hCoV-19/Bonaire/BQ-RIVM-110673/2022

hCoV-19/Curacao/CW-RIVM-110706/2022

hCoV-19/Curacao/CW-RIVM-110708/2022

hCoV-19/SouthKorea/KDCA106262/2022

hCoV-19/Singapore/10892/2022

hCoV-19/Nepal/NPHL-S-1780/2022

hCoV-19/Venezuela/MirLab11/2022

hCoV-19/SaintLucia/153574/2022

hCoV-19/Barbados/153213/2022

hCoV-19/DominicanRepublic/2055817-LNSPDD/2022

hCoV-19/Chile/AT-UDA-LBMG-7037238398/2022

hCoV-19/Australia/QLD0x011268/2022

hCoV-19/Estonia/20490738/2022

hCoV-19/Aruba/AW-RIVM-110908/2022

hCoV-19/Bonaire/BQ-RIVM-110763/2022

hCoV-19/Curacao/CW-RIVM-111372/2022

hCoV-19/Indonesia/JK-NIHRD-WGS.22.20771/2022

hCoV-19/Zimbabwe/77018/2022

hCoV-19/Argentina/INEI121469/2022

hCoV-19/Argentina/INEI122207/2022

hCoV-19/Jordan/MOH-CPHL003/2022

hCoV-19/Lesotho/N47923/2022

hCoV-19/USA/CT-ASC-210826103/2022

hCoV-19/PuertoRico/PR-ASC-210836041/2022

hCoV-19/Bangladesh/icddrb-TND-06-0989/2022

hCoV-19/Switzerland/TI-EOC-38003814/2022

hCoV-19/SouthAfrica/SU-NHLS_5796/2022

hCoV-19/Germany/BB-RKI-I-970719/2022

hCoV-19/Canada/NB-CHUDGLD-010922-MM00274R/2022

hCoV-19/Syria/Northwest-00349/2022

hCoV-19/Georgia/Tb-sNGS5941/2022

hCoV-19/USA/OH-ASC-210726840/2022

hCoV-19/Brunei/7722011632/2022

hCoV-19/Venezuela/MirLab4/2022

hCoV-19/Tunisia/S929/2022

hCoV-19/Tunisia/55765/2022

hCoV-19/Singapore/11504/2022

hCoV-19/Russia/40-CRIE-7773369236/2022

hCoV-19/PuertoRico/PR-ASC-210830455/2022

hCoV-19/SouthKorea/KDCA121818/2022

hCoV-19/Kenya/ILRI_COVM04374/2022

hCoV-19/Mexico/NLE-LESPNL-00970/2021|EPI_ISL_7716412|2021-03-17

hCoV-19/Mexico/NLE-LESPNL-00971/2021|EPI_ISL_7716413|2021-03-26

hCoV-19/Mexico/NLE-LESPNL-00972/2021|EPI_ISL_7716414|2021-03-29

hCoV-19/Mexico/NLE-LESPNL-00973/2021|EPI_ISL_7716415|2021-03-30

hCoV-19/Mexico/NLE-LESPNL-00975/2021|EPI_ISL_7716416|2021-03-26

hCoV-19/Mexico/NLE-LESPNL-00976/2021|EPI_ISL_7716417|2021-03-26

hCoV-19/Mexico/NLE-LESPNL-00977/2021|EPI_ISL_7716418|2021-03-27

hCoV-19/Mexico/NLE-LESPNL-00978/2021|EPI_ISL_7716419|2021-03-27

hCoV-19/Mexico/NLE-LESPNL-00979/2021|EPI_ISL_7716420|2021-03-27

hCoV-19/Mexico/NLE-LESPNL-00980/2021|EPI_ISL_7716421|2021-03-26

hCoV-19/Mexico/NLE-LESPNL-00981/2021|EPI_ISL_7716422|2021-04-02

hCoV-19/Mexico/NLE-LESPNL-00982/2021|EPI_ISL_7716423|2021-04-01

hCoV-19/Mexico/NLE-LESPNL-00983/2021|EPI_ISL_7716424|2021-04-02

hCoV-19/Mexico/NLE-LESPNL-00985/2021|EPI_ISL_7716426|2021-03-19

hCoV-19/Mexico/NLE-LESPNL-00986/2021|EPI_ISL_7716427|2021-03-20

hCoV-19/Mexico/NLE-LESPNL-00988/2021|EPI_ISL_7716428|2021-03-18

hCoV-19/Mexico/NLE-LESPNL-00989/2021|EPI_ISL_7716429|2021-03-19

hCoV-19/Mexico/NLE-LESPNL-00990/2021|EPI_ISL_7716430|2021-03-21

hCoV-19/Mexico/NLE-LESPNL-00991/2021|EPI_ISL_7716431|2021-03-23

hCoV-19/Mexico/NLE-LESPNL-00992/2021|EPI_ISL_7716432|2021-03-26

hCoV-19/Mexico/NLE-LESPNL-00993/2021|EPI_ISL_7716433|2021-03-26

hCoV-19/Mexico/NLE-LESPNL-00994/2021|EPI_ISL_7716434|2021-03-26

hCoV-19/Mexico/NLE-LESPNL-00996/2021|EPI_ISL_7716436|2021-04-02

hCoV-19/Mexico/NLE-LESPNL-00997/2021|EPI_ISL_7716438|2021-03-25

hCoV-19/Mexico/NLE-LESPNL-00998/2021|EPI_ISL_7716439|2021-03-23

hCoV-19/Mexico/NLE-LESPNL-01000/2021|EPI_ISL_7716440|2021-03-13

hCoV-19/Mexico/NLE-LESPNL-01002/2021|EPI_ISL_7716441|2021-03-13

hCoV-19/Mexico/NLE-LESPNL-01003/2021|EPI_ISL_7716442|2021-03-19

hCoV-19/Mexico/NLE-LESPNL-01004/2021|EPI_ISL_7716443|2021-03-19

hCoV-19/Mexico/NLE-LESPNL-01005/2021|EPI_ISL_7716444|2021-03-19

hCoV-19/Mexico/NLE-LESPNL-01006/2021|EPI_ISL_7716445|2021-04-01

hCoV-19/Mexico/NLE-LESPNL-01007/2021|EPI_ISL_7716446|2021-03-25

hCoV-19/Mexico/NLE-LESPNL-01008/2021|EPI_ISL_7716447|2021-04-03

hCoV-19/Mexico/NLE-LESPNL-01009/2021|EPI_ISL_7716448|2021-04-02

hCoV-19/Mexico/NLE-LESPNL-01010/2021|EPI_ISL_7716449|2021-03-30

hCoV-19/Mexico/NLE-LESPNL-01015/2021|EPI_ISL_7716450|2021-03-25

hCoV-19/Mexico/NLE-LESPNL-01016/2021|EPI_ISL_7716451|2021-04-03

hCoV-19/Mexico/NLE-LESPNL-01017/2021|EPI_ISL_7716452|2021-04-01

hCoV-19/Mexico/NLE-LESPNL-01018/2021|EPI_ISL_7716453|2021-03-19

hCoV-19/Mexico/NLE-LESPNL-01020/2021|EPI_ISL_7716455|2021-03-26

hCoV-19/Mexico/NLE-LESPNL-01021/2021|EPI_ISL_7716456|2021-03-27

hCoV-19/Mexico/NLE-LESPNL-01022/2021|EPI_ISL_7716457|2021-03-31

hCoV-19/Mexico/NLE-LESPNL-01023/2021|EPI_ISL_7716458|2021-04-01

hCoV-19/Mexico/NLE-LESPNL-01024/2021|EPI_ISL_7716459|2021-04-02

hCoV-19/Mexico/NLE-LESPNL-01025/2021|EPI_ISL_7716460|2021-04-02

hCoV-19/Mexico/NLE-LESPNL-01046/2021|EPI_ISL_7716481|2021-04-02

hCoV-19/Mexico/NLE-LESPNL-01047/2021|EPI_ISL_7716482|2021-03-14

hCoV-19/Mexico/NLE-LESPNL-01048/2021|EPI_ISL_7716483|2021-03-14

hCoV-19/Mexico/NLE-LESPNL-01049/2021|EPI_ISL_7716484|2021-03-20

hCoV-19/Mexico/NLE-LESPNL-01050/2021|EPI_ISL_7716485|2021-03-16

hCoV-19/Mexico/NLE-LESPNL-01051/2021|EPI_ISL_7716486|2021-03-17

hCoV-19/Mexico/NLE-LESPNL-01052/2021|EPI_ISL_7716487|2021-03-18

hCoV-19/Mexico/NLE-LESPNL-01053/2021|EPI_ISL_7716488|2021-03-18

hCoV-19/Mexico/NLE-LESPNL-01054/2021|EPI_ISL_7716489|2021-03-19

hCoV-19/Mexico/NLE-LESPNL-001055/2021|EPI_ISL_7730969|2021-03-26
