# Supplementary material for: A Proofreading Mutation with an Allosteric Effect Allows a Cluster of SARS-CoV-2 Viruses to Rapidly Evolve
Source: Mol Biol Evol. 2023 Sep 20;40(10):msad209. doi: 10.1093/molbev/msad209 (PMC10553922; doi:10.1093/molbev/msad209)
Supplement: msad209_Supplementary_Data [file msad209_supplementary_data.zip › NSP14 Mutation and accelerated evolution manuscript supplementary figures and tables for publication.pdf]

Dataset	PANGO lineage	Number of sequences	Sampling country	Sampling window (days)
Q22H	AY.4	100	England	205
T25L	B	24	China	35
H26N	AY.122	24	Wales	40
H26Y	AY.4	100	Wales	216
C39F	B.1.1.7	54	England	35
M57I	AY.4	20	Wales	21
M57V	AY.1.28	24	England	42
F60S	B.1.153	55	Mexico	22
M62I	AY.4	102	England	216
M62T	B.1.1.7	100	Denmark	80
M62V	B.1.1.7	29	France	107
M195I	BA.2	54	England	47
M195T	AY.4	55	Wales	41
I201M	AY.4	112	England	65
Control 1	B.1.1	100	Wales	288
Control 2	B.1.1.41	77	England	102

Supplementary Table 1. Details of each dataset obtained from GISAID including country of origin, PANGO lineage, number of sequences and timescale of sampling window.

Dataset	Slope (rate)	X-Intercept (TMRCA)	Correlation Coefficient	R^2
Q22H	6.8738E-04	2021.1682	0.6249	0.3905
T25L	3.7066E-04	2019.7674	0.3106	0.096451
H26N	1.8632E-04	2021.6988	0.3633	0.132
H26Y	4.1996E-04	2021.0308	0.7423	0.5511
C39F	2.1912E-04	2020.0661	0.1846	0.034092
M57I	8.5108E-04	2021.3685	0.7822	0.6118
M57V	1.9169E-04	2021.6672	0.2385	0.05688
F60S	2.6328E-03	2021.16	0.088156	0.0077715
M62I	9.2899E-04	2021.2796	0.9119	0.8316
M62T	4.8369E-04	2021.2583	0.6871	0.4721
M62V	6.3744E-04	2021.0289	0.6298	0.3966
M195I	4.377E-04	2021.9175	0.3665	0.1344
M195T	6.6911E-04	2021.4159	0.5282	0.279
I201M	4.3271E-04	2021.6851	0.3612	0.1305
Control 1	3.3262E-04	2019.9573	0.6641	0.4411
Control 2	6.208E-04	2020.1265	0.7488	0.5607

Supplementary Table 2. The results of the root-to-tip regression for each dataset showing their relative slope, time to most recent common ancestor (TMRCA), correlation coefficient and R^2.

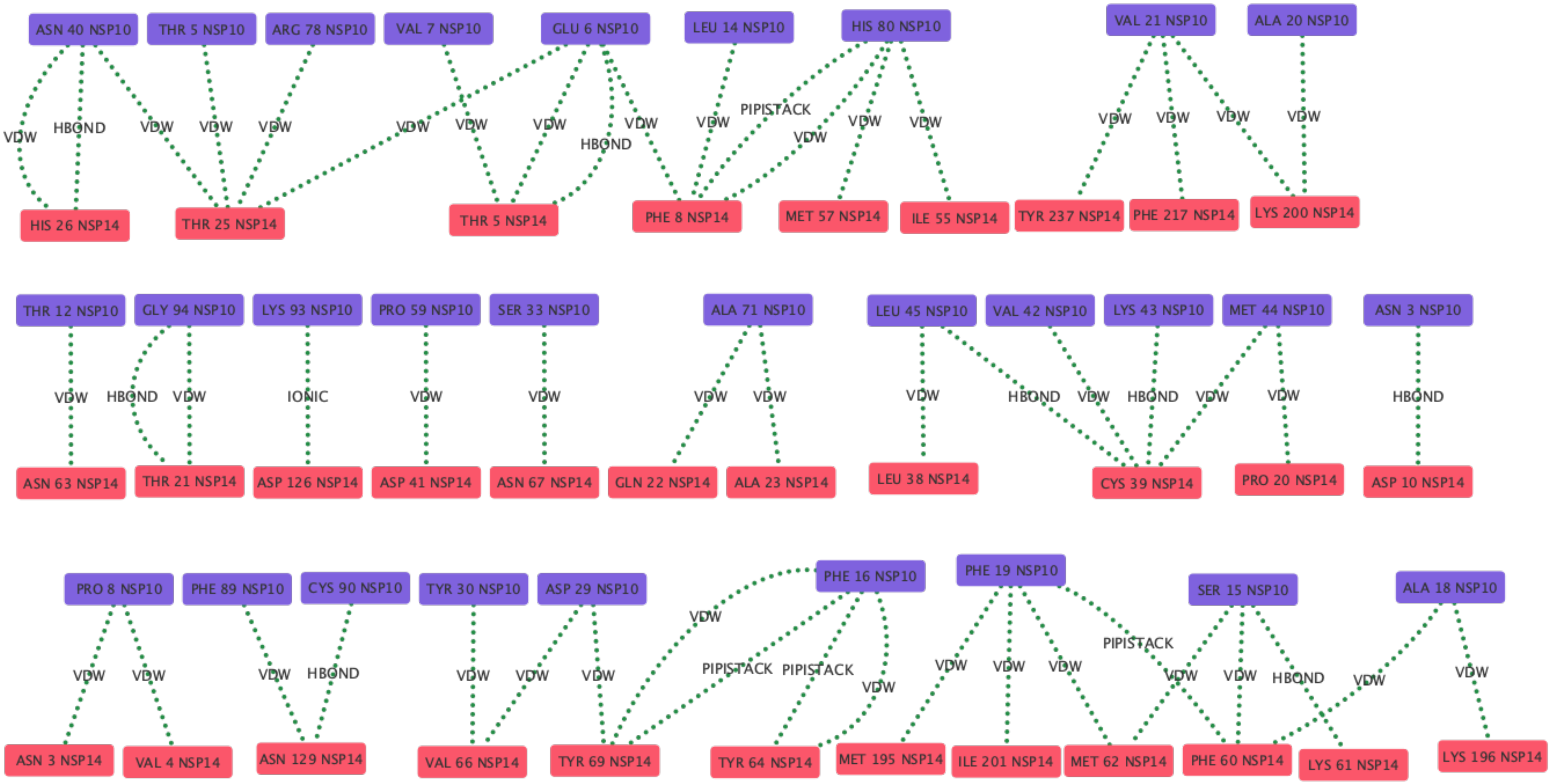

Supplementary Figure 1. Interfacial Residues of the NSP14/NSP10 Exonuclease Complex in SARS-CoV-2. This representation illustrates the interface residues between NSP14 (red) and NSP10 (blue) of the exonuclease complex and their respective interaction types. VDW – Van der Waals interactions, HBONDS – hydrogen bonds, PIPISTACK – Pi Pi stacking, IONIC – ionic bond.

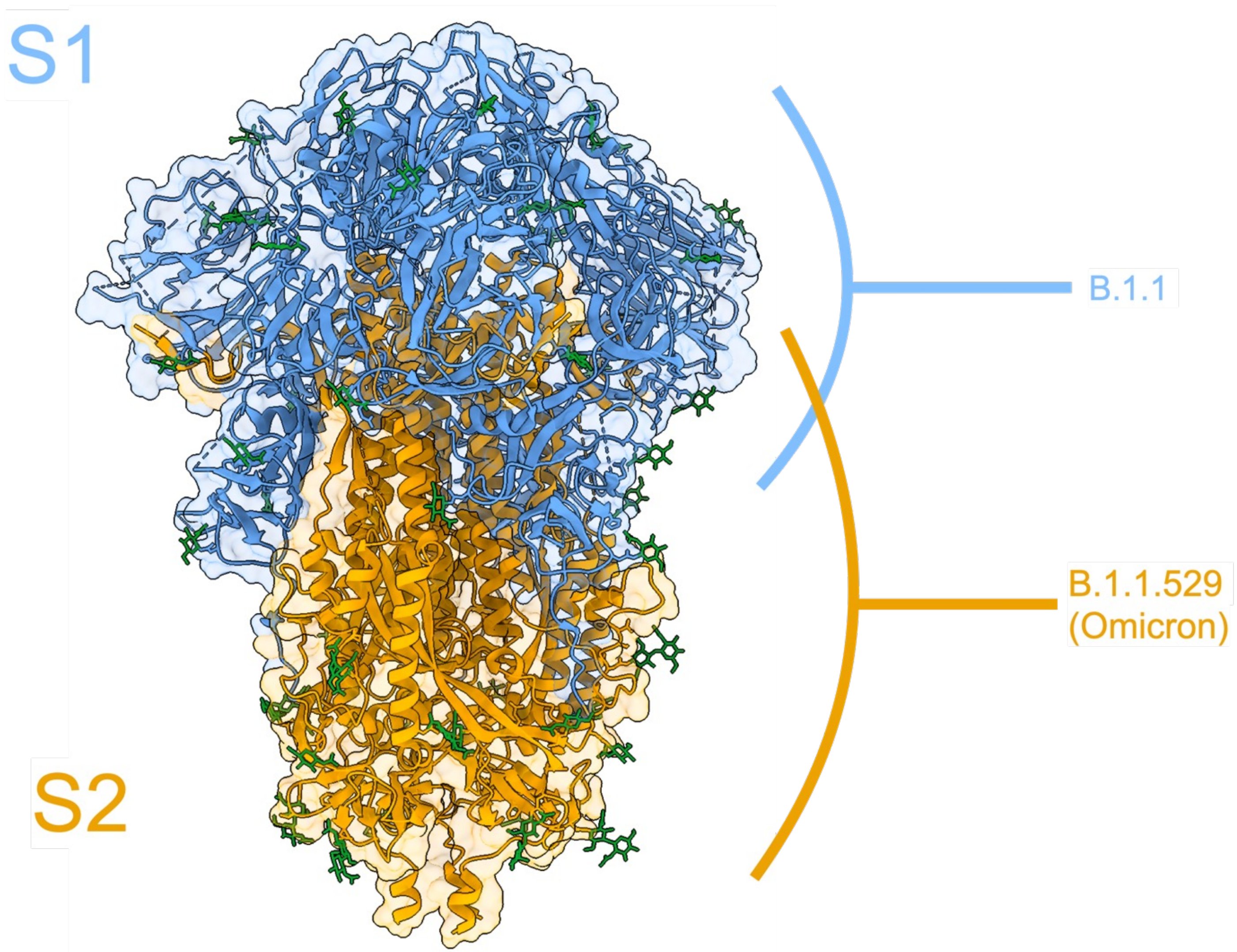

Supplementary Figure 2. An illustration of the recombinant spike protein of the F60S harbouring mutant lineage. Here, the S1 domain deriving from B.1.1 is coloured blue whilst the S2 domain deriving from the B.1.1.529 (Omicron) lineage is coloured orange. Base structure is the glycosylated SARS-CoV-2 spike protein in the closed state PDB:6VXX

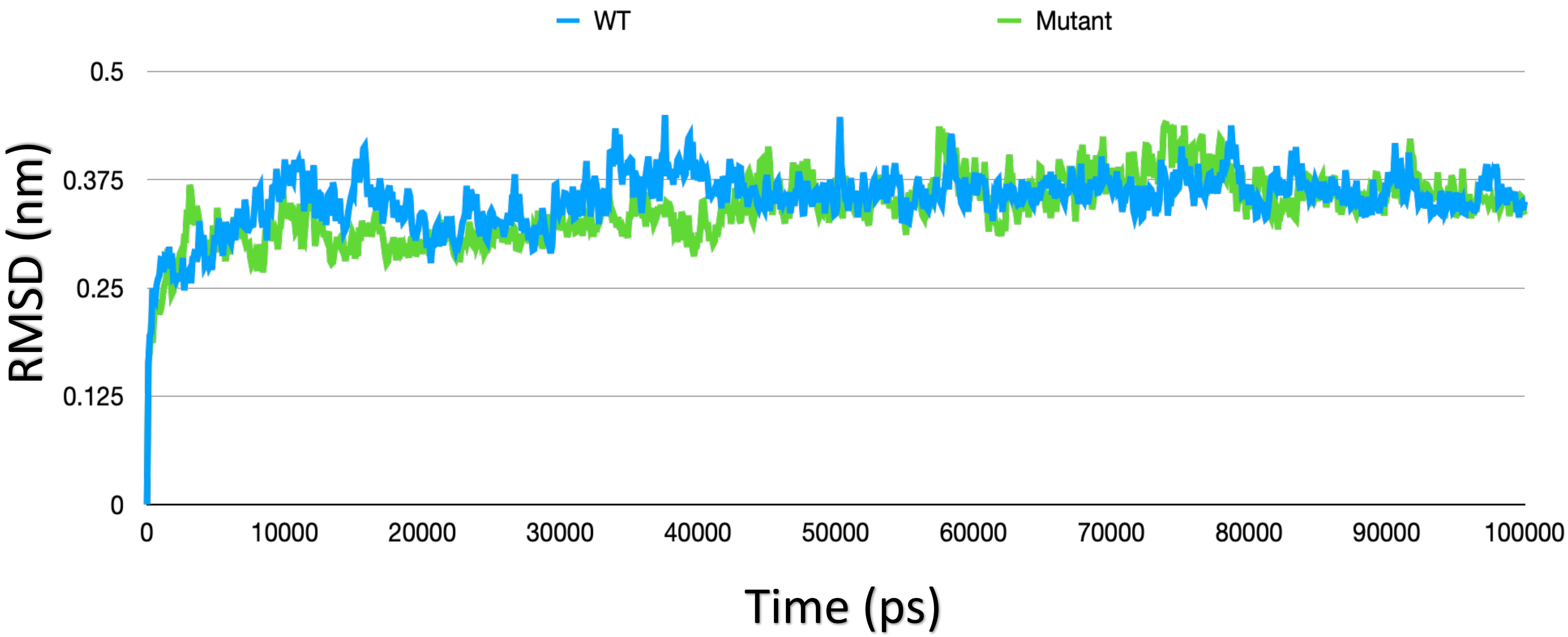

Supplementary Figure 3. The average root-mean squared deviation of 3 x 100ns simulations of WT and F60S mutant NSP14/NSP10 complex.

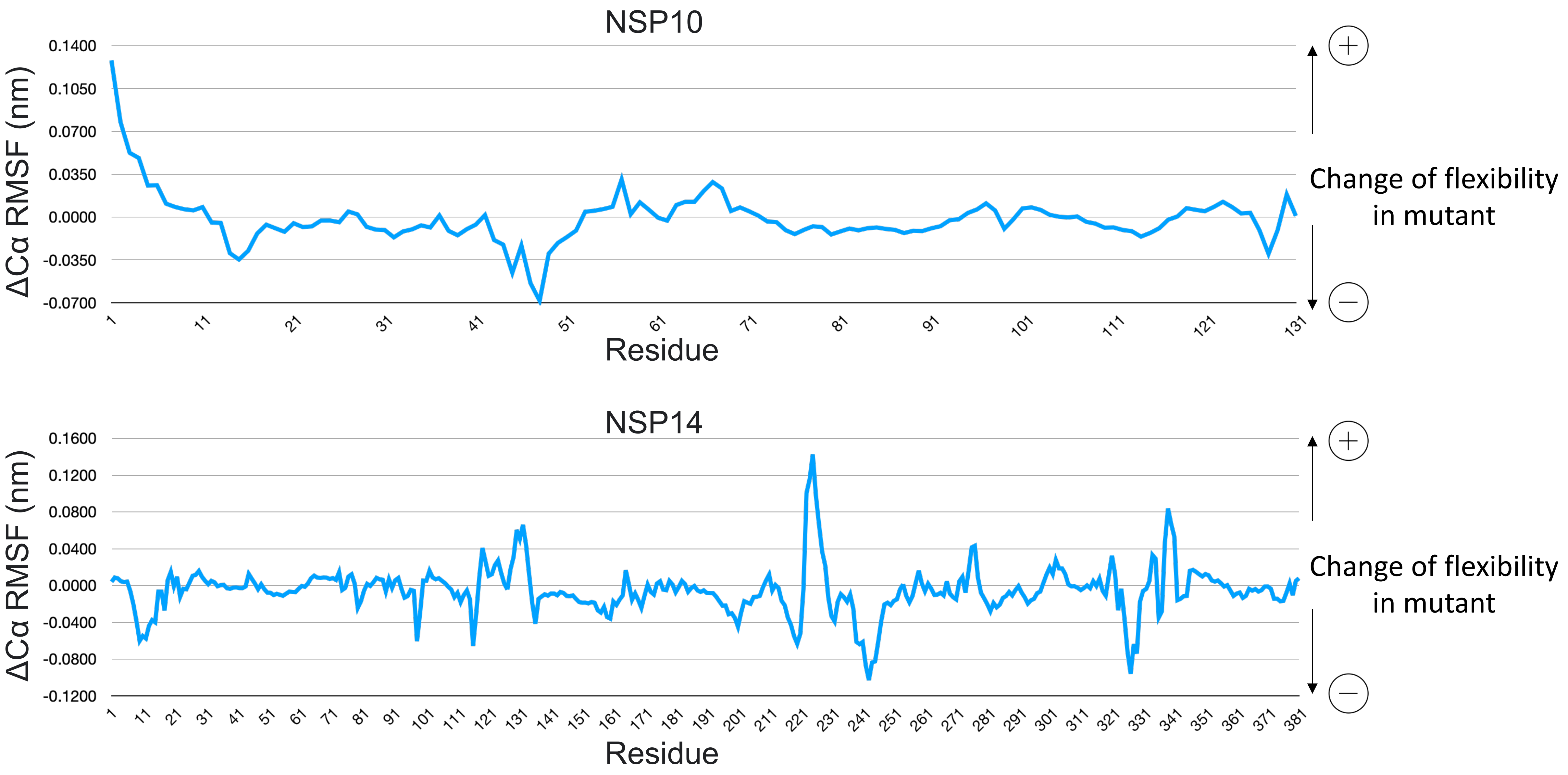

Supplementary Figure 4. The change in the root mean squared fluctuations (RMSF) of the Cα observed between the WT and F60S mutant, with the the results for NSP10 (top) and NSP14 (bottom) being shown independently.

Dataset	S SS	S PS	LN SS	LN PS	EX SS	EX PS
H26N	-40282.4118504875	-40282.4751161993	-40284.5938113324	-40284.6050647465	-40282.4129858475	-40282.364608033
T25L	-40602.7472999046	-40602.6932935566	-40606.6579862221	-40606.6831656147	-40601.0213733854	-40601.0403587682
C39F	-40575.9090279387	-40575.2429791334	-40564.7476532469	-40564.2296096151	-40571.3957624052	-40570.7469680385
Q22H	-43294.1030359266	-43291.0705611925	-43288.6283490547	-43285.1500258645	-43270.1418259602	-43267.6251200548
H26Y	-41701.0691854795	-41702.7001760196	-41699.4669916686	-41698.0488919814	-41699.7601465086	-41698.4183363402
M57I	-40502.2562539544	-40502.0292515868	-40493.2042859832	-40493.1864641813	-40499.6981694486	-40499.5013340429
M57V	-40334.1543223434	-40334.1457649401	-40333.4126665708	-40333.5291626146	-40333.8194950958	-40333.8045501942
M62I	-43118.0482487614	-43116.5097323732	-43109.3694207046	-43107.1207542222	-43110.6099522088	-43108.1889516005
M62T	-40791.6341213419	-40790.6348578999	-40790.6348578999	-40792.2999251239	-40789.0363263004	-40788.5620846824
M62V	-41588.3946551412	-41588.154457321	-41597.0488840093	-41596.9609436513	-41589.9618397245	-41589.8785907333
F60S	-50872.7313186001	-50871.7123293947	-50726.7868300091	-50725.9018609837	-50717.6722813595	-50716.5207688267
M195I	-40715.4895575776	-40714.972908385	-40720.6130421162	-40720.2412686625	-40713.6677335367	-40713.5508427863
M195T	-40653.2867624192	-40652.9272359425	-40656.2609170359	-40655.8706132304	-40651.2241347799	-40651.017859031
I201M	-40517.989359347	-40517.7386989085	-40514.9290330853	-40514.8497555012	-40512.366446474	-40512.1688998947
Control 1 B.1.1	-41554.4028158521	-41552.2947124363	-41556.3893877065	-41554.5727112081	-41560.220993373	-41558.3362403893
Control 2 B.1.1.41	-41221.9733234935	-41220.6842235797	-41227.6249348843	-41226.7631212406	-41223.0470974952	-41222.2582461609

Supplementary Table 3. Results of stepping-stone (SS) and path sampling (PS) analysis to identify the most appropriate model for Bayesian analysis. S – Strict clock model, LN – Log-normal clock model, EX – Exponential clock model.

Breakpoints		Sequences			Method (p-value)								
Begin	End	Recombinant Sequence(s)	Minor Parental Sequence(s)	Major Parental Sequence(s)	RDP	GENECOV	Bootscan	Maxchi	Chimaera	SiSscan	PhylPro	LARD	3Seq
21742	23666	hCoV-19/Mexico/NLE-LESPNL-01015/2021|EPI_ISL_77164	hCoV-19/Canada/BC-BCCDC-6752/2020	Unknown (hCoV-19/Australia/SA1353/2021)	0.00094	0.000120	0.000058	0.028000	0.034000	NS	NS	NS	0.008500

Supplementary Table 4. Here the results of the recombination analysis from RDP5 are shown. 6/9 methods from RDP5 support the presence of a recombination signal within these sequences, specifying a breakpoint from nt21742 to nt23666 which corresponds to residues 61 to 705 of the spike protein. This table is representative of all F60S sequences within the dataset.

Method	Affinity Change ΔΔG	Stabilising/Destabilising
DynaMut2	-3.32 kcal/mol	Destabilising
I-Mutant2.0	-1.67 kcal/mol	Destabilising
CUPSAT	-1.12 kcal/mol	Destabilising
mCSM-PPI2	-1.635 kcal/mol	Destabilising
mCSM	-2.617 kcal/mol	Destabilising

Supplementary Table 5. Results from the 5 different, structure-based approaches to determine the effect of the F60S mutation on binding energies and complex stability.

<u>Distribution of rotomer conformations from MD simulations</u>

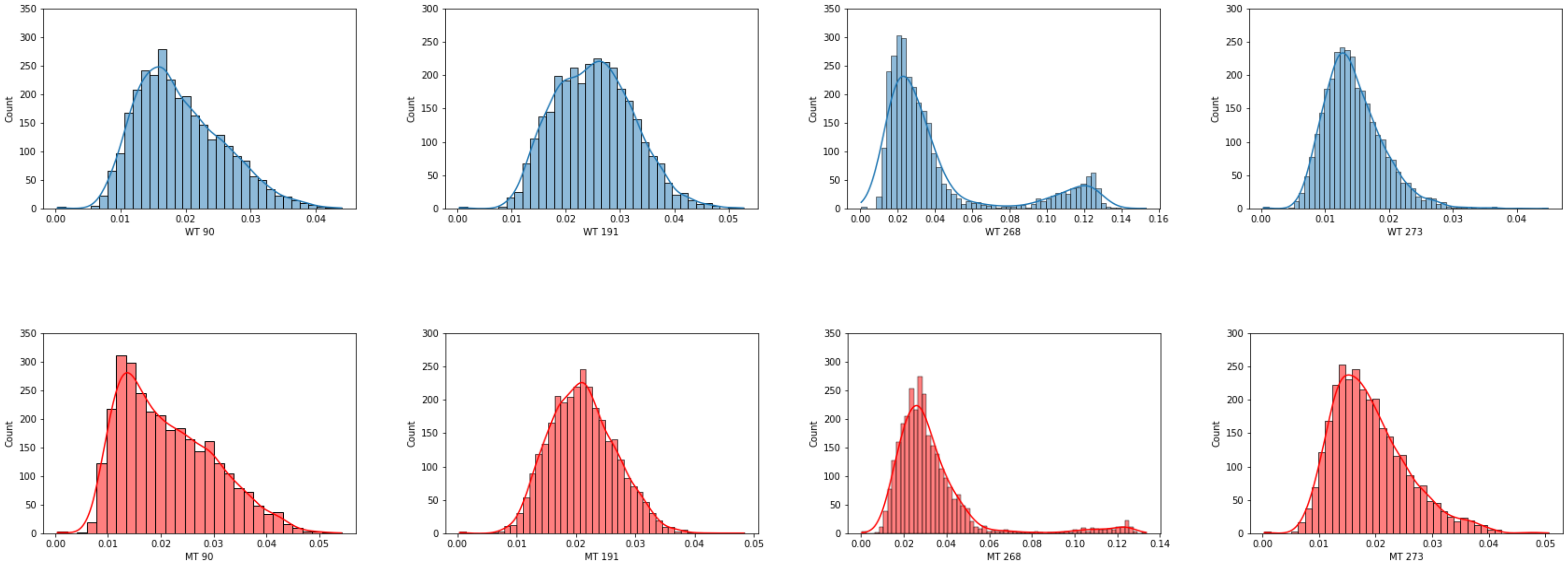

Supplementary Figure 5. The population distribution of root-mean squared deviation (RMSD) of the D90, E191, H268 and D273 catalytic residues of the NSP14 from 3 x 100ns MD simulations.

Dataset	BEST MODEL SS	BEST MODEL PS	BEST MODEL - NO TIP DATES SS	BEST MODEL - NO TIP DATES PS	SS Bayes factor	PS Bayes factor
H26N	-40282.41299	-40282.36461	-40282.63045	-40282.64922	0.284610086	0.284610086
T25L	-40601.02137	-40601.04036	-40603.44621	-40603.44548	2.40511684	2.40511684
C39F	-40564.74765	-40564.22961	-40585.02854	-40585.03149	20.80188008	20.80188008
Q22H	-43270.14183	-43267.62512	-43333.06954	-43332.92599	65.30086606	65.30086606
H26Y	-41699.46699	-41698.04889	-41804.84827	-41804.66923	106.6203417	106.6203417
M57I	-40493.20429	-40493.18646	-40493.60946	-40493.70642	0.519954101	0.519954101
M57V	-40333.41267	-40333.52916	-40333.51827	-40333.53043	0.001262655	0.001262655
M62I	-43109.36942	-43107.12075	-43182.32755	-43182.21209	75.09133141	75.09133141
M62T	-40789.03633	-40788.56208	-40833.2782	-40833.18728	44.62519281	44.62519281
M62V	-41588.39466	-41588.15446	-40728.46572	-40728.47221	-859.6822502	-859.6822502
F60S	-50717.67228	-50716.52077	-50729.88685	-50729.79551	13.27474354	13.27474354
M195I	-40713.66773	-40713.55084	-40729.57359	-40729.5905	16.03965356	16.03965356
M195T	-40651.22413	-40651.01786	-40658.84439	-40658.81669	7.798828939	7.798828939
I201M	-40512.36645	-40512.1689	-41060.88273	-41060.76962	548.6007182	548.6007182
Control 1 B.1.1	-41554.40282	-41552.29471	-41617.94817	-41617.89407	65.59935419	65.59935419
Control 2 B.1.1.41	-41221.97332	-41220.68422	-41279.25481	-41279.16546	58.48123784	58.48123784

Supplementary Table 6. Results of the BETS analyses showing the difference in Log marginal likelihood (Bayes factor) between the best model, and the best model without sampling/tip dates included . SS – stepping stone, PS – path sampling.

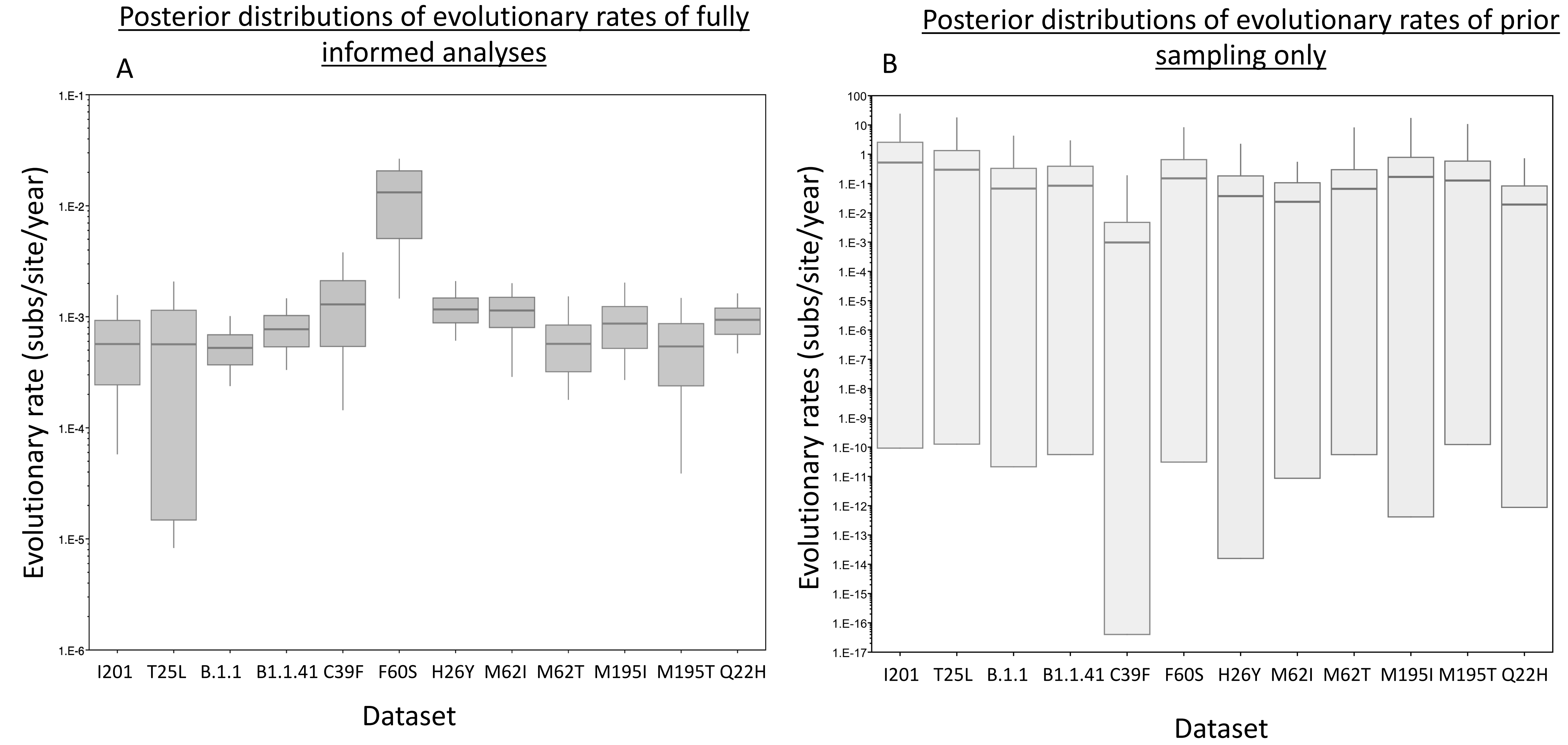

Supplementary Figure 6A The posterior distribution of the evolutionary rate (substitutions/site per/year) of each dataset analyzed under the best ranking model. 6B Posterior distribution of the evolutionary rates (subs/sites/years) from the BEAST analysis of each dataset, where only the prior was sampled.

aa_mutation	sample_count	sample_frequency	category	aa_mutation	sample_count	sample_frequency	category
S:E484K	3	0.054545	VoC	S:A688V	1	0.018182	spike
S:N501Y	1	0.018182	VoC	S:V722D	52	0.945455	spike
S:L452R	4	0.072727	VoC	S:M731L	49	0.890909	spike
S:A348S	2	0.036364	escape	S:T732A	1	0.018182	spike
S:T376I	1	0.018182	escape	S:N751D	1	0.018182	spike
S:K417T	2	0.036364	escape	S:G769R	1	0.018182	spike
S:T478K	36	0.654545	escape	S:V781D	17	0.309091	spike
S:G413V	6	0.109091	RBD	S:P812T	51	0.927273	spike
S:A520S	3	0.054545	RBD	S:A845S	1	0.018182	spike
S:P681H	2	0.036364	cleavage	S:I870T	52	0.945455	spike
S:T20I	2	0.036364	spike	S:M900L	3	0.054545	spike
S:P25S	49	0.890909	spike	S:K921E	12	0.218182	spike
S:S46T	1	0.018182	spike	S:D950E	44	0.8	spike
S:G75D	3	0.054545	spike	S:T1027I	1	0.018182	spike
S:T76I	1	0.018182	spike	S:E1031K	53	0.963636	spike
S:F106I	10	0.181818	spike	S:S1037L	54	0.981818	spike
S:N137S	1	0.018182	spike	S:V1122M	1	0.018182	spike
S:D138Y	2	0.036364	spike	S:V1128L	54	0.981818	spike
S:W152C	4	0.072727	spike	S:K1157N	6	0.109091	spike
S:M153T	1	0.018182	spike	S:V1176F	1	0.018182	spike
S:A163T	6	0.109091	spike	S:K1181Q	1	0.018182	spike
S:F168S	19	0.345455	spike	S:Q1208K	6	0.109091	spike
S:R190S	1	0.018182	spike	S:M1237K	54	0.981818	spike
S:I197T	5	0.090909	spike	S:D614G	52	0.945455	D614G
S:G261V	2	0.036364	spike	S:Q644L	8	0.145455	spike
S:E281Q	1	0.018182	spike	S:H655Y	3	0.054545	spike
S:F559I	47	0.854545	spike	S:A672S	42	0.763636	spike
S:A570D	1	0.018182	spike				

Supplementary Table 7. All mutations of the SARS-CoV-2 Spike protein, and their frequency within the F60S dataset. "category" column details whether the mutation is associated with a variant of concern (VoC), Immune escape (escape), receptor binding domain (RBD) or modulation of the cleavage site (cleavage). Mutations of unknown significance are noted as "Spike". Results were obtained from the USHER analysis.
